# Simple tools for assembling and searching high-density picolitre pyrophosphate sequence data

**DOI:** 10.1186/1751-0473-3-5

**Published:** 2008-04-18

**Authors:** Nicolas J Parker, Andrew G Parker

**Affiliations:** 1 10 Lockhart Close, Kenilworth, Warwickshire, CV8 1RB, UK; 2 Entomology Unit, FAO/IAEA Agriculture and Biotechnology Laboratory, Agency's Laboratories Seibersdorf, International Atomic Energy Agency, A-1400 Vienna, Austria

## Abstract

**Background:**

The advent of pyrophosphate sequencing makes large volumes of sequencing data available at a lower cost than previously possible. However, the short read lengths are difficult to assemble and the large dataset is difficult to handle. During the sequencing of a virus from the tsetse fly, *Glossina pallidipes*, we found the need for tools to search quickly a set of reads for near exact text matches.

**Methods:**

A set of tools is provided to search a large data set of pyrophosphate sequence reads under a "live" CD version of Linux on a standard PC that can be used by anyone without prior knowledge of Linux and without having to install a Linux setup on the computer. The tools permit short lengths of *de novo *assembly, checking of existing assembled sequences, selection and display of reads from the data set and gathering counts of sequences in the reads.

**Results:**

Demonstrations are given of the use of the tools to help with checking an assembly against the fragment data set; investigating homopolymer lengths, repeat regions and polymorphisms; and resolving inserted bases caused by incomplete chain extension.

**Conclusion:**

The additional information contained in a pyrophosphate sequencing data set beyond a basic assembly is difficult to access due to a lack of tools. The set of simple tools presented here would allow anyone with basic computer skills and a standard PC to access this information.

## Background

The introduction of micro-fabricated high-density picolitre reactor pyrophosphate sequencing [1,2] by the company 454 Life Sciences (454 Life Sciences Corp., 20 Commercial Street, Branford, Connecticut 06405, USA; hereafter referred to as 454 sequencing) makes available for the first time large quantities of sequence data at reasonable cost. The continual reduction in sequencing cost will encourage sequencing by small groups or individual researchers with modest computer resources and limited experience of bioinformatics tools.

The nature of the data from pyrophosphate sequencing is however both qualitatively and quantitatively different from that generated by Sanger sequencing [3] using fluorescent chain-terminating nucleotide analogues [4]. Instead of receiving a single consensus sequence with associated chromatogram (scf) file, this form of pyrophosphate sequencing generated for us 300 000 short sequence reads, around 100 bases long [5], assembled into several hundred contigs. However the normal system of checking dubious base calls on the chromatogram is not available, the qual and other associated files were not recognized by standard assembly tools (Phred/Phrap, DNAStar, NTiVector etc.) at the time we received the data (the ace file format has subsequently been made compatible with consed) and the very large number of short reads are difficult to assemble [6]. Assemblers designed for short reads are available [7-9] that utilize the de-Bruijn graph method [10] but they still suffer from shortcomings that result in the production of a large number of short contigs. Assembly is improved by the use of paired-end ditags [11], but this requires an additional sequencing run, thereby doubling the cost and does not necessarily resolve all the problems with repeat regions [12]. The additional cost though may be acceptable when compared to the cost of finishing by hand. The assemblers are also sensitive to read errors which generally occur at higher frequency in pyrophosphate sequencing than in Sanger sequencing [1] so the assembly protocol often incorporates an error correction step that corrects or removes sequences with low frequency [7,13]. This approach is acceptable when the source material is clonal and to produce a first consensus assembly, but when it is not clonal this error correction will mask polymorphisms in the source material. The pyrophosphate sequencing technique is still developing and both the average read length (now about 230 bases) and the number of reads returned per run (now around 500 000) will continue to increase, making this technique more attractive and the need for suitable tools greater.

Recently a novel DNA virus from the tsetse fly *Glossina pallidipes *(Diptera: Glossinidae) [14,15] (salivary gland hypertrophy virus, GpSGHV) has been sequenced by 454 sequencing; at this time paired-end ditag reads were not offered. The source material was not clonal, having been purified directly from infected salivary glands. The purified DNA was divided into two and submitted to 454 as two independent samples run together on a single reaction slide. After sequencing and assembling 454 were informed that the samples were from the same source and they were requested to assemble the combined fragment databases. The virus has a size of about 190 000 bases and the sequencing from the combined fragment database generated 321 953 reads averaging 108.8 bases each for a total of 35 023 571 bases read, giving about 185-fold coverage. These reads were assembled into 402 contigs by the Newbler software at 454. Prior to the pyrophosphate sequencing a number of clones had been sequenced by Sanger sequencing and subsequently targeted PCR amplification and sequencing were used for gap filling. However, it was realised that there was considerable additional information in the fragment database supplied by 454 if it could be suitably analysed.

Most individuals or small groups tempted by the low cost of pyrophosphate sequencing will probably use Windows based PCs. However, after the initial assembling, finishing the sequence and extracting additional information from the large database of individual reads presents a challenge when using Windows based programs. This is because the large datasets are too big to manipulate easily with standard Windows programs. There is, therefore, a clear need for assembly tools for this type of data that will permit detailed interrogation of the full dataset, but in the meantime we offer some simple tools that can be used to help to manually correct the problems encountered with the existing assembly programs and to investigate repeats and polymorphisms. We also discuss a number of issues related to the pyrophosphate sequencing revealed by examining our dataset.

## Methods

The simple tools described here provided the ability to search a database of more than 300 000 fragments for matches, collect the fragments, sort and count them, count the bases adjacent to the match and to perform a simple assembly and associated functions. To produce these tools easily and to make them open source they are based on ISO standard C code compiled under GNU Linux and script files running standard GNU Linux utilities. To make them readily accessible to users of Windows systems unfamiliar with GNU Linux instructions are provided for running the tools using the Knoppix "live" CD [16]. This allows Debian GNU Linux to be run on any PC with a bootable CD or DVD drive without making any changes to the Windows installation. The Knoppix GNU Linux does not use the PC hard disk drive, leaving the original Windows system completely unchanged. The tools have been tested under Knoppix 5.1, Red Hat 9 and Fedora 7 on several 32-bit PCs.

The current native disk format from Windows 2000 (NTFS) cannot be reliably written by some versions of Linux so we recommend a USB flash memory drive in order to have storage available that is accessible both to the Linux system and to Windows. An external hard disk with a FAT32 partition or a FAT32 partition on the internal hard disk can also be used. Instructions for setting up the necessary file structure and running the tools that should allow anyone reasonably familiar with a Windows system to run the Knoppix system and use these tools is provided in Additional file 1. The supplementary material consists of three files, the instructions [see Additonal file 1], the sample files, source code and compiled objects for the tools [see Additonal file 2], and the supplementary tables [see Additional file 3]. The latest versions are available from our web page [17].

### The tools

Performance figures are given for the tools run under Knoppix DVD 5.1 on an Intel Pentium 4, 1.5 GHz, with 256 kB cache and 512 MB RAM with the dataset already loaded into memory. Initial loading of the database takes about 30 seconds, and performance is dramatically reduced if the dataset needs to be reloaded into memory. As the tools all run in memory, disk or flash memory access speed is not an issue, affecting only the first loading of the database so long as the memory size is large enough to accommodate the dataset and any programs being run. For this reason, a minimum of 384 MB RAM is recommended. Unless otherwise stated the performance figures are based on the search string ACTGCTAAGTAATTTGTGAA, which produces 102 matching fragments with our full dataset of 331 953 fragments. The time taken for the tools is linear with respect to database size and to the number of matches produced.

The tools consist of the following.

fna_to_fnb *<input_file >output_file*

fna_to_fnb converts the data file (fna) supplied by 454 into a format usable by these tools. The fna file is in fasta format and is converted by removing the line break between each header and sequence so that they are on one line to make searching easier and quicker. The header lines are converted to lower case, to distinguish them from the following sequence, which is converted to uppercase. See the supplementary material *pyrophosphate_readme.pdf *for further details.

build *primer_file output_file*

This routine takes a primer string (minimum 60 bases, maximum 250 bases) from the file *primer_file *and searches the database for exact matches to a 60 base string, forward and complement. The consensus base for position 61 is determined from the collected fragments, it is then added to the primer string, and the 60 base match frame moved forward one place. After 60 bases the result is written to *output_file *as a single line, with a progress message written to standard error. If the number of matching fragments falls below 8, or the proportion that the fragments containing the consensus base to all matches of the 60 base primer string is less than 70% (as at the end of a repeated sequence where the sequence becomes unique again) the program stops, writing the remaining data out and displaying the problem. The problem can then be further investigated using other tools. If the input primer string is more than 60 bases, the bases up to the last 60 are written to standard out before starting the search. The tool stops automatically after 1920 bases if not stopped before this by a mismatch, in order to avoid being caught in an endless loop.

**Figure 1:**
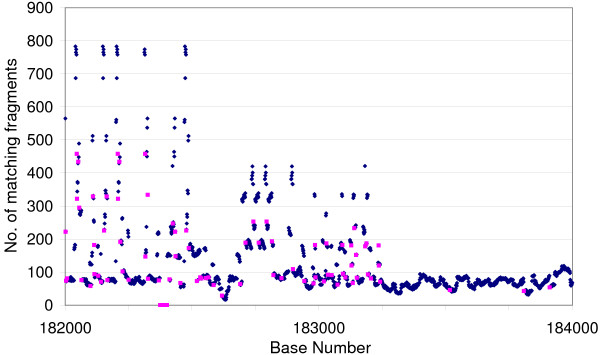
**Graphical representation of the output from re**. The sequence of red points on the x-axis near 182400 represents a mismatch region where a single base was inserted by the assembler. The areas with many more reads than the average show identical repeats. The match length was set to 27 for this Fig.

This tool searches only for exact matches to the 60 base string in the primer. As the dataset is so large, at each position there will typically be 20 – 40 matching fragments. When the dataset is smaller it will fail more quickly, and as the average number of matches approaches the limit of 8 it will not work. Exact repeats of more than 60 bases will result in a loop being formed that has to be resolved with other tools. 1920 bases are assembled in about 23 minutes.

re [*match_length*]* input_file output_file*

re takes an existing draft sequence and checks that the draft is supported by the fragment dataset at each base position. There are two mandatory arguments; the input and output file names. The tool ignores all characters in the input file except uppercase A, C, G and T, so it will accept a standard FASTA format file. The output file is in fixed width text format, and can be loaded into other (Linux or Windows) programs, such as Excel, for further processing. The first parameter is optional; if present it specifies the match length of the search, if not present a default length of 65 bases is used.

re takes *match_length *bases of the *input_file *and counts the number of reads containing *match_string *with the immediately preceding base and the number containing *match_string *plus the immediately succeeding base. The output is in the form of a table (Supplementary Table 1; see Additional file 3). The third column is the *match_string*, the second column is the immediately preceding base and the first column is the position of this base in the sequence being re-built. The fourth column is the immediately succeeding base. Column 5 is the number of matches to *match_string *plus the preceding base, and column 6 is the percentage that column 5 represents of all matches to *match_string *with any preceding base (but not N). Columns 6 and 7 likewise present the data for *match_string *with the succeeding base. In the succeeding line the *match_string *is stepped on one base and the searches repeated. This output can easily be further processed in a spreadsheet to display the number of matches, with matches below a threshold highlighted (Fig. 1) indicating that further investigation is required. An abrupt drop to zero matches identifies a base error, an insert, deletion or substitution and a sudden increase in matches above the general level identifies an exact repeat region. re takes about 6 minutes 30 seconds to check a 4 100 base sequence with the default 65 base match.

fr *match_string output_file*

fr takes any length string from standard in and outputs all fragments with an exact match from the dataset to *output_file*. If *output_file *is omitted the output is sent to standard out. Reverse matches are also output, complemented to bring them into the same sense as the original *match_string*. The original *match_string *is included at the beginning of the output (Supplementary Table 2; see Additional file 3). fr takes about 1.3 seconds.

fnb_to_fas *input_file *>*output_file*

fnb_to_fas takes a file produced by fr and converts it into standard FASTA format for further processing in an assembler or other program, sending the output to standard out unless redirected. If no parameter is supplied it takes input from standard in (keyboard) until terminated by <Ctrl>-d and outputs to standard out. fnb_to_fas takes about 30 seconds to convert 100 000 fragments to FASTA format.

fr2 *match_string output_file*

fr2 collects fragments as fr, but then truncates the beginning of each fragment to the first character of *match_string *and performs a simple alphabetic ascending sort to simplify visual comparison of the fragments. The forward and reverse matches are output separately, identified with > and < respectively and separated by one line (Supplementary Table 3; see Additional file 3). A related function, frx, produces the same output but with the *match_string *replaced by <x> to make it easier to view longer sequences. fr2 takes about 1.3 seconds.

count *match_string*

count searches the dataset for exact matches to *match_string *and outputs two numbers, the forward and reverse match number. count takes about 1 second.

c4 *match_string*

c4 collects exact matches to *match_string *in the dataset and counts the number of occurrences of each of the four bases in the position immediately following *match_string*. The output is written to standard out and consists of twelve lines with *match_string *followed by each of the bases in the order A, C, G, T and the count of forward and reverse matches. c4 takes about 3 seconds.

c4p *match_string*

c4p collects the same information as c4 but with each of the four bases in the position immediately preceding *match_string*. The output is written to standard out and consists of twelve lines with *match_string *preceded by each of the bases in the order A, C, G, T and the count of forward and reverse matches. c4p takes about 3 seconds.

cmpl *input_file*

cmpl outputs the reverse complement of *input_file *to standard out. Only the uppercase characters A, C, G and T are complemented; all other characters are passed through unchanged, except for a limited set of wild-card characters (see Regular Expressions below) and > which is converted to <. If no *input_file *is specified cmpl waits for input from the keyboard and continues to take input from the keyboard until it is ended with <Ctrl>-d.

A complete multi-line sequence can be reverse complemented by using cmpl followed by the Linux command tac, e.g.

cmpl *input_file *| tac >*output_file*

Note that any FASTA header will be mangled and will come at the end of its sequence. cmpl is intended to operate on files with a line length of 60 characters cmpl takes about 0.27 seconds to complement 4100 bases in lines of 60 characters; the time increases linearly with number of lines and as the square of the line length.

Where any of these tools expects input from standard in (the keyboard) or sends output to standard out (the monitor) the input or output can be redirected to or from a file with < and > respectively, e.g.

fr <*input_file output_file*

cmpl *input_file *>*output_file*

### Regular expressions

A very limited sub-set of regular expressions has been implemented to help with searching for partial matches and polymorphisms.

Wildcard.

The wildcard character. (period) can be used to substitute for any base. Two or more may appear in succession, e.g. AACT.TCC and AACT...TCC are both valid.

Multiplier *

The * character returns zero or more of the immediately preceding character, e.g. A* matches zero, one or more A, so AA* matches one or more A.

Alternatives [*mn*]

[*mn*] matches one of the characters contained in the square brackets, e.g. [AT] matches either A or T.

One or more of these may appear in any *match_string *or *search_string*, but they may not be combined in the same position; i.e. ACT [AT]CCG*A..A is valid but ACT [AT]*C is not as [*mn*] and * cannot be combined.

## Results and Discussion

### Additional information available from the 454 dataset

Although the 454 sequencing method produces characteristic errors as noted below, it also contains additional information that is not available from conventional sequencing. When the material to be sequenced is clonal, the result of the 454 method is essentially equivalent to Sanger sequencing. However when the original material is not clonal, the 454 sequencing will effectively sample the sequence many times at each position (approximately 185 times in our sample), permitting any polymorphisms to be investigated.

### Single-base polymorphisms

If an alternate sequence exists in the sample being sequenced at the level of 10% or so, Sanger sequencing will always return the majority sequence as the minority sequence signal will be masked by the majority signal. The 454 system however effectively samples and clones a large number of individual sequences and returns a read for each. During assembly by the company the majority sequence will be selected, but the minority base can be discovered from the original reads so long as there is some indication of where to look, together with an estimate of the frequency.

For example in our data, we noted some possible variability in the following sequence at the underlined locations:

GGTAT**A**TTAGGTGTG**C**TGTGAACAGGATTG**C**TAACAAAA**G**TAACAC**C**CCCAT

Using the tool fr2 with the *match_string*:

GGTAT.TTAGGTGTG.TGTGAACAGGATTG.TAACAAAA.TAACAC.CCCAT

produces the output shown in Supplementary Table 3 [see Additional file 3]. At the foot of the table are four instances, out of 60 matches, of the alternate form with all five of the polymorphic bases consistently changed. Whilst the error rate in individual reads from the 454 sequencing is quite high, to get five different bases all changed consistently strongly indicates the presence of a minority sequence with a frequency of about 6%.

### Assembling highly repeated regions

A number of tools are available to help with the assembly of repeat regions [18-22] but these are not specifically designed for the short reads from pyrophosphate sequencing. Pyrophosphate sequence data presents a particular challenge to assembling repeats because of the short read lengths and higher error rates. However, it also provides two advantages over Sanger sequencing:

1. The fragments generated by the shearing are incorporated at random onto the beads, avoiding the difficulties produced by sequences being under-represented because they are difficult to clone.

2. The large number of reads means that each sequence is read many times.

Repeats longer than about 100 bases become increasingly difficult to resolve, and in particular, exact repeats can only be assembled if there are reads longer than the repeat. However if the repeats are not exact the 454 data will provide information that can lead to at least partial assembly, and will indicate the number of copies even for exact repeats.

The commonest problem with assembling repeat regions is the collapsing of repeats. If the repeats are 100% identical this is difficult to avoid, but when the repeats differ by just one or two bases there is a risk that the assembler will treat the changed base as an error and misalign the sequences. As the tool build accepts only exact matches it can be used to force an assembly through a region of high repeat, so long as the individual base changes occur at least once in 60 bases. Where stretches of sequence occur that are identical for more than 60 bases build will stop at the split point, indicating the two or more possible variants. Each possibility can then be followed independently until they converge once more.

**Figure 2:**
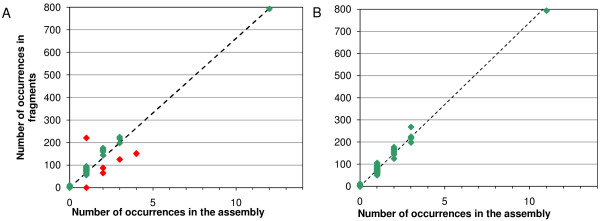
**Number of reads for repeat elements**. a) For an intermediate stage of finishing. b) After completion of finishing; the dashed line is the computed trend line with gradient 74.02 and intercept zero.

The power of the 454 data to identify misreads and to indicate the number of copies of a given repeat sequence can be illustrated from the GpSGHV data. The virus sequence contains a region of repeats between nucleotide position 178 000 and position 183 000. The repeats consist of alternating 27 base elements designated <p> and <q>, the individual variants of the repeats being indicated by a sequence number, e.g. <p4>. Supplementary Table 4 [see Additional file 3] shows the frequency of reads in the 454 data set determined using count for known repeat sequences and a sample of sequences created by changing a single base in a known repeat, together with the known number of occurrences in the sequence. The erroneous sequences occur in the fragments with very low or zero frequency, making it easy to distinguish real sequences from read errors. Fig. 2a shows the number of occurrences of repeat elements against the presumed number of instances of each element in the sequence of GpSGHV before the sequence was finished. Points lying off the trend line, marked in red, indicate errors in the assembly. Points above the line represent sequences that have been collapsed, and points below the line ones that have been incorrectly duplicated. The point lying on the X-axis indicates a sequence where the assembler had incorrectly inserted a base. After correction of the sequence and the erroneous base, the points all lie near the trend line (Fig. 2b). re can then be used to recheck the repeat region, which will indicate the presence and approximate number of repeats in the sequence and any erroneous bases remaining (see Fig. 1).

### Issues with the pyrophosphate sequence results

Pyrophosphate sequencing suffers from two main errors, incomplete chain extension and carry forward [1,5]. In our experience carry forward is not a significant problem but incomplete chain extension is, where during a given flow sequence the chain extension is not completed for a proportion of the chains in each well. The remaining bases of the homopolymer will then be copied in the next flow cycle, resulting in the extension being one flow behind the majority. In most cases this is ignored by the reading system, but two different errors can occur because of this. Firstly, the incomplete extension produces a weaker signal than usual, which the system will interpret as a shorter homopolymer when the signal falls below the predefined threshold leading to variability in the apparent length of a given homopolymer. Secondly, when another long homopolymer occurs further along the molecule, the signal generated by the copying of this homopolymer one flow behind the majority can lead to a signal large enough for the system to miscall an additional base.

### Homopolymer length

**Figure 3:**
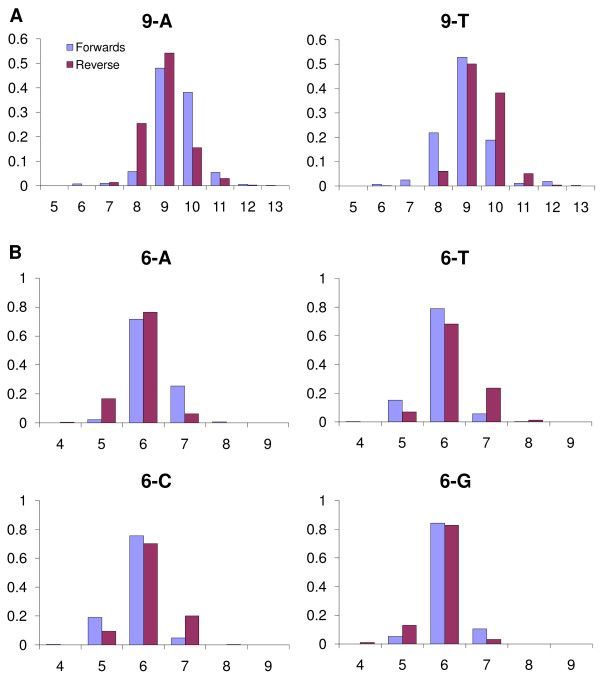
**Homopolymer lengths**. a) Reported homopolymer lengths in the 454 data set for all 9-mers of A and T in the GpSGHV. b) Reported homopolymer lengths in the 454 data set for all 6-mers of C and G and a random selection of 6-mers of A and T in the GpSGHV.

The GpSGHV has a C/G ratio of 28%. As a result homopolymers of C or G are limited to 7-mers but both A and T homopolymers up to 11-mer are present. There are two few of the longest homopolymers to produce a meaningful analysis but there are fifteen 9-mers of A and thirteen 9-mers of T. Using count the number of forward and reverse copies of each 9-mer with ten bases each side was counted and averaged (Fig. 3a). The reading of poly-T (and reverse reading of poly-A) is almost symmetric about the true value with only a slight tendency to under-read, but there is a consistent trend towards over-reading poly-A (and reverse poly-T).

Table 1 gives the length of reads for the sequence

TTTT*AAAA*TTAAAAAAAAATCCTCCGAGT

**Table 1:** Homopolymer read lengths

Homopolymer	Read direction		
Length	Forward	Reverse	Total
8	2	6	8
9	10	22	32
10	12	20	32
11	1	6	7

Read lengths in the 454 data for the GpSGHV sequence starting at position 86587, TTTT*AAAA*TTAAAAAAAAATCCTCCGAGT

at position 86587 in the GpSGHV. The * is the regular expression multiplier (see above) so that reads where the initial 4-T and 4-A are read as three or more are included to increase the sample size. The 9-A appears in the fragments from 8-mer to 11-mer, and taking only the total number of fragments there is a tie between 9-mer and 10-mer. However knowing from above the consistent tendency to over-read A, more weight can be given to the reverse reads, indicating a 9-mer. This was confirmed by directed Sanger sequencing.

Fig. 3b shows the fragment homopolymer length for all 6-mers of C and G, the longest homopolymers of C and G with enough occurrences to analyse, and of a random sample of ten 6-mers of A and T. The tendency to over call A and under call T is still visible, if not as strong, but there is also a tendency to over call G and under call C. However, at length 6 this never seems to lead to a misassembly by Newbler.

It is not possible to tell from our data if this is a systematic reading error or a result of the post reading corrections applied by 454 to correct some of the errors.

### Additional base called

The second common error is the addition of a spurious base in the flow cycle following a long homopolymer. This occurs when a sufficient number of chains have become out of step and a long homopolymer is read. The reading of the out of step long homopolymer can produce a signal large enough to be read as an additional base. We have noted from our data that incomplete chain extension is much more common than carry forward, so the misread base normally occurs after the long homopolymer and not before it. This asymmetry means that additional bases can be simply identified by comparing the forward and reverse reads. The addition of bases is much more common in our data with long A homopolymers than with the other bases.

Table 2 shows three examples of this. In each case the misread is given first followed by the corrected read. The tool count returns the number of matches in the forward and reverse directions, and in both cases the number of fragments matching the misread in the complement direction is very low (equivalent to a base incorrectly inserted before the homopolymer), but when the sequence is corrected the number matching in the forward and complement direction is almost equal.

**Table 2:** Bases inserted due to incomplete extension.

	Forward	Reverse
TTAAAATAAAAAAAAAG**A**CGCAT	17	0
TTAAAATAAAAAAAAAGCGCAT	8	14
TAAAAAAATAAAAAACT**A**GGTTT	18	2
TAAAAAAATAAAAAACTGGTTT	11	13
TATTGTAAATAAAAAAAAAATA**A**TACA	18	1
TATTGTAAATAAAAAAAAAATATACA	6	15

Paired output from count with the incorrect sequence followed by the corrected sequence. In each case, incomplete extension has resulted in the insertion of a spurious base in the following flow cycle in the forward direction, but no or very few reads in the reverse sense. When the spurious base is removed, the numbers are more nearly matched in the forward and reverse directions. In the third example, this effect caused a single A to be read as AA.

## Conclusion

454 sequencing provides a very large set of reads with consequently a very high over-read rate. The individual reads however are short, and as a result it is difficult to assemble them with conventional assembly programs. The company provides both the individual reads and the assembly, but depending on the complexity of the sequence the assembly may be in many separate contigs. The set of individual reads contains information on homopolymer length, base insertions and repeats not always captured by the assembled contigs, but this information is difficult to access due to the very large size of the dataset which is difficult to handle.

There is a need for an assembler specifically designed for the assembly of such reads that will also allow a detailed examination of the read database to extract this additional information. In order to facilitate access to this additional information in the absence of a suitable assembler we have developed a set of simple tools running under GNU Linux. Using these tools, we have been able to resolve issues of homopolymer length, incorrectly inserted reads and potential polymorphisms, to check draft assemblies against the reads for consistency and conduct limited *de novo *assembly from the reads for contig joining. We have also been able to check in detail the occurrence of specific variants of repeats, thereby correcting misassembled repeats and demonstrating that a block of repeats had been wrongly duplicated by estimating the number of occurrences of individual repeat variants in the complete sequence.

## Availability and requirements

The supplementary tables, source code, executables and instructions are available from our web page[17].

Requirements:

• Intel-compatible CPU (Pentium),

• 256 MB of RAM, 384 MB recommended to allow the Open Office programs to be used to produce files compatible with Microsoft Office,

• USB flash memory drive or external hard disk with a FAT32 formatted partition or FAT32 formatted partition on the internal hard drive,

• bootable CD-ROM drive, or a boot floppy and standard CD-ROM (IDE/ATAPI or SCSI),

• standard SVGA-compatible graphics card,

• serial or PS/2 standard mouse or IMPS/2-compatible USB-mouse,

• Knoppix 5.1 CD or DVD.

## Copyright, Trademarks, Licensing and registration requirements

This software is released under the GNU General Public License version 2.

## Competing interests

The authors declare that they have no competing interests.

## Authors' contributions

NJP wrote the code and script files. AGP developed the requirements and drafted the manuscript. Both authors collected the data presented, read and approved the manuscript.

## Supplementary Material

Additional file 1 Installation instructions. Description of how to launch and configure the Knoppix system, how to set up a home directory in the FAT32 file system and how to unpack the tgz file containing the tools. Click here for file

Additional file 2 Tool files. Compressed file containing the directory structure, the executable and script files, sample files, source code and additional documentation. Click here for file

Additional file 3 Supplementary tables. Supplementary tables containing sample output from several of the tools. Click here for file
