# Supplementary material for: Simple tools for assembling and searching high-density picolitre pyrophosphate sequence data
Source: Source Code Biol Med. 2008 Apr 18;3:5. doi: 10.1186/1751-0473-3-5 (PMC2374781; doi:10.1186/1751-0473-3-5)
Supplement: Additional file 1 — Installation instructions. Description of how to launch and configure the Knoppix system, how to set up a home directory in the FAT32 file system and how to unpack the tgz file containing the tools. [file 1751-0473-3-5-S1.pdf]

Setting up and running the pyrophosphate tools under the Knoppix GNU Linux system

N.B. These instructions are for using the Knoppix Live CD or DVD. If you want to use the tools on another Debian Linux system you will need to do your own configuration. In particular do not use the `setup` script as this may delete your `.bashrc` configuration file.

CONTENTS

1. Requirements
2. About Knoppix
3. Storage requirements
4. Running Knoppix and setting up the file structure
5. Unpacking the tools
6. Running Knoppix after the initial setup
7. Some comments on Linux, commands and utilities
8. vim
9. Sample files

1. Requirements:

1 Intel-compatible CPU (Pentium or later),

2 256 MB of RAM, 384 MB recommended to allow the Open Office programs to be used to produce files compatible with Microsoft Office,

3 USB flash memory drive or external hard disk with a FAT32 formatted partition or FAT32 formatted partition on the internal hard drive,

4 Bootable CD-ROM drive, or a boot floppy and standard CD-ROM (IDE/ATAPI or SCSI),

5 Standard SVGA-compatible graphics card,

6 Serial or PS/2 standard mouse or IMPS/2-compatible USB-mouse,

7 Knoppix CD or DVD (5.1).

8 The compressed archive file pyrophosphate_tools.tgz from the Source Code for Biology and Medicine web site http://www.scfbm.org/home/

9 This file pyrophosphate_readme.txt

2. About Knoppix

Knoppix is a "live" CD/DVD version of GNU Linux. This means that Linux runs directly from the CD or DVD without using your computer's hard disk, so that it does not interfere in any way with an existing Windows installation. As Knoppix runs from the CD or DVD the disk needs to remain in the drive all the time.

For details on how to download the Knoppix disk images and burn them to CD or DVD, or to obtain them by mail see http://www.knopper.net/knoppix/index-en.html. If you do not have a bootable CD or DVD drive you will need to boot from a floppy disk, see the Knoppix web pages for instructions. The web pages also contain much more information about Knoppix and what you can do to configure it. A tutorial for Knoppix is available at http://www.elearnit.de/knoppix/docs/tutorial/english/index.html

3. Storage requirements

Until recently Linux could not read the standard disk format for Windows versions after 2000 (NTFS). Whilst it is now possible to read and write NTFS partitions in Linux it is better to use a separate storage system with the FAT32 format as this can be read by both Linux and Windows. Ideal for this is a flash memory stick of 1 or 2 GB as these are FAT32 formatted. Alternatively an external USB hard drive can be used, but you will have to format the whole disk, or a partition on the disk, as FAT32. To format the disk as FAT32, or to create a new partition and format it, please see the Windows help. The final choice is to format a partition on the internal hard disk as FAT32. However we are proposing the use of Knoppix so as not to disturb the Windows installation on the computer so this will not de discussed further here.

Using Windows, create a folder on the memory stick or external hard drive to store all the pyrophosphate data and the tools. In Linux, Windows folders are known as directories or sub-directories. For ease of use in Linux use all lower case letters and avoid spaces; if you want to separate words use - or _. It is probably best to have the folder (directory) at the top level in the FAT32 partition. If its name is unique after a small number of initial characters, then that helps when using the shell's automatic filename completion (by typing the initial characters and then a tab character). For simplicity this will be referred to as "data" in these notes. When you see "data" in the notes replace it with your own name. Copy the `pyrophosphate_tools.tgz` file to this directory (folder). Also copy all the files from 454 with the extension `.fna` to this directory.

4. Running Knoppix and setting up the file structure

To run Knoppix put the CD or DVD into the disk drive and reboot the computer. Knoppix should start automatically. If the computer boots back into Windows you will need to change the boot priority. Reboot the computer again but this time as it starts to reboot watch the bottom line of the display. As the BIOS loads (the first step in booting the computer) a row of options will appear on the bottom line. Check which key will take you to either Options or Boot Options - it might be Delete, F1, F8 or another function key. Press the appropriate key before the options go from the screen. The options are not on the screen for long so you may need to reboot again to give yourself time to read and select an option. If you do not see the boot options consult the instructions with your computer.

Once you are in Boot Options (or if you are in Options, select Boot Options first) you must select your CD or DVD drive and set it to first in the priority list. Make sure that the hard disk still appears on the list as second or subsequent priority so that you can boot back into Windows. Save the changes and exit the Options menus. Knoppix should now boot. First there is a welcome screen, press Enter or wait a few seconds and it will go. Knoppix then examines your computer and selects the necessary drivers. This will take a minute or two. The X Window desktop appears next, and finally a window opens with information about Knoppix. This window can be closed or minimized. If Knoppix fails to boot on your computer check the help information on the Knoppix web site.

The desktop looks a bit like a Windows desktop. There are icons on the desktop for the disk drives and other locations, and at the bottom left a row of icons. The first accesses various programs, including Log Off which takes you to the shut down functions; to shut down allow time for the operating system to close down all the running programs and unmount the file systems then remove the CD or DVD from the drive when it opens automatically. The sixth icon from the left is the terminal session icon and is the main one used by these tools. Next to that are a web browser and Open Office, a suite of programs including word processing and spreadsheet.

If you now insert your memory stick or USB drive, after a few moments a new icon will appear on the desk top; if the drive has more than one partition one icon will appear for each partition. Linux creates a single file system of all available storage, instead of allocating drive letters to individual drives as in Windows. In order to access the memory stick or USB drive it has to be “mounted” onto the file system and made writable. If you place the mouse on the icon a hover box appears with information about the drive. The last but one line is labelled "Mount Point:" - take a note of the mount point listed. It will be something like /media/sdb or /media/sdc2, depending on the number of USB ports on the computer, whether the internal hard drive is serial or parallel and whether the drive has more than one partition. Now right click the icon and select mount. Right click the icon again and select "Change read/write mode"; a confirmation window will appear asking if you really want to change it, select yes. The memory stick or USB drive is now mounted and accessible.

Next open a terminal session (sixth icon from the left at the bottom of the screen). The window can be resized with the mouse by dragging a border and if the font size is too large it can be changed through the Settings - Font menu. At the top of the new window that opens you will see the command prompt, which is `knoppix@Knoppix:~$`. The ~ indicates that this is currently the “home” directory. It now waits for you to type something.

Change to the new directory in the memory stick by typing:

```
cd /media/sdb/data
```

and then press Enter (end all commands with Enter). Replace the "/media/sdb/" with the mount point you noted earlier and "data" with the name you used for your folder. Note that Linux commands are lower case and folder and file names are case sensitive. The command prompt changes to `knoppix@Knoppix:/media/sdb/data$` with your drive and directory details.

If you now type:

```
ls -l
```

you should see a listing of *pyrophosphate_tools.tgz* and any other files you copied to this directory.

5. Unpacking the tools

To unpack the tools type:

```
tar xvzf pyrophosphate_tools.tgz
```

This will unpack the files and create the necessary directories. You only need to do this once. Now type:

```
setup
```

(N.B. Only use this `setup` script with the Knoppix system as described here. The results on any other Linux system are unpredictable and we will not accept any responsibility for any damage caused.)

This will change your home directory to the current directory so that you do not have to keep entering */media/sdb/data* all the time and will set the $PATH variable to point to the new directory containing the tools so that they can be run. The prompt changes back to `knoppix@Knoppix:~$` to show that the home directory has been set to the current location.

Repeat the `ls` command but this time with different options:

```
ls -al
```

and now you should see several subdirectories; `bin, csrc, Docs` and `database` as well as the files *setup* and *.bashrc* and some sample files. The additional option in `-al` causes files starting with . to be displayed as well when they would normally be hidden. These . files are equivalent to Windows system files.

Finally convert the *.fna* file to the format for these tools. Type:

```
fna_to_fnb <file_name.fna >database/file_name.fnb
```

where *file_name.fna* is the name of the file from 454 that you copied into the directory on the memory stick or USB external hard disk in 3 above (use `ls` to check the name if you need to). This will create the file *file_name.fnb* in the subdirectory *database*. If you have more than one *.fna* file from 454 you will need to use different names for the *.fnb* files to avoid them being overwritten.

A sample database of 454 fragments called *sample.fnb* is provided in the subdirectory *database*. This can be used to try out the various tools. The tools look for the fragment database file specified by the variable `FRAGMENT_DB` in the directory specified by the variable `FRAG_DB_DIR` (by convention variable names are in upper case). These variables are set in the *.bashrc* file in your home directory to *~/database* and *sample.fnb* respectively. To change to a different directory type at the terminal session prompt:

```
FRAG_DB_DIR=~/directory_name; export FRAG_DB_DIR
```

and to change the fragment file name type:

```
FRAGMENT_DB=file_name.fnb; export FRAGMENT_DB
```

To permanently change the start-up default directory and file edit the *.bashrc* file in your home directory using `vim` (see below).

6. Running Knoppix after the initial setup

To run Knoppix again (on any PC) do the following:

1. Boot the computer with the Knoppix disk in.
2. Insert the memory stick or USB drive.
3. Mouse over the icon and check the mount point.
4. Right click the icon and mount the drive.
5. Right click the icon and make the drive read/write.
6. Open a terminal window (sixth icon from the left).
7. Type `cd /media/sdb/data` to change to your directory (where `/media/sdb` is replaced with the mount point noted in 3)
8. Type `setup`

You are now ready to work! The setup command changes the working directory to the current directory so if you open another terminal window it will automatically be in your home directory. You can also open a second terminal instance by clicking the icon at the bottom left of the terminal window.

7. Some comments on Linux, commands and utilities

If you are unfamiliar with Linux it may be a little strange to begin with. In these brief notes we cannot hope to teach you everything, but a few comments may be helpful to get you started. A search of the web for "Linux commands" will produce many web sites to help if you want more information.

Single left click to select
Right click for shortcut menu
Left click and drag to highlight and copy to clip board
Centre click (usually clicking the scroll wheel) to paste, or right click and select paste
When a command produces output to the screen (standard out) this can be redirected to a file with > e.g.

```
count AATCTCCAGGCT >1.txt
```

will create a text file called `1.txt` with the output from `count`. If `1.txt` already exists it is overwritten.

When a command expects input from the keyboard (standard in) this can be redirected from a file with < e.g.

```
count <2.txt
```

will count the occurrences of the string contained in 2.txt in the fragment database.

Some terminal window commands:

`cp` *`source_file destination_file`*	copies a file e.g. `cp` *`1.txt database/temp`* creates a new copy of *`1.txt`* called *`temp`* in the directory *`database`*
`cp` *`./* destination_directory`*	copies everything in the current directory to the destination directory
`rm` *`file_name`*	deletes a file (or directory)
`mv` *`source_file destination_file`*	changes the name of *`source_file`* to *`destination_file`*
`mv source_file directory`	moves the *`source_file`* to *`directory`*
`cd` *`directory_path`*	changes to the specified directory e.g. `cd` *`database`* changes to the *`database`* directory
`cd`	changes back to the current home directory
`mkdir` *`new_name`*	creates a new directory
`cat` *`text_file`*	lists a file to the screen
`ls` *`directory_path`*	lists files in directory (current directory if no path given)
`ls -l` *`directory_path`*	lists with more information
`ls -al` *`directory_path`*	list files including files starting with .
`unix2dos` *`file_name`*	converts a text file into the format that Windows reads
`dos2unix` *`file_name`*	converts from Windows to Unix (Linux) format
`man command_name`	gives the manual page for `command_name` with all the details you ever wanted, e.g. `man ls` gives the manual page for `ls`

8. vim

`vim` is a sophisticated command line text editor that is very useful for looking at the output files from the tools (or where the output has been redirected with > to a file). We only include a very brief description of `vim`; for a full description see `http://vimdoc.sourceforge.net/vimfaq.html`

Start `vim` in a terminal window with:

```
vi file_name
```

If *`file_name`* already exists the contents will appear in the window with a status line at the bottom showing the cursor position; if it does not exist a new file will be created and the window will show just a series of ~ indicating empty lines. `vim` handles files in both Unix (Linux) and Windows formats with Windows files indicated by `[DOS]` on the status line.

The standard cursor keys move you around the file and the `<del>` key deletes. `i` enters insert mode which will remain on until `<esc>` turns it off. It is important to remember this when trying to enter any of the commands listed below as they only work when insert mode if off - otherwise they are just entered into the file as new text. Sections can be copied by clicking with the mouse and dragging (or double-click on a "word" - any string of characters surrounded by white space) which you can paste into another terminal window (centre mouse button) for some other operation.

`vim` can handle very long lines, so long as the terminal window is not set too small. If you adjust the window to at least 100 characters wide it can handle single lines with 200,000 characters. This is very useful for examining assembled sequences. Starting with the fasta file of the sequence remove the header, then using the command listed below remove all line breaks. The complete sequence now appears as a single line. Using `nn|` you can move to any position in the sequence, or search for

a given string with / and the search string. The location of the found string is then shown at the bottom of the screen on the status line. Note that with very long lines (hundreds of thousands of bases) the cursor position shown at the bottom of the screen, which gives you the position in the sequence, will be wrong if the terminal window is too narrow – there is some interaction between line length and window width but we have not determined the limits of this. If you are unsure note the position reported for the cursor, then increase the width of the window by dragging a border with the mouse; if the reported position remains the same there is not a problem.

If a second copy of the fasta file is complemented and the lines reversed, when added as a second line in the file both forward and reverse complement matches can be searched simultaneously.

If `vim` is invoked with

```
view file_name
```

the file is opened in read-only mode. Changes can be made, but they cannot be saved unless saving is forced with `:w!`

A GUI version of `vim` called `gvim` is available in Knoppix from the start menu under Editors. `gvim` is also available in versions for other operating systems including Windows.

<u>Selected vim commands</u>

Commands that are executed when you press <Enter>:

`:set ic`	ignore case
`/`*string*	search forward
`?`*string*	search backwards
`:s/`*string1*`/`*string2*	replace *string1* with *string2*
`:w`	save file
`:w!`	force save of a read-only file
`:q`	quit
`:q!`	force quit without saving changes
`:e!`	discard changes - re-edit original file

Commands that are executed immediately:

`i`	enter insert mode (until `<esc>` ends it)
`u`	undo
`nnnG`	go to line `nnn`
`G`	go to last line
`¦`	beginning of line
`nn¦`	go to column `nn`
`n`	repeat search
`N`	repeat search other direction
`r`*x*	replace one character with `x`
`R`	replace until `<esc>`
`nnx`	delete `nn` characters from cursor
`o`	(lower case o) insert line below current one and enter insert mode (end with `<Esc>`)
`O`	(upper case o) insert line above current one and enter insert mode (end with `<Esc>`)
`D`	delete from cursor to end of line
`dd`	delete line containing cursor
nn`dd`	delete `nn` lines from line containing cursor

In search and match strings various special characters can be used (regular expressions). Some of them are:

`^`	start of line
`$`	end of line eol
`\<`	beginning of word
`\>`	end of word

`[7-9]`	match anything in range
`[^7-9]`	match all except
`[xyz]`	match several
`\n`	new line character
`.`	wildcard single character
*a**	any number of *a*

The following sample commands may be useful:

`:%s/\n` remove all line breaks

`:%s/^.*`*sequence*`/`*sequence*
delete the start of line up to *sequence* (last instance)

`:%s/`*sequence*`/# :%s/^.*#/`*sequence*
delete the start of line up to *sequence* (first instance)

`:%s/`*sequence1*`/`*sequence2*`/g`
replace every instance of *sequence1* with *sequence2* throughout the whole file

Sample files

In addition to the sample database file *sample.fnb* mentioned above, the corresponding section of the SGHV (16-20 kb) sequence is provided in the file *sample_sequence* (complete sequence in a single line with the reverse complement in a second line), *sample_sequence.fas* in standard fasta format and a number of primer files. Using these files various features of the tools may be tried.

primer1 contains the first 65 bases of this sequence. The command:

```
build primer1 build_1
```

will assemble in the file *build_1* the sequence using the sample fragment database. The assembly stops at base 502 due to an ambiguity with 9 C and 6 A. The ambiguity is quickly resolved by using c4 with the last 60 bases of *build_1* as the *match_string*, showing that most of the As come from the forward read direction with only one in the reverse direction. This is a typical incorrectly inserted base due to an incomplete extension error.

primer2 takes the last 64 bases of *build_1* with C appended allowing one to continue the build. With *primer2* the build stops after another 654 bases, this time due to the tendency in our 454 dataset to over-read A – the 7-A homopolymer appears 8 times as an 8-mer and 13 times as a 7-mer. Again examination of the fragments, this time using fr2 with the last 30 bases of *build_2* shows that the 8-mer appears only once in the reverse reads (where A is read as T). c4 would lead to the same conclusion.

primer3 again contains the last 64 bases of *build_2* with T appended. Using this primer 1143 bases are assembled until assembly stops due to too few fragments.

primer4 is located shortly after the stop point from *primer3*. Using `build` with this primer stops after 1390 bases with 83 C, 71 T and 3 A. The 3 A can be ignored, but the near equality in the number of C and T, and no significant bias in the read direction using c4, indicates the presence of a repeat sequence with an exact match at least 60 bases long. In this case the repeat lies outside the sample provided, at 22092. The difference between the forward and reverse count for the T may indicate that this position should be investigated further but is probably simply due to the tendency to over-read A.

Once a putative sequence has been assembled it can be checked against the fragment database for consistency. The sample sequence is provided in the file *sample_sequence.fas*. Using:

```
re sample_sequence.fas sample_re
```

Viewing the output file with `vi` many of the above issues can be seen. Abrupt changes in the number of matches indicate repeat sequences, and areas with zero match indicate potential errors in the putative sequence. The abrupt drop to zero at line 60 of *`sample_re`* shows the presence of an error that can be confirmed by comparison with *`build_1`*. However the more gradual decline seen around line 2113 can be due to the variability in reading homopolymers or a shortage of fragments in the database due to other unknown factors. The output from `re` can be conveniently imported into a spreadsheet to graph the number of matching fragments to give a pictorial representation of the number matching the putative sequence as in Figure 1 of the main paper.
