# Supplementary material for: Simple tools for assembling and searching high-density picolitre pyrophosphate sequence data
Source: Source Code Biol Med. 2008 Apr 18;3:5. doi: 10.1186/1751-0473-3-5 (PMC2374781; doi:10.1186/1751-0473-3-5)
Supplement: Additional file 2 — Tool files. Compressed file containing the directory structure, the executable and script files, sample files, source code and additional documentation. [file 1751-0473-3-5-S2.tgz › Docs/vimweb.pdf]

Vim documentation: vim_faq

This Vim FAQ is created from the questions and answers posted to the vim@vim.org user mailing list and the comp.editors newsgroup. There are several ways to solve a problem in Vim. This FAQ gives one of those several possibilities. You can explore the other ways using the information and links given in this FAQ. The credit for the answers in this FAQ goes to Peppe, Benji, Charles Campbell and numerous others.

INDEX

SECTION 1 - GENERAL INFORMATION
1.1. What is Vim?
1.2. Who wrote Vim?
1.3. Is Vim compatible with Vi?
1.4. What are some of the improvements of Vim over Vi?
1.5. Is Vim free?

SECTION 2 - RESOURCES
2.1. Where can I learn more about Vim?
2.2. Is there a mailing list available?
2.3. Is there an archive available for the Vim mailing lists?
2.4. Where can I get the Vim user manual in HTML/PDF/PS format?
2.5. I have a "xyz" (some) problem with Vim. How do I determine it is a problem with my setup or with Vim?
2.6. Where can I report bugs?
2.7. Where can the FAQ be found?
2.8. What if I don't find an answer in this FAQ?
2.9. I have a patch for implementing a Vim feature. Where do I send the patch?
2.10. I have a Vim tip or developed a new Vim syntax/indent/filetype/compiler plugin or developed a new script or a colorscheme. Is there a public website where I can upload this?

SECTION 3 - AVAILABILITY
3.1. What is the latest version of Vim?
3.2. Where can I find the latest version of Vim?
3.3. What platforms does it run on?
3.4. Where can I download the latest version of the Vim runtime files?

SECTION 4 - HELP
4.1. How do I use the help files?
4.2. How do I search for a keyword in the Vim help files?
4.3. I am getting an error message E123, what did I do wrong?
4.4. Where can I read about the various modes in Vim?
4.5. How do I generate the Vim help tags file after adding a new Vim help file?
4.6. Can I use compressed versions of the help files?

SECTION 5 - EDITING A FILE
5.1. How do I load a file in Vim for editing?
5.2. How do I save the current file in another name (save as) and edit a new file?

5.3. How do I change the current directory to the directory of the current file?
5.4. How do I write a file without the line feed (EOL) at the end of the file?
5.5. How do I configure Vim to open a file at the last edited location?
5.6. When editing a file in Vim, which is being changed by an external application, Vim opens a warning window (like the confirm dialog) each time a change is detected. How do I disable this warning?
5.7. How do I edit a file whose name is under the cursor?
5.8. How do I reload/re-edit the current file?
5.9. How do I autosave a file periodically?
5.10. How do I open a file in read-only mode?
5.11. How do I open a file for editing without saving the modifications to the current file?

SECTION 6 - EDITING MULTIPLE FILES
6.1. How do I open multiple files at once from within Vim?
6.2. How do I switch between multiple files/buffers in Vim?
6.3. How do I open several files in Vim, with each file in a separate window?
6.4. How do I configure Vim to autoload several files at once similar to "work-sets" or "projects"?
6.5. Is it possible to open multiple top level windows in a single instance of Vim similar to Nedit or emacs?
6.6. How do I browse/explore directories from within Vim?
6.7. How do I edit files over a network using ftp/scp/rcp/http?

SECTION 7 - BACKUP
7.1. When I edit and save files, Vim creates a file with the same name as the original file and a "~" character at the end. How do I stop Vim from creating this file (or) How do I disable the Vim backup file feature?
7.2. How do I configure Vim to store all the backup files in a particular directory?
7.3. When I save a file with Vim, the file permissions are changed. How do I configure Vim to save a file without changing the file permissions?

SECTION 8 - BUFFERS
8.1. I have made some modifications to a buffer. How do I edit another buffer without saving the modified buffer and also without losing the modifications?
8.2. How do I configure Vim to auto-save a modified buffer when switching to another buffer?
8.3. How do I replace the buffer in the current window with a blank buffer?
8.4. Is there a keyboard shortcut to load a buffer by the buffer number?
8.5. How do I open all the current buffers in separate windows?
8.6. How do I close (delete) a buffer without exiting Vim?
8.7. I have several buffers opened with ":e filename". How do I close one of the buffers without exiting Vim?
8.8. When I use the command ":%bd" to delete all the buffers, not all the buffers are deleted. Why?
8.9. How do I display the buffer number of the current buffer/file?
8.10. How do I delete a buffer without closing the window in which the buffer is displayed?
8.11. How do I map the tab key to cycle through and open all the

buffers?

SECTION 9 - WINDOWS
- 9.1. What is the difference between a Vim window and a buffer?
- 9.2. How do I increase the width of a Vim window?
- 9.3. How do I zoom into or out of a window?
- 9.4. How do I execute an ex command on all the open buffers or open windows or all the files in the argument list?

SECTION 10 - MOTION
- 10.1. How do I jump to the beginning (first line) or end (last line) of a file?
- 10.2. In insert mode, when I press the <Esc> key to go to command mode, the cursor moves one character to the left (except when the cursor is on the first character of the line). Is it possible to change this behavior to keep the cursor at the same column?
- 10.3. How do I configure Vim to maintain the horizontal cursor position when scrolling with the <Page Up>, <Page Down>, etc keys?
- 10.4. Some lines in a file are more than the screen width and they are all wrapped. When I use the j, k keys to move from one line to the next, the cursor is moved to the next line in the file instead of the next line on the screen. How do I move from one screen line to the next?
- 10.5. What is the definition of a sentence, paragraph and section in Vim?
- 10.6. How do I jump to beginning or end of a sentence, paragraph or a section?
- 10.7. I have lines in a file that extends beyond the right extent of the screen. How do I move the Vim view to the right to see the text off the screen?
- 10.8. How do I scroll two or more buffers simultaneously?
- 10.9. When I use my arrow keys, Vim changes modes, inserts weird characters in my document but doesn't move the cursor properly. What's going on?
- 10.10. How do I configure Vim to move the cursor to the end of the previous line, when the left arrow key is pressed and the cursor is currently at the beginning of a line?
- 10.11. How do I configure Vim to stay only in insert mode (modeless editing)?
- 10.12. How do I display some context lines when scrolling text?
- 10.13. How do I go back to previous cursor locations?

SECTION 11 - SEARCHING TEXT
- 11.1. After I searched for a text with a pattern, all the matched text stays highlighted. How do I turn off the highlighting temporarily/permanently?
- 11.2. How do I enter a carriage return character in a search pattern?
- 11.3. How do I search for the character ^M?
- 11.4. How can I search/replace characters that display as '~R', '~S', etc.?
- 11.5. How do I highlight all the non-printable characters in a file?
- 11.6. How do I search for whole words in a file?
- 11.7. How do I search for the current word under the cursor?
- 11.8. How do I search for a word without regard to the case (uppercase or lowercase)?
- 11.9. How do I search for words that occur twice consecutively?
- 11.10. How do I count the number of times a particular word occurs in a buffer?

11.11. How do I place the cursor at the end of the matched word when searching for a pattern?
11.12. How do I search for an empty line?
11.13. How do I search for a line containing only a single character?
11.14. How do I search and replace a string in multiple files?
11.15. I am using the ":s" substitute command in a mapping. When a search for a pattern fails, the map terminates. I would like the map to continue processing the next command, even if the substitute command fails. How do I do this?
11.16. How do I search for the n-th occurrence of a character in a line?
11.17. How do I replace a tab (or any other character) with a hard return (newline) character?
11.18. How do I search for a character by its ASCII value?
11.19. How do I search for long lines?
11.20. How do I display all the lines in the current buffer that contain a specified pattern?
11.21. How do I search for a text string that spans multiple lines?
11.22. How do I search for a pattern only within a range of lines in a buffer?

SECTION 12 - CHANGING TEXT

12.1. How do I delete all the trailing white space characters (SPACE and TAB) at the end of all the lines in a file?
12.2. How do I replace all the occurrences of multiple consecutive space characters to a single space?
12.3. How do I reduce a range of empty lines into one line only?
12.4. How do I delete all blank lines in a file? How do I remove all the lines containing only space characters?
12.5. How do I copy/yank the current word?
12.6. How do I yank text from one position to another position within a line, without yanking the entire line?
12.7. When I yank some text into a register, how do I append the text to the current contents of the register?
12.8. How do I yank a complete sentence that spans over more than one line?
12.9. How do I yank all the lines containing a pattern into a buffer?
12.10. How do I delete all the lines in a file that does not contain a pattern?
12.11. How do I add a line before each line with "pattern" in it?
12.12. Is there a way to operate on a line if the previous line contains a particular pattern?
12.13. How do I execute a command on all the lines containing a pattern?
12.14. Can I copy the character above the cursor to the current cursor position?
12.15. How do I insert a blank line above/below the current line without entering insert mode?
12.16. How do I insert the name of current file into the current buffer?
12.17. How do I insert the contents of a Vim register into the current buffer?
12.18. How do I move the cursor past the end of line and insert some characters at some columns after the end of the line?
12.19. How to replace the word under the cursor (say: junk) with "foojunkbar" in Vim?
12.20. How do I replace a particular text in all the files in a directory?

12.21. I have some numbers in a file. How do I increment or decrement the numbers in the file?
12.22. How do I reuse the last used search pattern in a ":substitute" command?
12.23. How do I change the case of a string using the ":substitute" command?
12.24. How do I enter characters that are not present in the keyboard?
12.25. Is there a command to remove any or all digraphs?
12.26. In insert mode, when I press the backspace key, it erases only the characters entered in this instance of insert mode. How do I erase previously entered characters in insert mode using the backspace key?
12.27. I have a file which has lines longer than 72 characters terminated with "+" and wrapped to the next line. How can I quickly join the lines?
12.28. How do I paste characterwise yanked text into separate lines?
12.29. How do I change the case (uppercase, lowercase) of a word or a character or a block of text?
12.30. How do I enter ASCII characters that are not present in the keyboard?
12.31. How do I replace non-printable characters in a file?
12.32. How do I remove duplicate lines from a buffer?
12.33. How do I prefix all the lines in a file with the corresponding line numbers?
12.34. How do I exchange (swap) two characters or words or lines?
12.35. How do I change the characters used as word delimiters?

SECTION 13 - COMPLETION IN INSERT MODE
13.1. How do I complete words or lines in insert mode?
13.2. How do I complete file names in insert mode?
13.3. I am using CTRL-P/CTRL-N to complete words in insert mode. How do I complete words that occur after the just completed word?

SECTION 14 - TEXT FORMATTING
14.1. How do I format a text paragraph so that a new line is inserted at the end of each wrapped line?
14.2. How do I format long lines in a file so that each line contains less than 'n' characters?
14.3. How do I join short lines to the form a paragraph?
14.4. How do I format bulleted and numbered lists?
14.5. How do I indent lines in insert mode?
14.6. How do I format/indent an entire file?
14.7. How do I increase or decrease the indentation of the current line?
14.8. How do I indent a block/group of lines?
14.9. When I indent lines using the > or < key, the standard 8-tabstops are used instead of the current 'tabstop' setting. Why?
14.10. How do I turn off the automatic indentation of text?
14.11. How do I configure Vim to automatically set the 'textwidth' option to a particular value when I edit mails?
14.12. Is there a way to make Vim auto-magically break lines?
14.13. I am seeing a lot of ^M symbols in my file. I tried setting the 'fileformat' option to 'dos' and then 'unix' and then 'mac'. None of these helped. How can I hide these symbols?
14.14. When I paste some text into a Vim buffer from another application, the alignment (indentation) of the new text is messed up. How do I fix this?
14.15. When there is a very long wrapped line (wrap is "on") and a line

doesn't fit entirely on the screen it is not displayed at all. There are blank lines beginning with '@' symbol instead of wrapped line. If I scroll the screen to fit the line the '@' symbols disappear and the line is displayed again. What Vim setting control this behavior?
14.16. How do I convert all the tab characters in a file to space characters?
14.17. What Vim options can I use to edit text that will later go to a word processor?
14.18. How do I join lines without adding or removing any space characters?

SECTION 15 - VISUAL MODE
15.1. How do I do rectangular block copying?
15.2. How do I delete or change a column of text in a file?
15.3. How do I apply an ex-command on a set of visually selected lines?
15.4. How do I execute an ex command on a column of text selected in Visual block mode?
15.5. How do I select the entire file in visual mode?
15.6. When I visually select a set of lines and press the > key to indent the selected lines, the visual mode ends. How can I reselect the region for further operation? (or) How do I re-select the last selected visual area again?
15.7. How do I jump to the beginning/end of a visually selected region?
15.8. When I select text with mouse and then press : to enter an ex command, the selected text is replaced with the : character. How do I execute an ex command on a text selected using the mouse similar to the text selected using the visual mode?
15.9. When I select a block of text using the mouse, Vim goes into selection mode instead of Visual mode. Why?

SECTION 16 - COMMAND-LINE MODE
16.1. How do I use the name of the current file in the command mode or an ex command line?
16.2. How do I edit the text in the Vim command-line effectively?
16.3. How do I switch from Vi mode to Ex mode?
16.4. How do I copy the output from an ex-command into a buffer?
16.5. When I press the tab key to complete the name of a file in the command mode, if there are more than one matching file names, then Vim completes the first matching file name and displays a list of all matching filenames. How do I configure Vim to only display the list of all the matching filenames and not complete the first one?
16.6. How do I copy text from a buffer to the command line and from the command line to a buffer?
16.7. How do I put a command onto the command history without executing it?
16.8. How do I increase the height of the command-line?

SECTION 17 - VIMINFO
17.1. When I invoke Vim, I get error messages about illegal characters in the viminfo file. What should I do to get rid of these messages?
17.2. How do I disable the viminfo feature?
17.3. How do I save and use Vim marks across Vim sessions?

SECTION 18 - REMOTE EDITING
18.1. How do I open a file with existing instance of gvim? What

happened to the Vim 5.x OpenWithVim.exe and SendToVim.exe files?
18.2. How do I send a command to a Vim server to write all buffers to disk?
18.3. Where can I get the documentation about the Vim remote server functionality?

SECTION 19 - OPTIONS

19.1. How do I configure Vim in a simple way?
19.2. How do I toggle the value of an option?
19.3. How do I set an option that affects only the current buffer/window?
19.4. How do I use space characters for a Vim option value?
19.5. Can I add (embed) Vim option settings to the contents of a file?
19.6. How do I display the line numbers of all the lines in a file?
19.7. How do I change the width of the line numbers displayed using the "number" option?
19.8. How do I display (view) all the invisible characters like space, tabs and newlines in a file?
19.9. How do I configure Vim to always display the current line and column number?
19.10. How do I display the current Vim mode?
19.11. How do I configure Vim to show pending/partial commands on the status line?
19.12. How do I configure the Vim status line to display different settings/values?
19.13. How do I configure Vim to display status line always?
19.14. How do I make a Vim setting persistent across different Vim invocations/instances/sessions?
19.15. Why do I hear a beep (why does my window flash) about 1 second after I hit the Escape key?
19.16. How do I make the 'c' and 's' commands display a '$' instead of deleting the characters I'm changing?
19.17. How do I remove more than one flag using a single ":set" command from a Vim option?

SECTION 20 - MAPPING KEYS

20.1. How do I know what a key is mapped to?
20.2. How do list all the user-defined key mappings?
20.3. How do I unmap a key?
20.4. I am not able to create a mapping for the <xxx> key. What is wrong?
20.5. How do I map the numeric keypad keys?
20.6. How do I create a mapping that works only in visual mode?
20.7. In a Vim script, how do I know which keys to use for my mappings, so that the mapped key will not collide with an already used key?
20.8. How do I map the escape key?
20.9. How do I map a key to perform nothing?
20.10. I want to use the Tab key to indent a block of text and Shift-Tab key to unindent a block of text. How do I map the keys to do this? This behavior is similar to textpad, visual studio, etc.
20.11. In my mappings the special characters like <CR> are not recognized. How can I configure Vim to recognize special characters?
20.12. How do I use the '|' to separate multiple commands in a map?
20.13. If I have a mapping/abbreviation whose ending is the beginning of another mapping/abbreviation, how do I keep the first from expanding into the second one?

20.14. Why does it take a second or more for Vim to process a key, sometimes when I press a key?
20.15. How do I map a key to run an external command using a visually selected text?
20.16. How do I map the Ctrl-I key while still retaining the functionality of the <Tab> key?

SECTION 21 - ABBREVIATIONS
21.1. How do I auto correct misspelled words?
21.2. How do I create multi-line abbreviations?
21.3. When my abbreviations are expanded, an additional space character is added at the end of the expanded text. How do I avoid this character?
21.4. How do I insert the current date/time stamp into the file?
21.5. How do I prevent an abbreviation from expanding in insert mode?

SECTION 22 - RECORD AND PLAYBACK
22.1. How do I repeat an editing operation (insertion, deletion, paste, etc)?
22.2. How I record and repeat a set of key sequences?
22.3. How do I edit/modify a recorded set of key sequences?
22.4. How do I write recorded key sequences to a file?
22.5. I am using register 0 to record my key sequences (i.e. q0 .... q). In the recorded key sequences, I am yanking some text. After the first replay of the recorded key sequence, I am no longer able to play it back.

SECTION 23 - AUTOCOMMANDS
23.1. How do I execute a command when I try to modify a read-only file?
23.2. How do I execute a command every time when entering a buffer?
23.3. How do I execute a command every time when entering a window?
23.4. From an autocmd, how can I determine the name of the file or the buffer number for which the autocommand is executed?
23.5. How do I automatically save all the changed buffers whenever Vim loses focus?
23.6. How do I execute/run a function when Vim exits to do some cleanup?

SECTION 24 - SYNTAX HIGHLIGHT
24.1. How do I turn off/on syntax highlighting?
24.2. How do I change the background and foreground colors used by Vim?
24.3. How do I change the highlight colors to suit a dark/light background?
24.4. How do I change the color of the line numbers displayed when the ":set number" command is used?
24.5. How do I change the background color used for a Visually selected block?
24.6. How do I highlight the special characters (tabs, trailing spaces, end of line, etc) displayed by the 'list' option?
24.7. How do I specify a colorscheme in my .vimrc/.gvimrc file, so that Vim uses the specified colorscheme everytime?
24.8. Vim syntax highlighting is broken. When I am editing a file, some parts of the file is not syntax highlighted or syntax highlighted incorrectly.
24.9. Is there a built-in function to syntax-highlight the corresponding matching bracket?
24.10. How do I turn off the C comment syntax highlighting?
24.11. How do I add my own syntax extensions to the standard syntax

files supplied with Vim?
24.12. How do I replace a standard syntax file that comes with the Vim distribution with my own syntax file?
24.13. How do I highlight all the characters after a particular column?
24.14. How do I convert a source file (.c, .h, etc) with the Vim syntax highlighting into a HTML file?
24.15. How do I list the definition of all the current highlight groups?

SECTION 25 - VIM SCRIPT WRITING
25.1. How do I list the names of all the scripts sourced by Vim?
25.2. How do I debug Vim scripts?
25.3. How do I locate the script/plugin which sets a Vim option?
25.4. I am getting some error/informational messages from Vim (possibly when running a script), the messages are cleared immediately. How do I display the messages again?
25.5. How do I save and restore a plugin specific information across Vim invocations?
25.6. How do I start insert mode from a Vim function?
25.7. How do I change the cursor position from within a Vim function?
25.8. How do I check the value of an environment variable in the .vimrc file?
25.9. How do I check whether an environment variable is set or not from a Vim function?
25.10. How do I call/use the Vim built-in functions?
25.11. I am using some normal mode commands in my Vim script. How do I avoid using the user-defined mappings for these normal mode commands and use the standard Vim functionality for these normal mode commands?
25.12. How do I get a visually selected text into a Vim variable or register?
25.13. I have some text in a Vim variable 'myvar'. I would like to use this variable in a ":s" substitute command to replace a text 'mytext'. How do I do this?
25.14. A Vim variable (bno) contains a buffer number. How do I use this variable to open the corresponding buffer?
25.15. How do I store the value of a Vim option into a Vim variable?
25.16. I have copied and inserted some text into a buffer from a Vim function. How do I indent the inserted text from the Vim function?
25.17. How do I get the character under the cursor from a Vim script?
25.18. How do I get the name of the current file without the extension?
25.19. How do I get the basename of the current file?
25.20. How do I get the output from a Vim function into the current buffer?
25.21. How do I call external programs from a Vim function?
25.22. How do I get the return status of a program executed using the ":!" command?
25.23. How do I determine whether the current buffer is modified or not?
25.24. I would like to use the carriage return character in a normal command from a Vim script. How do I specify the carriage return character?
25.25. How do I split long lines in a Vim script?
25.26. When I try to "execute" my function using the "execute 'echo Myfunc()'" command, the cursor is moved to the top of the current buffer. Why?
25.27. How do I source/execute the contents of a register?

25.28. After calling a Vim function or a mapping, when I press the 'u' key to undo the last change, Vim undoes all the changes made by the mapping/function. Why?
25.29. How can I call a function defined with s: (script local function) from another script/plugin?
25.30. Is it possible to un-source a sourced script? In otherwords, reverse all the commands executed by sourcing a script.

SECTION 26 - PLUGINS
26.1. How do I set different options for different types of files?
26.2. I have downloaded a Vim plugin or a syntax file or a indent file, or a color scheme or a filetype plugin from the web. Where should I copy these files so that Vim will find them?
26.3. How do I extend an existing filetype plugin?
26.4. How do I turn off loading the Vim plugins?
26.5. How do I turn on/off loading the filetype plugins?
26.6. How do I override settings made in a file type plugin in the global ftplugin directory for all the file types?
26.7. How do I disable the Vim directory browser plugin?
26.8. How do I set the filetype option for files with names matching a particular pattern or depending on the file extension?

SECTION 27 - EDITING PROGRAM FILES
27.1. How do I enable automatic indentation for C/C++ files?
27.2. How do I configure the indentation used for C/C++ files?
27.3. How do I turn off the automatic indentation feature?
27.4. How do I change the number of space characters used for the automatic indentation?
27.5. I am editing a C program using Vim. How do I display the definition of a macro or a variable?
27.6. I am editing a C program using Vim. How do I jump to the beginning or end of a code block from within the block?
27.7. Is there a way to turn off the "//" comment auto-insertion behavior for C++ files? If I'm sitting on a line beginning with "//", then I open a new line above or below it, Vim automatically inserts new "//" chars.
27.8. How do I add the comment character '#' to a set of lines at the beginning of each line?
27.9. How do I edit a header file with the same name as the corresponding C source file?
27.10. How do I automatically insert comment leaders while typing comments?

SECTION 28 - QUICKFIX
28.1. How do I build programs from Vim?
28.2. When I run the make command in Vim I get the errors listed as the compiler compiles the program. When it finishes this list disappears and I have to use the :clist command to see the error message again. Is there any other way to see these error messages?

SECTION 29 - FOLDING
29.1. How do I extend the Vim folding support?
29.2. When I enable folding by setting the 'foldmethod' option, all the folds are closed. How do I prevent this?
29.3. How do I control how many folds will be opened when I start editing a file?
29.4. How do I open and close folds using the mouse?

29.5. How do I change the text displayed for a closed fold?
29.6. How do I store and restore manually created folds across different Vim invocations?

SECTION 30 - VIM WITH EXTERNAL APPLICATIONS
30.1. Can I run a shell inside a Vim window?
30.2. How do I pass the word under the cursor to an external command?
30.3. How do I get the output of a shell command into a Vim buffer?
30.4. How do I pipe the contents of the current buffer to an external command and replace the contents of the buffer with the output from the command?
30.5. How do I sort a section of my file?
30.6. Is there a step-by-step guide for using Vim with slrn?
30.7. How do I use Vim as a pager?
30.8. How do I view Unix man pages from inside Vim?
30.9. How do I change the diff command used by the Vim diff support?
30.10. How do I use the Vim diff mode without folding?

SECTION 31 - GUI VIM
31.1. How do I create buffer specific menus?
31.2. How do I change the font used by GUI Vim?
31.3. When starting GUI Vim, how do I specify the location of the GVIM window?
31.4. How do I add a horizontal scrollbar in GVim?
31.5. How do I make the scrollbar appear in the left side by default?
31.6. How do I remove the Vim menubar?
31.7. I am using GUI Vim. When I press the ALT key and a letter, the menu starting with that letter is selected. I don't want this behavior as I want to map the ALT-<key> combination. How do I do this?
31.8. Is it possible to scroll the text by dragging the scrollbar so that the cursor stays in the original location?
31.9. How do I get gvim to start browsing files in a particular directory when using the ":browse" command?
31.10. For some questions, like when a file is changed outside of Vim, Vim displays a GUI dialog box. How do I replace this GUI dialog box with a console dialog box?
31.11. I am trying to use GUI Vim as the editor for my xxx application. When the xxx application launches GUI Vim to edit a file, the control immediately returns to the xxx application. How do I start GUI Vim, so that the control returns to the xxx application only after I quit Vim?
31.12. Why does the "Select Font" dialog doesn't show all the fonts installed in my system?
31.13. How do I use the mouse in Vim command-line mode?
31.14. When I use the middle mouse button to scroll text, it pastes the last copied text. How do I disable this behavior?
31.15. How do I change the location and size of a GUI Vim window?

SECTION 32 - VIM ON UNIX
32.1. I am running Vim in a xterm. When I press the CTRL-S key, Vim freezes. What should I do now?
32.2. I am seeing weird screen update problems in Vim. What can I do to solve this screen/display update problems?
32.3. I am using the terminal/console version of Vim. In insertmode, When I press the backspace key, the character before the cursor is not erased. How do I configure Vim to do this?
32.4. I am using Vim in a xterm. When I quit Vim, the screen contents

are restored back to the original contents. How do I disable this?
32.5. When I start Vim, it takes quite a few seconds to start. How do I minimize the startup time?
32.6. How can I make the cursor in gvim in unix stop blinking?
32.7. How do I change the menu font on GTK Vim?
32.8. How do I prevent <Ctrl-Z> from suspending Vim?
32.9. When I kill the xterm running Vim, the Vim process continues to run and takes up a lot of CPU (99%) time. Why is this happening?
32.10. How do I get the Vim syntax highlighting to work in a Unix terminal?

SECTION 33 - VIM ON MS-WINDOWS
33.1. In MS-Windows, CTRL-V doesn't start the blockwise visual mode. What happened?
33.2. When I press the CTRL-Y key, it acts like the CTRL-R key. How do I configure Vim to treat CTRL-Y as CTRL-Y?
33.3. How do I start GUI Vim in a maximized window always?
33.4. After doing some editing operations, Vim freezes. The cursor becomes an empty rectangle. I am not able enter any characters. What is happening?
33.5. I am using Windows XP, the display speed of maximized GVim is very slow. What can I do to speed the display updates?
33.6. What are the recommended settings for using Vim with cygwin?
33.7. I am trying to use GNU diff with Vim diff mode. When I run the diff from command line, it works. When I try to use the diff with Vim it doesn't work. What should I do now?
33.8. Is it possible to use Vim as an external editor for MS-Windows Outlook email client?
33.9. I am using Vim to edit HTML files. How do I start internet explorer with the current file to preview the HTML file?
33.10. I would like to use Vim with Microsoft Visual Studio. How do I do this?
33.11. Where do I place the _vimrc and _gvimrc files?
33.12. Everytime I save a file, Vim warns about the file being changed outside of Vim. Why?

SECTION 34 - PRINTING
34.1. How do I print a file along with line numbers for all the lines?
34.2. How do I print a file with the Vim syntax highlighting colors?

SECTION 35 - BUILDING VIM FROM SOURCE
35.1. How do I build Vim from the sources on a Unix system?
35.2. How do I install Vim in my home directory or a directory other than the default installation directory in Unix?
35.3. How do I build Vim from the sources on a MS-Windows system?
35.4. The Vim help, syntax, indent files are missing from my Vim installation. How do I install these files?
35.5. I have built Vim from the source and installed the Vim package using "make install". Do I need to keep the Vim source directory?
35.6. How do I determine the Vim features which are enabled at compile time?
35.7. Can I build Vim without the GUI support?
35.8. When building Vim on a Unix system, I am getting "undefined reference to term_set_winsize' error. How do I resolve this error?
35.9. Vim configure keeps complaining about the lack of gtk-config while trying to use GTK 2.03. This is correct, since in GTK 2

they moved to using the generic pkg-config. I can get pkg-config to list the various includes and libs for gtk, but for some reason the configure script still isn't picking this up.

SECTION 36 - VARIOUS
36.1. How do I edit binary files with Vim?
36.2. How do I disable the visual error flash and the error beep?
36.3. How do I display the ascii value of a character displayed in a buffer?
36.4. Can I use zero as a count for a Vim command?
36.5. How do I disable the Vim welcome screen?
36.6. How do I avoid the "hit enter to continue" prompt?
36.7. How do I invoke Vim from command line to run a group of commands on a group of files?
36.8. How do I use a normal mode command from insert mode without leaving the insert mode?
36.9. How do I start Vim in insert mode?

SECTION 37 - UNICODE
37.1. Is it possible to create Unicode files using Vim?
37.2. Which Vim settings are particularly important for editing Unicode files?
37.3. What is the 'encoding' option?
37.4. How does Vim name the various Unicode encodings?
37.5. How does Vim specify the presence or absence of a byte-order mark?
37.6. What is the 'fileencoding' option?
37.7. What is the 'fileencodings' option?
37.8. What is the 'termencoding' option?
37.9. What is the 'bomb' option?
37.10. Where can I find an example of a typical use of all these options?
37.11. How can I insert Unicode characters into a file using Vim?
37.12. How can I know which digraphs are defined and for which characters?

===

SECTION 1 - GENERAL INFORMATION

1.1. What is Vim?

Vim stands for Vi IMproved. It used to be Vi IMitation, but there are so many improvements that a name change was appropriate. Vim is a text editor which includes almost all the commands from the Unix program "Vi" and a lot of new ones. All commands can be given with the keyboard. This has the advantage that you can keep your fingers on the keyboard and your eyes on the screen. For those who want it, there is mouse support and a GUI version with scrollbars and menus.

Vim is an editor, not a word processor. A word processor is used mainly to do layout of text. This means positioning it, changing the way it appears on output. More often than not, the final document is meant to be printed or typeset or what have you, in order to present it in a pleasing manner to others. Examples of word processors are Microsoft Word, WordPerfect, FrameMaker, and AmiPro.

An editor is simply for entering text. Any typesetting or laying out of the document is secondary. With an editor, one's main concern is entering text, not making the text look good. Examples of editors other than Vim and Vi are Emacs, Crisp, Brief, and xedit. And Notepad.

For more information, read

```
:help intro
```

1.2. Who wrote Vim?

Most of Vim was written by Bram Moolenar, with contributions from too many people to mention here. See ":h credits" for a complete list.

Vim is based on Stevie, worked on by Tim Thompson, Tony Andrews and G.R. (Fred) Walter.

For more information, read

```
:help author
```

1.3. Is Vim compatible with Vi?

Yes. Vim is very much compatible with Vi. You can use the "-C" command-line flag to start Vim in Vi compatible mode:

```
$ vim -C
```

You can also use:

```
$ vim -u NONE
```

You can also set the 'compatible' option to enable Vi compatibility:

```
:set compatible
```

For more information, read

```
:help -C
:help 'compatible'
:help compatible-default
```

1.4. What are some of the improvements of Vim over Vi?

A short summary of the improvements of Vim over vi is listed below. The list shows that Vim is a thoroughly modern and feature-packed editor. Standard features of modern editors are implemented, and there is an equal emphasis on general power-user features and features for programmers.

Features to modernise Vi:

Multi-level undo
   Allows you to set the number of times you can undo your changes in a file buffer. You can also redo an undone change.

Multiple windows and buffers
Each file can be displayed in its own window. You can move easily from one window to another. Each file opened during a Vim session also has an associated buffer and you can easily jump from one to the other.
Flexible insert mode
Vim allows you to use the arrow keys while in insert mode to move around in the file. No more hitting <Esc>, moving around, then hitting `i' or `a'.
Macros
Vim has a facility which allows you to record a sequence of typed characters and repeat them any number of times.
Visual mode
You can highlight sections of text and execute operations on this section of text only.
Block operators
Allow selection and highlighting of rectangular blocks of text in order do execute specific operations on them.
Online help system
You can easily find help on any aspect of using Vim. Help is displayed in its own window.
Command-line editing and history
History allows you to use the arrow keys to repeat or search for a command that has already been typed. Allows you to match the beginning of a command with the beginning of another similar command in the history buffer. You can also edit a command to correct typos or change a few values.
Command line completion.
Using the <Tab> key, you can complete commands, options, filenames, etc. as needed.
Horizontal scrolling.
Long lines can be scrolled horizontally (with or without the GUI).

Advanced user features:

Text formatting.
With two keystrokes, you can format large sections of text, without the use of external programs.
Word completion in Insert mode
Vim can complete words while you are typing, by matching the current word with other similar words in the file.
Jump tags
Just like in an internet browser, you can jump back to previous parts of the text you were editing, and then forward again. Your brain is thus free to edit instead of navigate.
Automatic commands
Commands automatically executed when reading or writing a file, jumping to another buffer, etc.
Viminfo
Allows storing of the command line history, marks and registers in a file to be read on startup. Therefore, you can recall old search patterns, macros, etc., in a new Vim session.
Mouse support
The mouse is supported in an xterm and for MS-DOS. It can be used to position the cursor, select the visual area, paste a register, etc.
Graphical User Interface (GUI)
Just like any modern editor. Also, it's very easy to add your own menus. Of course, console vim is still supported, and very widely used.

```
  Scripting language
     Vim has a powerful scripting language so new commands can be created.
     You can also use Perl, Python, TCL and Ruby to achieve the same thing!
  Plugins
     Extra functionality implemented via vim commands (regular commands or
     the scripting language) that is automatically loaded on startup.
     Examples: file explorer, network editing.  More are being developed
     and shared on VimOnline all the time.
  Syntax highlighting for many programming languages
     Syntax highlighting for hundreds of programming languages is
     supported. Support for others can be added.
  Extended regular expressions
     Vim supports extended regular expressions which are similar in
     functionality to that of perl regular expressions.

Programming performance features:

  Edit-compile-edit speedup
     You can compile within Vim and automatically jump to the location of
     errors in the source code.
  Indenting for many programming languages
     C, C++, Java, Perl, XML and many other languages can be automatically
     indented by vim while you type.  Support for others can be added.
  Searching for words in include files
     Vim allows you to search for a match of the word under the cursor in
     the current and included files.
  Advance text objects
     Instantly select, or delete, or copy, or indent, or format, or change
     case, or ... to all the text between ( and ), or { and }, or < and >,
     or [ and ].  Or a word, sentence, or paragraph.  Very powerful.
  Folding
     Certain parts of the text can be "folded" away.  The best example is
     the body of a function.  You can get an overview of the code, and then
     open the fold of the function whose detail you need to see.
  ctags and cscope integration
     Using these two powerful programs, you can jump to a definition of a
     function from a calling instance of it, and use other tricks to
     navigate source code.

For more information, read

    :help vi-differences


1.5. Is Vim free?

Vim is Charityware. There are no restrictions on using or copying Vim, but
the author encourages you to make a donation to charity. A document
explaining how to do so is included in the distribution.

For more information, read

    :help copyright


=============================================================================

SECTION 2 - RESOURCES
```

2.1. Where can I learn more about Vim?

You can post your Vim questions to the vim@vim.org mailing list. You can post your Vim development related questions to the vim-dev@vim.org mailing list. Vim does not have a newsgroup of its own. But the appropriate newsgroup to post to is comp.editors.

"VimOnline" is a web page that serves as a de facto homepage for vim, although the main purpose of it is to gather tips and scripts from everywhere. Get involved! The URL is vim.sourceforge.net or vim.sf.net.

Finally, read the Vi FAQ:

    http://www.faqs.org/faqs/editor-faq/vi/part1/index.html

For more information, read

```
:help mail-list
:help internet
```

2.2. Is there a mailing list available?

There are several:

NAME	DESCRIPTION
vim-announce	Announcements of new releases
vim	General discussion
vim-dev	Patches, bug reports, development discussions
vim-mac	Macintosh discussion
vim-fr	General discussion in French
vim-multibyte	Multibyte development issues
vim-vms	Development on VMS

Of these, only vim and vim-dev are of general interest. vim-announce is read-only to most people, and its messages are sent to the other lists as well. The remaining four are very low volume.

To subscribe: send an email to <NAME>-subscribe@vim.org
To unsubscribe: send an email to <NAME>-unsubscribe@vim.org
To get help: send an email to <NAME>-help@vim.org

2.3. Is there an archive available for the Vim mailing lists?

Yes. Visit http://www.yahoogroups.com/list/<name>, where name is one of: vimannounce, vim, vimdev, vim-fr, vim-mac, vim-multibyte, vim-vms.

Alternatively, visit www.gmane.org to find out about GMANE, which allows you to access the mailing lists as though they were newsgroups. This offers some convenience to those who wish to browse the history or casually observe the current threads.

2.4. Where can I get the Vim user manual in HTML/PDF/PS format?

You can download the HTML/PDF/PS format of the Vim user manual from:

    http://vimdoc.sourceforge.net/

2.5. I have a "xyz" (some) problem with Vim. How do I determine it is a problem with my setup or with Vim?

First, you have to determine that the problem is not with your .vimrc or .gvimrc or system vimrc or your personal plugin files or in any of your setup files. To do this, use

```
$ vim -N -u NONE -U NONE
```

This will start Vim in 'nocompatible" mode and will not source your personal .vimrc and .gvimrc files. It will also not load your personal plugins. In this invocation of Vim, try to reproduce your problem. If you are not able to reproduce the problem, then the problem is related to some setting in one of your local setup files or plugins. To locate the problem in your setup files, you have to use trial and error and try commenting out the lines in your setup files one by one. You can also use the -V command line argument to Vim to get more debug information and analyze the problem:

```
$ vim -V2
```

You can increase the value passed to the -V argument to get more debug information.

For more information, read

```
:help -u
:help -U
:help -N
:help -V
:help 'verbose'
:help :verbose
:help set-verbose
```

2.6. Where can I report bugs?

First collect the required information using the following command:

```
:source $VIMRUNTIME/bugreport.vim
```

Now send the resulting text from the above command to the bugs@vim.org e-mail address.

The Vim Development mailing list (see above) is a good place to discuss general bugs. If the bug you find is with syntax highlighting or some other "added feature" (i.e. not directly programmed into vim), attempt to inform the maintainer of that feature.

For more information, read

```
:help bug-reports
```

2.7. Where can the FAQ be found?

The FAQ can be found at VimOnline (vim.sf.net). Other places will be decided in the future.

2.8. What if I don't find an answer in this FAQ?

This FAQ covers mainly Vim-specific questions. You may find more information suitable for most Vi clones by reading the Vi FAQ. It is posted regularly on comp.editors. You can also find a copy at

    http://www.faqs.org/faqs/editor-faq/vi/part1/index.html

Also, since Vim has gathered so many features in the last few years, successfully documenting the frequently asked questions here is a near-impossible task. To make it possible, please email the maintainer if you have a good question. A good question is one that you've tried to answer yourself (remember, Vim has great documentation) but struggled.

2.9. I have a patch for implementing a Vim feature. Where can I send this patch?

You can send your patches to the Vim developer mailing list vim-dev@vim.org.

For more information, read

    :help vim-dev

2.10. I have a Vim tip or developed a new Vim syntax/indent/filetype/compiler plugin or developed a new script or a colorscheme. Is there a public website where I can upload this?

Yes. You can use the Vim Online website to upload your plugins/scripts, colorschemes, tips, etc. The site is at http://vim.sourceforge.net

===

SECTION 3 - AVAILABILITY

3.1. What is the latest version of Vim?

The latest version of Vim is 6.3 released on 8th June 2004.

The release-history of different versions of Vim is below:

```
Version 6.3     8th June 2004
Version 6.2     1st June 2003
Version 6.1     24th March 2002
Version 6.0     27th September, 2001
Version 5.8     31st May, 2001
Version 5.7     24th June, 2000
```

```
Version 5.6     16th January, 2000
Version 5.5     21st September, 1999
Version 5.4     26th July, 1999
Version 5.3     31st August, 1998
Version 5.2     24th August, 1998
Version 5.1     7th April, 1998
Version 5.0     19th February, 1998
Version 4.6     13th March,1997
Version 4.5     17th October, 1996
Version 4.2     5th July,1996
Version 4.0     21st May, 1996
Version 3.0     16th August, 1994
Version 2.0     21st December, 1993
Version 1.27    23rd April, 1993
Version 1.17    21st April, 1992
```

3.2. Where can I find the latest version of Vim?

You can download the sources for the latest version of Vim from the VimOnline website. The URL for this site is http://vim.sourceforge.net/download.php.

3.3. What platforms does it run on?

All Unix platforms.
All Windows platforms.
Amiga, Atari, BeOS, DOS, Macintosh, MachTen, OS/2, RiscOS, VMS.

3.4. Where can I download the latest version of the Vim runtime files?

You can download the latest version of the Vim runtime files (syntax files, filetype plugins, compiler files, color schemes, documentation, indentation files and keymaps) from the Vim ftp site from the ftp://ftp.vim.org/pub/vim/runtime directory.

===

SECTION 4 - HELP

4.1. How do I use the help files?

Help can be found for all functions of Vim. In order to use it, use the ":help" command. This will bring you to the main help page. On that first page, you will find explanations on how to move around. Basically, you move around in the help pages the same way you would in a read-only document. You can jump to specific subjects by using tags. This can be done in two ways:

* Use the "<Ctrl-]>" command while standing on the name of a command or option. This only works when the tag is a keyword. "<Ctrl-LeftMouse>" and "g<LeftMouse>" work just like "<Ctrl-]>".
* use the ":tag <subject>" command. This works with all characters.

Use "<Ctrl-T>" to jump back to previous positions in the help files. Use ":q" to close the help window.

If you want to jump to a specific subject on the help pages, use ":help {subject}". If you don't know what to look for, try ":help index" to get a list of all available subjects. Use the standard search keys to locate the information you want. You can abbreviate the ":help" command as ":h".

For more information, read

```
:help online-help
```

4.2. How do I search for a keyword in the Vim help files?

You can press the CTRL-D key after typing the help keyword to get a list of all the help keywords containing the supplied pattern. You can also use the meta characters like *, \+, etc to specify the help search pattern:

```
:help init<C-D>
:help str*()<C-D>
:help '*indent<C-D>
```

You can press the Tab key after typing a partial help keyword to expand to the matching keyword. You can continue to press the Tab key to see other keyword matches.

From the help window, you can use the ":tag" command to search for keywords. For example,

```
:tselect /window
```

This command will list all the help keywords containing the text "window". You can select one from the list and jump to it.

You can use the ":helpgrep" command to search for the given text in all the help files. The quickfix window will be opened with all the matching lines.

For more information, read

```
:help c_CTRL-D
:help c_<Tab>
:help :tselect
:help :help
:help :helpgrep
```

4.3. I am getting an error message E123, what did I do wrong?

You can get more information about the error and the error message using:

```
:help E123
```

For more information, read

```
:help error-messages
```

4.4. Where can I read about the various modes in Vim?

You can get information about the different modes in Vim by reading

```
:help vim-modes
```

4.5. How do I generate the Vim help tags file after adding a new Vim help file?

You can use the ":helptags" command to regenerate the Vim help tag file. For example:

```
:cd $VIMRUNTIME/doc
:helptags .
```

For more information, read

```
:help :helptags
:help add-local-help
```

4.6. Can I use compressed versions of the help files?

Yes. You can compress the help files and still be able to view them with Vim. This makes accessing the help files a bit slower and requires the "gzip" utility. Follow these steps to compress and use the Vim help files:

- Compress all the help files using "gzip doc/*.txt".

- Edit the "doc/tags" file and change the ".txt" to ".txt.gz" using

```
        :%s=\(\t.*\.txt\)\t=\1.gz\t=
```

- Add the following line to your vimrc:

```
        set helpfile={dirname}/help.txt.gz
```

Where {dirname} is the directory where the help files are. The gzip.vim plugin supplied with the standard Vim distribution will take care of decompressing the files. You must make sure that $VIMRUNTIME is set to where the other Vim files are, when they are not in the same location as the compressed "doc" directory.

For more information, read

```
:help gzip-helpfile
:help 'helpfile'
:help gzip
:help $VIMRUNTIME
```

===

SECTION 5 - EDITING A FILE

5.1. How do I load a file in Vim for editing?

There are several ways to load a file for editing. The simplest is to

use the ":e" (:edit) command:

```
:e <filename>
```

You can also use the ":n" (:next) command to load files into Vim:

```
:n <filename(s)>
```

You can also use the ":args" command to load files into Vim:

```
:args <filename(s)>
```

For more information, read

```
:help usr_07
:help edit-files
:help :edit
:help :next_f
:help :args_f
```

5.2. How do I save the current file in another name (save as) and edit a new file?

You can use the ":saveas" command to save the current file in another name:

```
:saveas <newfilename>
```

Alternatively, you can also use the following commands:

```
:w <newfilename>
:edit #
```

You can also use the ":file" command, followed by ":w" command:

```
:file <newfilename>
:w
```

For more information, read

```
:help 07.7
:help :saveas
:help :file_f
:help :w
```

5.3. How do I change the current directory to the directory of the current file?

You can use the following command to change the current directory to the directory of the current file:

```
:cd %:p:h
```

To automatically change the current directory to the directory of the current file, use the following autocmd:

```
:autocmd BufEnter * cd %:p:h
```

For more information, read

```
:help :cd
:help :lcd
:help filename-modifiers
:help autocommand
```

5.4. How do I write a file without the line feed (EOL) at the end of the file?

You can turn off the 'eol' option and turn on the 'binary' option to write a file without the EOL at the end of the file:

```
:set binary
:set noeol
:w
```

For more information, read

```
:help 'endofline'
:help 'binary'
:help 23.4
```

5.5. How do I configure Vim to open a file at the last edited location?

Vim stores the cursor position of the last edited location for each buffer in the '"' register. You can use the following autocmd in your .vimrc or .gvimrc file to open a file at the last edited location:

```
au BufReadPost * if line("'\"") > 0 && line("'\"") <= line("$") |
                     \ exe "normal g'\"" | endif
```

For more information, read

```
:help '"
:help last-position-jump
```

5.6. When editing a file in Vim, which is being changed by an external application, Vim opens a warning window (like the confirm dialog) each time a change is detected. How do I disable this warning?

You can set the Vim 'autoread' option to automatically read the file again when it is changed outside of Vim:

```
:set autoread
```

You can also use the following autocommand:

```
autocmd FileChangedShell *
      \ echohl WarningMsg |
      \ echo "File has been changed outside of vim." |
      \ echohl None
```

For more information, read

```
    :help 'autoread'
    :help FileChangedShell
    :help timestamp
    :help :checktime
```

5.7. How do I edit a file whose name is under the cursor?

You can use the gf command to edit a file whose name is under the cursor.
You can use the CTRL-W f command to edit the file in a new window.

For more information, read

```
    :help gf
    :help CTRL-W_f
    :help 'isfname'
    :help 'path'
    :help 'suffixesadd'
    :help 'includeexpr'
```

5.8. How do I reload/re-edit the current file?

You can use the ":edit" command, without specifying a file name, to reload the current file. If you have made modifications to the file, you can use ":edit!" to force the reload of the current file (you will lose your modifications).

For more information, read

```
    :help :edit
    :help :edit!
    :help 'confirm'
```

5.9. How do I autosave a file periodically?

Vim doesn't support auto-saving a file periodically.

For more information, read

```
    :help 'updatetime'
    :help CursorHold
    :help swap-file
```

5.10. How do I open a file in read-only mode?

You can open a file in read-only mode using the ":view" command:

```
    :view <filename>
```

This command sets the 'readonly' option for the opened buffer. You can also use the "-R" command-line option to open a file in read-only mode:

```
    $ vim -R <filename>
```

You can also use the symbolic link executable "view" to open a file in read-only mode from the command-line:

```
$ view <filename>
```

For more information, read

```
:help 07.6
:help 'readonly'
:help 'modifiable'
:help :view
:help :sview
:help view
:help -R
:help -M
```

5.11. How do I open a file for editing without saving the modifications to the current file?

You can open a file for editing without saving the modifications to the current file and without losing the changes using one of the following methods:

```
:split <new_filename>
:new <new_filename>
```

You can also set the 'hidden' option and edit a new file:

```
:set hidden
: e <new_filename>
```

If you want to discard the changes made to the current file and load another file for editing, then you can use the following command:

```
:e! <new_filename>
```

For more information, read

```
:help :edit!_f
:help 'hidden'
:help :split
:help :new
```

===

SECTION 6 - EDITING MULTIPLE FILES

6.1. How do I open multiple files at once from within Vim?

There are several ways to open multiple files at once from within Vim. You can use the ":next" command to specify a group of files:

```
:next f1.txt f2.txt
:next *.c
```

You can use the :args command to specify a group of files as arguments:

```
:args f1.txt f2.txt
:args *.c
```

After loading the files, you can use the ":next" and ":prev" command to switch between the files.

For more information, read

```
:help 07.2
:help :next
:help :args_f
:help argument-list
```

6.2. How do I switch between multiple files/buffers in Vim?

There are several ways to switch between multiple files. You can use the ":buffer" command to switch between multiple files. For example,

```
:buffer file1
:buffer file2
```

You can also use the CTRL-^ key to switch between buffers. By specifying a count before pressing the key, you can edit the buffer with that number. Without the count, you can edit the alternate buffer by pressing CTRL-^

You can also use the ":e #" command to edit a particular buffer:

```
:e #5
```

For more information, read

```
:help edit-files
:help :buffer
:help CTRL-^
:help alternate-file
:help 22.4
:help 07.3
```

6.3. How do I open several files in Vim, with each file in a separate window?

You can use the -o and -O Vim command line arguments to open multiple files in separate horizontally or vertically split Vim windows. For example:

```
$ vim -o3 f1.txt f2.txt f3.txt
```

The above command will open the files f1.txt, f2.txt and f3.txt in three separate horizontally split Vim windows.

```
$ vim -O3 f1.txt f2.txt f3.txt
```

The above command will open the files f1.txt, f2.txt and f3.txt in three separate vertically split Vim windows.

For more information, read

```
:help -o
:help -O
:help startup-options
```

6.4. How do I configure Vim to autoload several files at once similar to "work-sets" or "projects"?

You can use the ":mksession" and the ":mkview" commands to autoload several files in Vim.

The ":mksession" command creates a Vim script that restores the current editing session. You can use the ":source" command to source the file produced by the mksession command.

The ":mkview" command creates a Vim script that restores the contents of the current window. You can use the ":loadview" command to load the view for the current file.

For more information, read

```
:help 21.4
:help 21.5
:help views-sessions
:help 'sessionoptions'
:help :mksession
:help :source
:help v:this_session
:help :mkview
:help :loadview
:help 'viewdir'
:help buffers
```

6.5. Is it possible to open multiple top level windows in a single instance of Vim similar to Nedit or emacs?

No. It is currently not possible to open multiple top-level windows in a single instance of Vim. This feature is in the todo list.

6.6. How do I browse/explore directories from within Vim?

You can use the explorer.vim plugin, supplied with the standard Vim installation, to browse/explore directories from within Vim. You can start the file explorer using one of the following commands:

```
:e <directory>
:Explore
:SExplore
```

From the file explorer, you can browse through directories, rename, delete and edit files.

For more information, read

```
    :help file-explorer
    :help 22.1
```

6.7. How do I edit files over a network using ftp/scp/rcp/http?

You can use the netrw.vim plugin, supplied with the standard Vim package, to edit files over a network using ftp/scp/rcp/http. Using this plugin, Vim will transparently load and save the files over ftp/scp/rcp/http. For example, to edit a file over ftp, you can use the following command:

```
    $ vim ftp://machine/path
```

For more information, read

```
    :help netrw.vim
```

===

SECTION 7 - BACKUP

7.1. When I edit and save files, Vim creates a file with the same name as the original file and a "~" character at the end. How do I stop Vim from creating this file? (or) How do I disable the Vim backup file feature?

You have set the 'backup' option, so Vim creates a backup file when saving the original file. You can stop Vim from creating the backup file, by clearing the option:

```
    :set nobackup
```

Note that, by default this option is turned off. You have explicitly enabled the 'backup' option in one of the initialization files. You may also have to turn off the 'writebackup' option:

```
    :set nowritebackup
```

For more information, read

```
    :help 07.4
    :help backup-table
    :help 'backup'
    :help 'writebackup'
    :help 'backupskip'
    :help 'backupdir'
    :help 'backupext'
    :help 'backupcopy'
    :help backup
```

7.2. How do I configure Vim to store all the backup files in a particular directory?

You can configure Vim to store all the backup files in a particular directory using the 'backupdir' option. For example, to store all the

backup files in the ~/backup directory, you can use the following command:

```
:set backupdir=~/backup
```

For more information, read

```
:help 07.4
:help 'backupdir'
:help backup
```

7.3. When I save a file with Vim, the file permissions are changed.
How do I configure Vim to save a file without changing the file
permissions?

This may happen, if the 'backupcopy' option is set to 'no' or 'auto'. Note that the default value for this option is set in such a way that this will correctly work in most of the cases. If the default doesn't work for you, try setting the 'backupcopy' option to 'yes' to keep the file permission when saving a file:

```
:set backupcopy=yes
```

This applies, only if you have configured Vim to make a backup whenever overwriting a file. By default, Vim will not backup files.

For more information, read

```
:help 'backupcopy'
:help backup
:help 'backup'
:help 'writebackup'
```

===

SECTION 8 - BUFFERS

8.1. I have made some modifications to a buffer. How do I edit another
buffer without saving the modified buffer and also without losing the
modifications?

You can set the 'hidden' option to edit a file without losing modifications to the current file:

```
:set hidden
```

By setting the 'hidden' option, you can also save the modification history (undo-history) for the buffer. Otherwise, as you switch between files, the undo-history will be lost.

For more information, read

```
:help 'hidden'
:help hidden-quit
:help :hide
```

8.2. How do I configure Vim to auto-save a modified buffer when switching to another buffer?

You can set the 'autowrite' option to auto-save a modified buffer when switching to another buffer:

```
:set autowrite
```

For more information, read

```
:help 'autowrite'
:help 'autowriteall'
:help 'hidden'
```

8.3. How do I replace the buffer in the current window with a blank buffer?

You can use the ":enew" command to load an empty buffer in place of the buffer in the current window.

For more information, read

```
:help :enew
```

8.4. Is there a keyboard shortcut to load a buffer by the buffer number?

You can use the CTRL-^ command to load a buffer by specifying the buffer number. For example, to load buffer number 5, you have to use the 5 CTRL-^ command.

For more information, read

```
:help CTRL-^
```

8.5. How do I open all the current buffers in separate windows?

You can use the ":ball" or ":sball" commands to open all the buffers in the buffer list:

```
:ball
```

For more information, read

```
:help :ball
```

8.6. How do I close (delete) a buffer without exiting Vim?

You can use the ":bdelete" command to delete a buffer without exiting Vim. For example:

```
:bdelete file1
```

For more information, read

```
:help :bdelete
:help :bwipeout
```

8.7. I have several buffers opened with :e filename. How do I close one of the buffers without exiting Vim?

You can use the ":bdelete <buffername>" command to close the buffer.

For more information, read

```
:help :bdelete
:help :bunload
:help :bwipeout
```

8.8. When I use the command ":%bd" to delete all the buffers, not all the buffers are deleted. Why?

In the ":%bd" command, the '%' range will be replaced with the starting and ending line numbers in the current buffer. Instead of using '%' as the range, you should specify numbers for the range. For example, to delete all the buffers, you can use the command ":1,9999bd".

For more information, read

```
:help :bd
```

8.9. How do I display the buffer number of the current buffer/file?

You can use 2<CTRL-G> command to display the buffer number for the current file/buffer. Note the use of count before the CTRL-G command. If the count is greater than 1, then Vim will display the buffer number.

You can also use the following command to display the current buffer number:

```
:echo bufnr("%")
```

You can also include the "%n" field to the 'statusline' option to display the current buffer number on the statusline.

For more information read,

```
:help CTRL-G
:help bufnr()
:help :echo
:help 'statusline'
```

8.10. How do I delete a buffer without closing the window in which the buffer is displayed?

You can use the following command to open the next buffer and delete the current buffer.

```
:bnext | bdelete #
```

For more information read,

```
:help :bnext
:help :bdelete
:help :buffers
```

8.11. How do I map the tab key to cycle through and open all the buffers?

You can use the following two map commands, to map the CTRL-Tab key to open the next buffer and the CTRL-SHIFT-Tab key to open the previous buffer:

```
:nnoremap <C-Tab> :bnext<CR>
:nnoremap <S-C-Tab> :bprevious<CR>
```

For more information read,

```
:help :bnext
:help :previous
```

===

SECTION 9 - WINDOWS

9.1. What is the difference between a Vim window and a buffer?

A Vim buffer is a file loaded into memory for editing. The original file remains unchanged until you write the buffer to the file. A Vim window is a viewport onto a buffer. You can use multiple windows on one buffer or several windows on different buffers.

For more information, read

```
:help usr_08.txt
:help 22.4
:help windows-intro
:help Q_wi
```

9.2. How do I increase the width of a Vim window?

You can increase the width of a Vim window using one of the following commands:

```
:vert resize +N
:vert resize -N
:vert resize N
```

You can also use CTRL-W < or CTRL-W > or CTRL-W | commands.

For more information, read

```
:help vertical-resize
:help CTRL-W_>
:help CTRL-W_<
```

```
:help window-resize
```

9.3. How do I zoom into or out of a window?

You can zoom into a window (close all the windows except the current window) using the "CTRL-W o" command or the ":only" ex command.

You can use the "CTRL-W _" command or the ":resize" ex command to increase the current window height to the highest possible without closing other windows.

You can use the "CTRL-W |" command or the ":vertical resize" ex command to increase the current window width to the highest possible without closing other windows.

You can use the "CTRL-W =" command to make the height and width of all the windows equal.

You can also set the following options to get better results with the above commands:

Method 1:
Set the 'winminheight' option to 0:

```
:set winminheight=0
```

By default, this option is set to 1.
This option controls the minimum height of an inactive window (when it is not the current window). When the 'winminheight' option is set to 0, only the status line will be displayed for inactive windows.

Method 2:
Set the 'noequalalways' option and set the 'winheight' option to a large value (like 99999):

```
:set noequalalways
:set winheight=99999
```

Now, the active window will always open to its maximum size, while the other windows will stay present, but shrunken to just a status line.

With any of the above mentioned methods, you cannot restore the window layout after zooming into a window. If you want to restore the Vim window layout after zooming into a window, you can use the ZoomWin plugin. You can download this plugin from the Vim online website at:

http://vim.sourceforge.net/scripts/script.php?script_id=508

For more information, read

```
:help CTRL-W_o
:help window-resize
:help 'winminheight'
:help 'equalalways'
:help 'winheight'
:help 08.3
```

9.4. How do I execute an ex command on all the open buffers or open windows
     or all the files in the argument list?

You can use the ":bufdo" command to execute an ex command on all the open buffers. You can use the ":windo" command to execute an ex command on all the open windows. You can use the ":argdo" command to execute an ex command on all the files specified in the argument list.

For more information, read

```
:help :windo
:help :bufdo
:help :argdo
:help 26.3
```

===

SECTION 10 - MOTION

10.1. How do I jump to the beginning (first line) or end (last line) of a
     file?

You can use 'G' command to jump to the last line in the file and the 'gg' command to jump to the first line in the file.

For more information, read

```
:help G
:help gg
```

10.2. In insert mode, when I press the <Esc> key to go to command mode, the
     cursor moves one character to the left (except when the cursor is on
     the first character of the line). Is it possible to change this
     behavior to keep the cursor at the same column?

No. It is not possible to change this behavior. The cursor is *always* positioned on a valid character (unless you have virtual-edit mode enabled). So, if you are appending text to the end of a line, when you return to command mode the cursor *must* drop back onto the last character you typed. For consistency sake, the cursor drops back everywhere, even if you are in the middle of a line.

You can use the CTRL-O command in insert mode to execute a single ex command and return back to insert mode without moving the cursor column.

For more information, read

```
:help 'virtual'
:help i_CTRL-O
```

10.3. How do I configure Vim to maintain the horizontal cursor position when
     scrolling with the <Page Up>, <Page Down>, etc keys?

You can reset the 'startofline' option to keep the cursor at the same horizontal location when scrolling text:

```
:set nostartofline
```

For more information, read

```
:help 'startofline'
```

10.4. Some lines in a file are more than the screen width and they are all wrapped. When I use the j, k keys to move from one line to the next, the cursor is moved to the next line in the file instead of the next line on the screen. How do I move from one screen line to the next?

You can use the gj and gk commands to move from one screen line to the next/previous screen line. The j and k commands move the cursor from one file line to the next file line. You can also avoid the line wrapping by resetting the 'wrap' option:

```
:set nowrap
```

For more information, read

```
:help gj
:help gk
:help 'wrap'
```

You can use the following mappings:

```
:map <Up> gk
:imap <Up> <C-o>gk
:map <Down> gj
:imap <Down> <C-o>gj
```

10.5. What is the definition of a sentence, paragraph and section in Vim?

A sentence is defined as ending at a '.', '!' or '?' followed by either the end of a line, or by a space (or two) or tab. Which characters and the number of spaces needed to constitute a sentence ending is determined by the 'joinspaces' and 'cpoptions' options.

A paragraph begins after each empty line, and also at each of a set of paragraph macros, specified by the pairs of characters in the 'paragraphs' option.

A section begins after a form-feed (<C-L>) in the first column and at each of a set of section macros, specified by the pairs of characters in the 'sections' option.

For more information, read

```
:help sentence
:help 'joinspaces'
:help 'cpoptions' | /^\s*j\>
:help paragraph
:help section
```

```
    :help word
```

10.6. How do I jump to beginning or end of a sentence, paragraph or a section?

You can use the following motion commands to jump to the beginning or end of a sentence or a paragraph or a section:

```
      motion  position    where
      (       beginning   current sentence
      )       end         current sentence
      {       beginning   current paragraph
      }       end         current paragraph
      []      end         previous section
      [[      beginning   current section
      ][      end         current section
      ]]      beginning   next section
```

Each of these motions can be preceded by a number which will extend the jump forward (or backward).

For more information, read

```
    :help object-motions
```

10.7. I have lines in a file that extends beyond the right extent of the screen. How do I move the Vim view to the right to see the text off the screen?

You can use one of the following commands to horizontally scroll the screen to the left or right:

```
    zl - scroll to the left
    zh - scroll to the right
    zL - scroll half a screenwidth to the left
    zH - scroll half a screenwidth to the right
    zs - scroll to position the cursor at the start of the screen
    ze - scroll to position the cursor at the end of the screen
```

You can use the g0 command to move the cursor to the first character of the screen line and the g$ command to move the cursor to the last character of the screen line without scrolling the screen.

For more information, read

```
    :help scroll-horizontal
```

10.8. How do I scroll two or more buffers simultaneously?

You can set the "scrollbind" option for each of the buffer to scroll them simultaneously.

For more information, read

```
    :help 'scrollbind'
```

```
:help scroll-binding
:help 'scrollopt'
```

10.9. When I use my arrow keys, Vim changes modes, inserts weird characters in my document but doesn't move the cursor properly. What's going on?

There are a couple of things that could be going on: either you are using Vim over a slow connection or Vim doesn't understand the key sequence that your keyboard is generating.

If you are working over a slow connection (such as a 2400 bps modem), you can try to set the 'timeout' or 'ttimeout' option. These options, combined with the 'timeoutlen' and 'ttimeoutlen' options, may fix the problem.

The preceding procedure will not work correctly if your terminal sends key codes that Vim does not understand. In this situation, your best option is to map your key sequence to a matching cursor movement command and save these mappings in a file. You can then ":source" the file whenever you work from that terminal.

For more information, read

```
:help 'timeout'
:help 'ttimeout'
:help 'timeoutlen'
:help 'ttimeoutlen'
:help :map
:help vt100-cursor-keys
```

10.10. How do I configure Vim to move the cursor to the end of the previous line, when the left arrow key is pressed and the cursor is currently at the beginning of a line?

You can add the '<' flag to the 'whichwrap' option to configure Vim to move the cursor to the end of the previous line, when the left arrow key is pressed and the cursor is currently at the beginning of a line:

```
:set whichwrap+=<
```

Similarly, to move the cursor the beginning of the next line, when the right arrow key is pressed and the cursor is currently at the end of a line, add the '>' flag to the 'whichwrap' option:

```
:set whichwrap+=>
```

The above will work only in normal and visual modes. To use this in insert and replace modes, add the '[' and ']' flags respectively.

For more information, read

```
:help 'whichwrap'
:help 05.7
```

10.11. How do I configure Vim to stay only in insert mode (modeless editing)?

You can set the 'insertmode' option to configure Vim to stay only in insert mode:

```
:set insertmode
```

By setting this option, you can use Vim as a modeless editor. If you press the <Esc> key, Vim will not go to the normal mode. To execute a single normal mode command, you can press CTRL-O followed by the normal mode command. To execute more than one normal command, you can use CTRL-L followed by the commands. To return to insert mode, press the <Esc> key. To disable this option, reset the 'insertmode' option:

```
:set noinsertmode
```

You can also start vim using the "evim" command or you can use "vim -y" to use Vim as a modeless editor.

You can also start Vim in insert mode using the ":startinsert" ex command.

For more information, read

```
:help 'insertmode'
:help :startinsert
:help :stopinsert
:help i_CTRL-O
:help i_CTRL-L
:help evim
:help evim-keys
```

10.12. How do I display some context lines when scrolling text?

You can set the 'scrolloff' option to display a minimal number of screen lines (context) above and below the cursor.

```
:set scrolloff=10
```

For more information, read

```
:help 'scrolloff'
:help 'sidescrolloff'
```

10.13. How do I go back to previous cursor locations?

You can go back to the cursor location before the latest jump using the '' or `` command. You can use the CTRL-O command to go back to older cursor positions and the CTRL-I command to go to the newer cursor positions in the jump list.

For more information, read

```
:help 03.10
:help mark-motions
:help jump-motions
```

===

SECTION 11 - SEARCHING TEXT

11.1. After I searched for a text with a pattern, all the matched text stays highlighted. How do I turn off the highlighting temporarily/permanently?

The 'hlsearch' option controls whether all the matches for the last searched pattern are highlighted or not. By default, this option is not enabled. If this option is set in a system-wide vimrc file, then you can turn off the search highlighting by using the following command:

```
:set nohlsearch
```

To temporarily turn off the search highlighting, use

```
:nohlsearch
```

You can also clear the search highlighting, by searching for a pattern that is not in the current file (for example, search for the pattern 'asdf').

For more information, read

```
:help 'hlsearch'
:help :nohlsearch
```

11.2. How do I enter a carriage return character in a search pattern?

You can either use '\r' or <CTRL-V><CTRL-M> to enter a carriage return character in a pattern. In Vim scripts, it is better to use '\r' for the carriage return character.

For more information, read

```
:help sub-replace-special
```

11.3. How do I search for the character ^M?

You can enter the ^M character in a search command by first pressing the CTRL-V key and then pressing the CTRL-M key.

```
/^V^M
```

You can also use the "\r" character. In Vim scripts, "\r" is preferred.

For more information, read

```
:help c_CTRL-V
:help using_CTRL-V
:help /\r
```

11.4. How can I search/replace characters that display as '~R', '~S', etc.?

You can use the 'ga' command to display the ASCII value/code for the special character. For example, let us say the ASCII value is 142. Then you can use the following command to search for the special character:

```
/^V142
```

where, ^V is entered by pressing CTRL-V.

For more information, read

```
:help ga
:help using_CTRL_V
:help 24.8
```

11.5. How do I highlight all the non-printable characters in a file?

You can use the following commands and search pattern to highlight all the non-printable characters in a file:

```
:set hlsearch
/\(\p\|$\)\@!.
```

For more information, read

```
:help /\p
:help /bar
:help /$
:help /\(
:help /\@!
:help 'hlsearch'
```

11.6. How do I search for whole words in a file?

You can search for whole words in a file using the \< and \> atoms. For example:

```
/\<myword\>
```

The \< atom matches the beginning of the word and the \> atom matches the end of the word.

For more information, read

```
:help /\<
:help /\>
```

11.7. How do I search for the current word under the cursor?

You can press the * key to search forward for the current word under the cursor. To search backward, you can press the # key. Note that only whole keywords will be searched using these commands.

For more information, read

```
:help star
```

```
:help #
:help g*
:help g#
:help 03.8
:help search-commands
```

11.8. How do I search for a word without regard to the case (uppercase or lowercase)?

To always ignore case while searching for a pattern, set the 'ignorecase' option:

```
:set ignorecase
```

To ignore case only when searching a particular pattern, use the special \c directive:

```
/\c<pattern>
```

For more information, read

```
:help 'ignorecase'
:help /ignorecase
:help /\c
```

11.9. How do I search for words that occur twice consecutively?

You can use one of the following search commands to locate words that occur twice consecutively:

```
 /\(\<\w\+\)\_s\+\1\>
 /\(\<\k\+\)\_s\+\1\>
```

The main difference is the use of '\w' and '\k', where the latter is based on the 'iskeyword' option which may include accented and other language specific characters.

For more information, read

```
:help /\1
:help /\(
:help /\)
:help /\<
:help /\>
:help /\w
:help /\k
:help /\+
:help /\_x
:help 'iskeyword'
```

11.10. How do I count the number of times a particular word occurs in a buffer?

You can use the following set of commands to count the number of times a particular word occurs in a buffer:

```
:let cnt=0
:g/\<your_word\>/let cnt=cnt+1
:echo cnt
```

This only counts the number of lines where the word occurs. You can also use the following command:

```
:%s/\<word\>/&/g
```

To count the number of alphabetic words in a file, you can use

```
:%s/\a\+/&/g
```

To count the number of words made up of non-space characters, you can use

```
:%s/\S\+/&/g
```

For more information, read

```
:help count-items
:help word-count
:help v_g_CTRL-G
:help 12.5
```

11.11. How do I place the cursor at the end of the matched word when searching for a pattern?

You can use the 'e' offset to the search command to place the cursor at the end of the matched word. For example

```
/mypattern/e
```

For more information about search offsets, read

```
:help search-offset
:help /
```

11.12. How do I search for an empty line?

You can search for an empty line using:

```
/^$

or

/^\s*$
```

For more information, read

```
:help /^
:help /$
:help /\s
:help /*
:help search-commands
```

11.13. How do I search for a line containing only a single character?

You can search for a line containing only a single character using:

```
/^\s*\a\s*$
```

For more information, read

```
:help /^
:help /\a
:help /\s
:help /*
:help /$
```

11.14. How do I search and replace a string in multiple files?

You can use the 'argdo' or 'bufdo' or 'windo' commands to execute an ex command on multiple files. For example:

```
:argdo %s/foo/bar/g
```

For more information, read

```
:help :argdo
:help :bufdo
:help :windo
```

11.15. I am using the ":s" substitute command in a mapping. When a search for a pattern fails, the map terminates. I would like the map to continue processing the next command, even if the substitute command fails. How do I do this?

You can use the 'e' flag to the substitute command to continue processing other commands in a map, when a pattern is not found.

For more information, read

```
:help :s_flags
```

11.16. How do I search for the n-th occurrence of a character in a line?

To search for the n-th occurrence of a character in a line, you can prefix the 'f' command with a number. For example, to search for the 5th occurrence of the character @ in a line, you can use the command 5f@. This assumes the cursor is at the beginning of the line - and that this first character is not the one your are looking for.

For more information, read

```
:help f
:help F
:help t
:help T
:help ;
```

```
    :help ,
```

11.17. How do I replace a tab (or any other character) with a hard return (newline) character?

You can replace a tab (or any other character) with a hard return (newline) character using the following command:

```
    :s/\t/\r/
```

Note that in the above command, if you use \n instead of \r, then the tab characters will not be replaced by a new-line character.

For more information, read

```
    :help sub-replace-special
    :help NL-used-for-Nul
    :help CR-used-for-NL
```

11.18. How do I search for a character by its ASCII value?

You can search for a character by its ASCII value by pressing CTRL-V followed by the decimal or hexadecimal or octal value of that character in the search "/" command. To determine the ASCII value of a character you can use the ":ascii" or the "ga" command.

For more information, read

```
    :help i_CTRL-V_digit
    :help :ascii
    :help ga
```

11.19. How do I search for long lines?

You can search for long lines or lines containing more than a specific number of characters using the Vim regular-expressions in the search command. For example, to search for all the lines containing more than 80 characters, you can use one of the following commands:

```
    /^.\{80}.*$
    /^.*\%80c.*$
```

For more information, read

```
    :help /\{
    :help /\%c
```

11.20. How do I display all the lines in the current buffer that contain a specified pattern?

You can use the following command to display all the lines in the current buffer that contain a specified pattern:

```
    :g/<pattern>/p
```

For example, the following command will display all the lines in the current buffer that contain "vim":

```
:g/vim/p
```

If you also want the corresponding line numbers, then you can use the following command:

```
:g/<pattern>/#
```

For more information, read

```
:help :global
:help :print
:help :number
```

11.21. How do I search for a text string that spans multiple lines?

You can search for a text string that spans multiple lines using the _x regular expression atom. For example, to search for the text string "Hello World", you can use the following search command:

```
/Hello\_sWorld
```

This will match the word "Hello" followed by a newline character and then the word "World" at the beginning of the next line. This will also match the word "Hello" immediately followed by a space character and then the word "World". When searching for the "Hello World" string, to include the space characters at the end and beginning of the line, you can use the following search command:

```
/Hello\_s\+World
```

For more information, read

```
:help 27.8
:help pattern-atoms
:help /\_
:help pattern-searches
```

11.22. How do I search for a pattern within the specified range of lines in a buffer?

You can search for a pattern within a range of lines using the \%>l and \%<l regular expression atoms.

For example, to search for the word 'white' between the lines 10 and 30 in a buffer, you can use the following command:

```
/white\%>10l\%<20l
```

For more information, read

```
:help /\%l
```

===

SECTION 12 - CHANGING TEXT

12.1. How do I delete all the trailing white space characters (SPACE and TAB) at the end of all the lines in a file?

You can use the ":substitute" command on the entire file to search and remove all the trailing white space characters:

```
:%s/\s\+$//
```

For more information, read

```
:help :%
:help :s
:help /\s
:help /\+
:help /$
```

12.2. How do I replace all the occurrences of multiple consecutive space characters to a single space?

You can use the following command to replace all the occurrences of multiple consecutive space characters to a single space:

```
:%s/ \{2,}/ /g
```

For more information, read

```
:help :%
:help :s
:help /\{
:help :s_flags
```

12.3. How do I reduce a range of empty lines into one line only?

You can use the following command to reduce a range of empty lines into one line only:

```
:v/./.,/./-1join
```

The explanation for this command is below:

```
:v/./           Execute the following command for all lines not
                containing a character (empty lines).
     .,         Use the current line as the start of the range of
                lines.
       /./      Use the line containing a character as the last line.
          -1    Adjust the range of lines to end with the line before
                the last line.
            j   Join the lines in the range.
```

Note that this will give an error message if the empty lines are at the end

of the file. To correct this, you have to add a temporary line at the end of the file, execute the command and then remove the temporary line.

For more information, read

```
:help :v
:help :join
:help cmdline-ranges
:help collapse
```

12.4. How do I delete all blank lines in a file? How do I remove all the lines containing only space characters?

To remove all blank lines, use the following command:

```
:g/^$/d
```

To remove all lines with only whitespace (spaces or tabs) in them, use the following command:

```
:g/^\s\+$/d
```

To remove all the lines with only whitespace, if anything, use the following command:

```
:g/^\s*$/d
```

12.5. How do I copy/yank the current word?

You can use the "yiw" (yank inner word without whitespace) command or the "yaw" (yank a word with whitespace) command to copy/yank the current word.

For more information, read

```
:help 04.6
:help 04.8
:help iw
:help yank
:help text-objects
:help objects
```

12.6. How do I yank text from one position to another position within a line, without yanking the entire line?

You can specify a motion command with the yank operator (y) to yank text from one position to another position within a line. For example, to yank from the current cursor position till the next letter x, use yfx or Fx or tx or Tx. To yank till the nth column, use n|. To yank till the next occurrence of a 'word', use /word. To do a yank till the nth column on another line, first mark the position using the 'ma' command, go to the start of the yank position, and then yank till the mark using y`a (note the direction of the quote)

For more information, read

```
    :help yank
    :help motion.txt
    :help 4.6
```

12.7. When I yank some text into a register, how do I append the text to the current contents of the register?

When you specify the register for some operation, if you use the upper-case for the register name, then the new text will be appended to the existing contents. For example, if you have some text in the register "a". If you want to append some new text to this, you have to use the "A" register name. If you use the lowercase register name, then the contents of the register will be overwritten with the new text.

For more information, read

```
    :help quote
    :help quote_alpha
    :help 10.1
```

12.8. How do I yank a complete sentence that spans over more than one line?

To yank a complete sentence that spans over more than one line you have to use the yank operator followed by a motion command. For example:

```
        y)
```

From inside the sentence you can use 'yi)' to yank the sentence.

For more information, read

```
    :help yank
    :help {motion}
    :help object-motions
    :help 4.6
```

12.9. How do I yank all the lines containing a pattern into a buffer?

You can use the ":global" command to yank all the lines containing the pattern into a register and then paste the contents of the register into the buffer:

```
    :let @a=''
    :g/mypattern/y A
```

The first command, clears the contents of the register "a". The second command copies all the lines containing "mypattern" into the register "a". Note that the capital letter "A" is used to append the matched lines. Now you can paste the contents of register "a" to a buffer using "ap command. For more information, read

```
    :help :g
    :help :y
    :help let-register
```

```
:help quote_alpha
:help put
:help registers
:help :registers
```

12.10. How do I delete all the lines in a file that does not contain a pattern?

You can use ":v" command to delete all the lines that does not contain a pattern:

```
:v/pattern/d
```

or

```
:g!/pattern/d
```

For more information, read

```
:help :v
:help :g
```

12.11. How do I add a line before each line with "pattern" in it?

You can use the following command to add a line before each line with "pattern" in it:

```
:g/pattern/normal Oi<line of text goes here>
```

Alternatively you can yank the line using the Y command and then insert the line using the following command:

```
:g/pattern/put!
```

For more information, read

```
:help :g
:help :put
:help insert
:help 0
```

12.12. Is there a way to operate on a line if the previous line contains a particular pattern?

You can use the ":global" command to operate on a line, if the previous line contains a particular pattern:

```
:g/<pattern>/+{cmd}
```

For more information, read

```
:help :g
:help :range
```

12.13. How do I execute a command on all the lines containing a pattern?

You can use the ":global" (:g) command to execute a command on all the lines containing a pattern.

```
:g/my pattern/d
```

If you want to use a non-Ex command, then you can use the ":normal" command:

```
:g/my pattern/normal {command}
```

Unless you want the normal mode commands to be remapped, consider using a ":normal!" command instead (note the "!").

For more information, read

```
:help :global
:help :v
:help :normal
```

12.14. Can I copy the character above the cursor to the current cursor position?

In Insert mode, you can copy the character above the cursor to the current cursor position by typing <Ctrl-Y>. The same can be done with the characters below the cursor by typing <Ctrl-E>.

For more information, read

```
:help i_CTRL-Y
:help i_CTRL-E
```

12.15. How do I insert a blank line above/below the current line without entering insert mode?

You can use the ":put" ex command to insert blank lines. For example, try

```
:put =''
:put! =''
```

For more information, read

```
:help :put
```

12.16. How do I insert the name of current file into the current buffer?

There are several ways to insert the name of the current file into the current buffer. In insert mode, you can use the <C-R>% or the <C-R>=expand("%") command. In normal mode, you can use the ":put =@%" command.

For more information, read

```
:help i_CTRL-R
```

```
    :help expand()
    :help !!
```

12.17. How do I insert the contents of a Vim register into the current buffer?

In insert mode, you can use the <C-R><register> command to insert the contents of <register>. For example, use <C-R>a to insert the contents of register "a" into the current buffer.

In normal mode, you can use the ":put <register>" command to insert the contents of <register>. For example, use the ":put d" command to insert the contents of register "d" into the current buffer.

For more information, read

```
    :help i_CTRL-R
    :help :put
```

12.18. How do I move the cursor past the end of line and insert some characters at some columns after the end of the line?

You can set the "virtualedit" option to move the cursor past the end-of-line and insert characters in a column after the end-of-line. To start the virtual mode, use

```
    :set virtualedit=all
```

For more information, read

```
    :help 'virtualedit'
```

12.19. How to replace the word under the cursor (say: junk) with "foojunkbar" in Vim?

There are several ways to do this. If the word is the first such word on the line, use the following command:

```
    :exe "s/".expand("<cword>")."/foo&bar/"
```

Too match specifically you could use a more complex substitution like this:

```
    :exe 's/\<'.expand("<cword>").'\%>'.(col(".")-1).'c\>/foo&bar/'
```

You can also use the command: ciwfoo<C-R>"bar<Esc>

For more information, read

```
    :help :substitute
    :help expand()
    :help col()
    :help /\%c
```

12.20. How do I replace a particular text in all the files in a directory?

You can use the "argdo" command to execute the substitute command on all the files specified as arguments:

```
:args *
:argdo %s/<your_text>/<replacement_text>/ge | update
```

For more information, read

```
:help :args_f
:help :argdo
:help :s_flags
```

12.21. I have some numbers in a file. How do I increment or decrement the numbers in the file?

You can use the CTRL-A key to increment the number and the CTRL-X key to decrement the number. You can also specify the number to increment/decrement from the number by specifying a count to the key. This works for decimal, octal and hexadecimal numbers. You can change the base used by Vim for this operation by modifying the 'nrformats' option.

For more information, read

```
:help 26.2
:help CTRL-A
:help CTRL-X
:help 'nrformats'
```

12.22. How do I reuse the last used search pattern in a ":substitute" command?

To reuse the last used search pattern in a ":substitute" command, don't specify a new search pattern:

```
:s/pattern/newtext/
:s//sometext/
```

In the second ":s" command, as a search pattern is not specified, the pattern specified in the first ":s" command 'pattern' will be used.

If you want to change the search pattern but repeat the substitution pattern you can use the special right hand side, you can use the tilde character:

```
:s/newpattern/~/
```

For more information, read

```
:help :s
:help :&
:help :~
:help &
:help sub-replace-special
```

12.23. How do I change the case of a string using the ":substitute" command?

You can use special characters in the replacement string for a ":substitute" command to change the case of the matched string. For example, to change the case of the string "MyString" to all uppercase, you can use the following command:

```
:%s/MyString/\U&/g
```

To change the case to lowercase, you can use the following command:

```
:%s/MyString/\L&/g
```

To change the case of the first character in all the words in the current line to uppercase, you can use the following command:

```
:s/\<\(.\)\(\k*\)\>/\u\1\L\2/g
```

For more information, read

```
:help sub-replace-special
:help :substitute
:help \U
:help \L
:help \u
```

12.24. How do I enter characters that are not present in the keyboard?

You can use digraphs to enter characters that are not present in the keyboard. You can use the ":digraphs" command to display all the currently defined digraphs. You can add a new digraph to the list using the ":digraphs" command.

For more information, read

```
:help digraphs
:help 'digraphs'
:help 24.9
```

12.25. Is there a command to remove any or all digraphs?

No. The digraphs table is defined at compile time. You can only add new ones. Adding a command to remove digraphs is on the todo list.

12.26. In insert mode, when I press the backspace key, it erases only the characters entered in this instance of insert mode. How do I erase previously entered characters in insert mode using the backspace key?

You can set the 'backspace' option to erase previously entered characters in insert mode:

```
:set backspace=indent,eol,start
```

For more information, read

```
:help 'backspace'
:help i_backspacing
```

12.27. I have a file which has lines longer than 72 characters terminated with "+" and wrapped to the next line. How can I quickly join the lines?

You can use the ":global" command to search and join the lines:

```
:g/+$/j
```

This will, however, only join every second line. A couple of more complex examples which will join all consecutive lines with a "+" at the end are:

```
:g/*$/,/\(^\|[^+]\)$/j
:g/+$/mark a | .,/\(^\|[^+]\)$/s/+$// | 'a,.j
```

For more information, read

```
:help :g
:help :j
:help :mark
```

12.28. How do I paste characterwise yanked text into separate lines?

You can use the ":put" command to paste characterwise yanked text into new lines:

```
:put =@"
```

For more information, read

```
:help :put
:help quote_=
```

12.29. How do I change the case (uppercase, lowercase) of a word or a character or a block of text?

You can use the "~" command to switch the case of a character.

You can change the case of the word under the cursor to uppercase using the "gUiw" or "viwU" command and to lowercase using the "guiw" or "viwu" command.

You can switch the case (upper case to lower case and vice versa) of the word under the cursor using the "viw~" or "g~iw" command.

You can use the "gUgU" command to change the current line to uppercase and the "gugu" command to change the current line to lowercase.

You can use the "g~g~" command to switch the case of the current line. You can use the "g~{motion}" or "{Visual}~" commands to switch the case of a block of text.

For more information, read

```
:help case
```

12.30. How do I enter ASCII characters that are not present in the keyboard?

You can enter ASCII characters that are not present in the keyboard by pressing CTRL-V and then the ASCII character number. You can also use digraphs to enter special ASCII characters.

For more information, read

```
:help i_CTRL-V_digit
:help digraphs
:help 45.5
```

12.31. How do I replace non-printable characters in a file?

To replace a non-printable character, you have to first determine the ASCII value for the character. You can use the ":ascii" ex command or the "ga" normal-mode command to display the ASCII value of the character under the cursor.

You can enter the non-printable character by entering CTRL-V followed by the decimal number 1-255 (with no leading zero), or by x and a hex number 00-FF, or by an octal number 0-0377 (with leading zero), or by u and a hex number 0-FFFF, or by U and a hex number 0-7FFFFFFF

Another alternative is to use the ":digraphs" ex command to display the digraphs for all characters, together with their value in decimal and alpha. You can enter a non-printable character by entering CTRL-K followed by two alphanumeric characters (a digraph).

For more information, read

```
:help :ascii
:help i_CTRL-V
:help i_CTRL-V_digit
:help :digraphs
```

12.32. How do I remove duplicate lines from a buffer?

You can use the following user-defined command to remove all the duplicate lines from a buffer:

```
:command -range=% Uniq <line1>,<line2>g/^\%<<line2>l\(.*\)\n\1$/d
```

Add the above command to your .vimrc file and invoke ":Uniq" to remove all the duplicate lines.

12.33. How do I prefix all the lines in a file with the corresponding line numbers?

You can prefix the lines with the corresponding line number in several ways. Some of them are listed below:

```
:%s/^/\=line('.'). ' '
:%s/^/\=strpart(line(".")."     ", 0, 5)
:g/^/exec "s/^/".strpart(line(".")."    ", 0, 4)
```

For more information, read

```
:help sub-replace-special
:help line()
:help expr6
:help strpart()
:help :execute
:help :global
```

12.34. How do I exchange (swap) two characters or words or lines?

You can exchange two characters with the "xp" command sequence. The 'x' will delete the character under the cursor and 'p' will paste the just deleted character after the character under the cursor. This will result in exchanging the two characters.

You can exchange two words with the "deep" command sequence (start with the cursor in the blank space before the first word).

You can exchange two lines with the "ddp" command sequence. The 'dd' will delete the current line and 'p' will paste the just deleted line after the current line. This will result in exchanging the two lines.

All of the above operations will change the " unnamed register.

You can use the ":m +" ex command to exchange two lines without changing the unnamed register.

For more information, read

```
:help x
:help p
:help dd
:help d
:help e
:help linewise-register
:help quotequote
:help :move
```

12.35. How do I change the characters used as word delimiters?

Vim uses the characters specified by the 'iskeyword' option as word delimitiers. The default setting for this option is "@,48-57,_,192-255".

For example, to add ':' as a word delimitier, you can use

```
:set iskeyword+=:
```

To remove '_' as a word delimitier, you can use

```
:set iskeyword-=_
```

For more information, read

```
:help 'iskeyword'
:help word
```

===

SECTION 13 - COMPLETION IN INSERT MODE

13.1. How do I complete words or lines in insert mode?

In insert mode, you can complete words using the CTRL-P and CTRL-N keys. The CTRL-N command searches forward for the next matching keyword. The CTRL-P command searches backwards for the next matching keyword.

In insert mode, you can use the CTRL-X CTRL-L command sequence to complete lines that starts with the same characters as in the current line before the cursor. To get the next matching line, press the CTRL-P or CTRL-N keys. There are a lot of other keys/ways available to complete words in insert mode.

Vim supports completion of the following items:

```
CTRL-X CTRL-F    file names
CTRL-X CTRL-L    whole lines
CTRL-X CTRL-D    macro definitions (also in included files)
CTRL-X CTRL-I    current and included files
CTRL-X CTRL-K    words from a dictionary
CTRL-X CTRL-T    words from a thesaurus
CTRL-X CTRL-]    tags
CTRL-X CTRL-V    Vim command line
```

For more information, read

```
:help 24.3
:help ins-completion
```

13.2. How do I complete file names in insert mode?

In insert mode, you can use the CTRL-X CTRL-F command sequence to complete filenames that start with the same characters as in the current line before the cursor.

For more information, read

```
:help compl-filename
```

13.3. I am using CTRL-P/CTRL-N to complete words in insert mode. How do I complete words that occur after the just completed word?

You can use CTRL-X CTRL-N and CTRL-X CTRL-P keys to complete words that are present after the just completed word.

For more information, read

```
    :help i_CTRL-X_CTRL-P
    :help i_CTRL-X_CTRL-N
    :help ins-completion
```

===

SECTION 14 - TEXT FORMATTING

14.1. How do I format a text paragraph so that a new line is inserted at the end of each wrapped line?

You can use the 'gq' command to format a paragraph. This will format the text according to the current 'textwidth' setting.

Note that the gq operator can be used with a motion command to operate on a range of text. For example:

```
    gqgq - Format the current line
    gqap - Format current paragraph
    gq3j - Format the current and the next 3 lines
```

For more information, read

```
    :help gq
    :help formatting
    :help usr_25.txt
    :help motion.txt
```

14.2. How do I format long lines in a file so that each line contains less than 'n' characters?

You can set the 'textwidth' option to control the number of characters that can be present in a line. For example, to set the maximum width of a line to 70 characters, you can use the following command:

```
    set textwidth=70
```

Now to break the long lines in a file to the length defined by the 'textwidth' option, you can use

```
    :g/./normal gqq
```

For more information, read

```
    :help 'textwidth'
    :help gq
```

14.3. How do I join short lines to form a paragraph?

First, make sure the 'textwidth' option is set to a high value:

```
:set textwidth=99999
```

Next, join the short lines to form a paragraph using the command:

```
1GgqG
```

The above command will operate on the entire file. To do the formatting on all paragraphs in a specific range, use:

```
:'a,'bg/\S/normal gq}
```

For more information, read

```
:help gq
:help G
:help gqq
```

14.4. How do I format bulleted and numbered lists?

You can configure Vim to format bulleted and numbered lists using the 'formatoptions' option. For example, you can format the list of the following format:

```
  - this is a test. this is a test. this is a test. this is a test.
  this is a test.
```

into this format:

```
  - this is a test. this is a test. this is a test. this is a test.
    this is a test.
```

You can use the 'n' flag in the 'formatoptions' to align the text.

```
:set fo+=n
```

With this option, when formatting text, Vim will recognize numbered lists. For this option to work, the 'autoindent' option also must be set.

For more information, read

```
:help 'formatoptions'
:help fo-table
:help format-comments
```

14.5. How do I indent lines in insert mode?

In insert mode, you can press the CTRL-T key to insert one shiftwidth of indent at the start of the current line. In insert mode, you can use the CTRL-D key to delete on shiftwidth of indent at the start of the current line. You can also use the CTRL-O >> and CTRL-O << commands to indent the current line in insert mode.

For more information, read

```
:help i_CTRL-T
:help i_CTRL-D
:help i_0_CTRL-D
:help i_CTRL-O
:help >>
:help <<
```

14.6. How do I format/indent an entire file?

You can format/indent an entire file using the gg=G command, where

```
gg - Goto the beginning of the file
=  - apply indentation
G  - till end of file
```

For more information, read

```
:help gg
:help =
:help G
:help 'formatprg'
:help C-indenting
```

14.7. How do I increase or decrease the indentation of the current line?

You can use the '>>' and '<<' commands to increase or decrease the indentation of the current line.

For more information, read

```
:help shift-left-right
:help >>
:help <<
:help 'shiftwidth'
```

14.8. How do I indent a block/group of lines?

You can visually select the group of lines and press the > or < key to indent/unindent the lines. You can also use the following ex-command to indent the lines

```
:10,20>
```

For more information, read

```
:help shift-left-right
:help v_>
:help v_<
:help :<
:help :>
```

14.9. When I indent lines using the > or < key, the standard 8-tabstops are used instead of the current 'tabstop' setting. Why?

The number of spaces used when lines are indented using the ">" operator is controlled by the 'shiftwidth' option. The 'tabstop' setting is not used for indentation. To change the amount of spaces used for indentation, use the command:

```
:set shiftwidth=4
```

For more information, read

```
:help 'shiftwidth'
:help >>
:help 'softtabstop'
```

14.10. How do I turn off the automatic indentation of text?

By default, the automatic indentation of text is not turned on. Check the configuration files (.vimrc, .gvimrc) for settings related to indentation. Make sure the ":filetype indent on" command is not present. If it is present, remove it. Also, depending on your preference, you may also want to check the value of the 'autoindent', 'smartindent', 'cindent' and 'indentexpr' options and turn them off as needed.

For more information, read

```
:help :filetype-indent-off
:help 'autoindent'
:help 'smartindent'
:help 'cindent'
:help 'indentexpr'
```

14.11. How do I configure Vim to automatically set the 'textwidth' option to a particular value when I edit mails?

You can use the 'FileType' autocommand to set the 'textwidth' option:

```
autocmd FileType mail set tw=<your_value>
```

For more information, read

```
:help :autocmd
:help FileType
:help usr_43.txt
```

14.12. Is there a way to make Vim auto-magically break lines?

Yes. Set the 'textwidth' option to the preferred length for a line. Then Vim will auto-magically break the newly entered lines. For example:

```
:set textwidth=75
```

For more information, read

```
:help 'textwidth'
:help ins-textwidth
:help 'formatoptions'
```

```
    :help fo-table
    :help formatting
```

14.13. I am seeing a lot of ^M symbols in my file. I tried setting the 'fileformat' option to 'dos' and then 'unix' and then 'mac'. None of these helped. How can I hide these symbols?

When a file is loaded in Vim, the format of the file is determined as below:

- If all the lines end with a new line (<NL>), then the fileformat is 'unix'.
- If all the lines end with a carriage return (<CR>) followed by a new line (<NL>), then the fileformat is 'dos'.
- If all the lines end with carriage return (<CR>), then the fileformat is 'mac'.

If the file has some lines ending with <CR> and some lines ending with <CR> followed by a <NL>, then the fileformat is set to 'unix'.

You can change the format of the current file, by modifying the 'fileformat' option and then saving the file:

```
    :set fileformat=dos
    :w
```

To display the format of the current file, use

```
    :set fileformat?
```

The above behavior is also controlled by the 'fileformats' option. You can try the following commands:

```
    :set fileformats+=unix
    :e <your_file>
    :set fileformat=unix
    :w
```

To remove the carriage return (<CR>) character at the end of all the lines in the current file, you can use the following command:

```
    :%s/\r$//
```

To force Vim to use a particular file format, when editing a file, you can use the following command:

```
    :e ++ff=dos filename
```

For more information, read

```
    :help 'fileformats'
    :help 'fileformat'
    :help file-formats
    :help DOS-format-write
    :help Unix-format-write
    :help Mac-format-write
    :help dos-file-formats
```

```
:help 23.1
:help ++ff
```

14.14. When I paste some text into a Vim buffer from another application, the alignment (indentation) of the new text is messed up. How do I fix this?

The indentation of the text is messed up due to various Vim settings related to indentation (like autoindent, smartindent, textwidth etc). Before pasting text into Vim, you can set the 'paste' option:

```
:set paste
```

After pasting the text, you can turn off the option using:

```
:set nopaste
```

You can also toggle the paste option using:

```
:set paste!
```

If you can access the clipboard through the * register, then you can paste the text without indentation using CTRL-R CTRL-O *.

For more information, read

```
:help 'paste'
:help 'pastetoggle'
:help i_CTRL-R_CTRL_O
:help clipboard
:help xterm-clipboard
:help gui-clipboard
```

14.15. When there is a very long wrapped line (wrap is "on") and a line doesn't fit entirely on the screen it is not displayed at all. There are blank lines beginning with '@' symbol instead of wrapped line. If I scroll the screen to fit the line the '@' symbols disappear and the line is displayed again. What Vim setting control this behavior?

You can set the 'display' option to 'lastline' to display as much as possible of the last line in a window instead of displaying the '@' symbols.

```
:set display=lastline
```

For more information, read

```
:help 'display'
```

14.16. How do I convert all the tab characters in a file to space characters?

You can use the ":retab" command to update all the tab characters in the current file with the current setting of 'expandtab' and 'tabstop'. For example, to convert all the tabs to white spaces, use

```
    :set expandtab
    :retab
```

For more information, read

```
    :help :retab
    :help 'expandtab'
    :help 'tabstop'
    :help 25.3
```

14.17. What Vim options can I use to edit text that will later go to a word processor?

You can set the following options to edit text that will later go into a word processor:

```
    :set wrap
    :set linebreak
    :set textwidth=0
    :set showbreak=>>>
```

You can use the 'gk' and 'gj' commands to move one screen line up and down. For more information, read

```
    :help 'wrap'
    :help 'linebreak'
    :help 'textwidth'
    :help 'showbreak'
    :help gk
    :help gj
```

14.18. How do I join lines without adding or removing any space characters?

By default, when you join lines using the "J" or ":join" command, Vim will replace the line break, leading white space and trailing white space with a single space character. If there are space characters at the end of a line or a line starts with the ')' character, then Vim will not add a space character.

To join lines without adding or removing any space characters, you can use the gJ or ":join!" commands.

For more information, read

```
    :help gJ
    :help :join
    :help J
    :hep 10.5
    :help 'joinspaces'
    :help 'cpoptions'
    :help 'formatoptions'
```

===

SECTION 15 - VISUAL MODE

15.1. How do I do rectangular block copying?

You can do rectangular block copying in Vim using the blockwise visual mode. To start blockwise visual mode use the CTRL-V key. Move the cursor using any of the motion commands and then use the y operator to yank to visually selected text.

If CTRL-V does not work as expected, it may have been remapped to CTRL-Q by the mswin.vim script which is often sourced by a vimrc on Windows machines to mimic some common short cuts from other programs.

For more information, read

```
:help 04.4
:help blockwise-visual
:help visual-mode
:help Q_vi
```

15.2. How do I delete or change a column of text in a file?

You can use the Vim block-wise visual mode to select the column of text and apply an operator (delete, change, copy, etc) on it.

For more information, read

```
:help visual-block
:help visual-operators
```

15.3. How do I apply an ex-command on a set of visually selected lines?

When you select a range of lines in visual mode, the < register is set to the start of the visual region and the > register is set to the end of the visual region. You can use these registers to specify the range for an ex command. After visually selecting the lines, press ":" to go to the command mode. Vim will automatically insert the visual range '<,'>. You can run any ex-command on the visual range.

For more information, read

```
:help v_:
:help '<
:help '>
```

15.4. How do I execute an ex command on a column of text selected in Visual block mode?

All the ex commands operate on whole lines only. If you try to execute an ex command on a column of text selected in visual block mode, Vim will operate on all the selected lines (instead of the selected columns). You can use the vis.vim plugin script from http://vim.sourceforge.net scripts archive to do this.

For more information, read

```
:help cmdline-ranges
:help 10.3
:help cmdline-lines
```

15.5. How do I select the entire file in visual mode?

You can select the entire file in visual mode using ggVG.

```
gg - go to the beginning of the file.
V  - Start linewise visual mode
G  - goto the end of the file.
```

For more information, read

```
:help gg
:help linewise-visual
:help G
```

15.6. When I visually select a set of lines and press the > key to indent the selected lines, the visual mode ends. How can I reselect the region for further operation? (or) How do I re-select the last selected visual area again?

You can use the 'gv' command to reselect the last selected visual area. You can also use the marks '< and '> to jump to the beginning or the end of the last selected visual area.

For more information, read

```
:help gv
:help '<
:help '>
```

15.7. How do I jump to the beginning/end of a visually selected region?

You can use the 'o' command to jump to the beginning/end of a visually selected region.

For more information, read

```
:help v_o
```

15.8. When I select text with mouse and then press : to enter an ex command, the selected text is replaced with the : character. How do I execute an ex command on a text selected using the mouse similar to the text selected using the visual mode?

This will happen if you have configured Vim to use select mode instead of Visual mode by setting the 'selectmode' option. Check the value of this option:

```
:set selectmode?
```

This mode is known as selectmode and is similar to the visual mode. This option is also automatically set when you use the "behave mswin" command. Select mode looks like visual mode, but it is similar to the selection mode in MS-Windows.

For more information, read

```
:help Select-mode
:help 'selectmode'
:help 9.4
:help :behave
```

15.9. When I select a block of text using the mouse, Vim goes into selection mode instead of Visual mode. Why?

The 'selectmode' option controls whether Select mode will be started when selecting a block of text using the mouse. To start Visual mode when selecting text using mouse, remove the 'mouse' value from the 'selectmode' option:

```
:set selectmode-=mouse
```

Note that by default, the 'selectmode' option will be set to empty, so that always visual mode is used.

For more information, read

```
:help 'selectmode'
:help Select-mode
:help :behave
```

15.10. How do I visually select the last copy/pasted text?

You can use the '[and '] marks to visually select the last copy/pasted text. The '[mark is set to the beginning of the last changed/yanked text and the '] mark is set to the end of the last changed/yanked text. To visually select this block of text use the command '[v']

For more information, read

```
:help '[
:help ']
:help `a
:help v
```

===

SECTION 16 - COMMAND-LINE MODE

16.1. How do I use the name of the current file in the command mode or an ex command line?

In the command line, the '%' character represents the name of the current

file. In some commands, you have to use expand("%") to get the filename:

```
:!perl %
```

For more information, read

```
:help :_%
:help cmdline-special
:help expand()
```

16.2. How do I edit the text in the Vim command-line effectively?

You can use the command-line window for editing Vim command-line text. To open the Vim command-line window use the "q:" command in normal mode. In command-line mode, use the CTRL-F key. In this window, the command line history will be displayed. You can use normal Vim keys/commands to edit any previous/new command line. To execute a command line, press the enter/return key.

In a similar vain, the search history can be edited with "q/" and "q?" commands.

For more information, read

```
:help cmdline-window
```

16.3. How do I switch from Vi mode to Ex mode?

You can use the Q command to switch from Vi mode to Ex mode. To switch from Ex mode back to the Vi mode, use the :vi command.

For more information, read

```
:help Q
:help gQ
:help Ex-mode
:help :vi
```

16.4. How do I copy the output from an ex-command into a buffer?

To copy the output from an ex-command into a buffer, you have to first get the command output into a register. You can use the ":redir" command to get the output into a register. For example,

```
:redir @a
:g/HelloWord/p
:redir END
```

Now the register 'a' will contain the output from the ex command "g/HelloWord/p". Now you can paste the contents of the register 'a' into a buffer. You can also send or append the output of an ex-command into a file using the 'redir' command.

You can prefix the ":global" command with ":silent", to avoid having the lines printed to the screen.

To redirect the output from an ex-command to a file, you can use the following set of commands:

```
:redir > myfile
:g/HelloWord/p
:redir END
```

For more information, read

```
:help :redir
:help :silent
```

16.5. When I press the tab key to complete the name of a file in the command mode, if there are more than one matching file names, then Vim completes the first matching file name and displays a list of all matching filenames. How do I configure Vim to only display the list of all the matching filenames and not complete the first one?

You can modify the 'wildmode' option to configure the way Vim completes filenames in the command mode. In this case, you can set the 'wildmode' option to 'list':

```
:set wildmode=list
```

For more information, read

```
:help 'wildmode'
```

16.6. How do I copy text from a buffer to the command line and from the command line to a buffer?

To copy text from a buffer to the command line, after yanking the text from the buffer, use Ctrl-R 0 in the command line to paste the text. You can also yank the text to a specific register and use CTRL-R <register> to paste the text to the command line. You can use CTRL-R CTRL-W to paste the word under the cursor in the command line.

To copy text from the command line into a buffer, you can paste the contents of the : register using the ":p command. The most recently executed command line is stored in the : register.

Another approach for copying and pasting text to and from the command line is to open the command line window using q: from normal mode or CTRL-F from the command-line mode. In the command line window you can use all the Vim commands to edit the command line.

For more information, read

```
:help c_CTRL-R
:help quote_:
:help cmdline-window
```

16.7. How do I put a command onto the command history without executing it?

To put a command onto the command history without executing it, press the <Esc> key to cancel the command.

For more information, read

```
:help c_<Esc>
```

16.8. How do I increase the height of the command-line?

You can increase the height of the command-line by changing the 'cmdheight' option:

```
:set cmdheight=2
```

For more information, read

```
:help 'cmdheight'
:help hit-enter
:help 05.7
```

===

SECTION 17 - VIMINFO

17.1. When I invoke Vim, I get error messages about illegal characters in the viminfo file. What should I do to get rid of these messages?

You can remove the $HOME/.viminfo or the $HOME/_viminfo file to get rid of these error messages.

For more information, read

```
:help viminfo-errors
:help viminfo-file-name
:help viminfo
:help 21.3
```

17.2. How do I disable the viminfo feature?

By default, the viminfo feature is disabled. If the viminfo feature is enabled by a system-wide vimrc file, then you can disable the viminfo feature by setting the 'viminfo' option to an empty string in your local .vimrc file:

```
:set viminfo=""
```

For more information, read

```
:help 'viminfo'
```

17.3. How do I save and use Vim marks across Vim sessions?

You can save and restore Vim marks across Vim sessions using the viminfo

file. To use the viminfo file, make sure the 'viminfo' option is not empty. To save and restore Vim marks, the 'viminfo' option should not contain the 'f' flag or should have a value greater than zero for the 'f' option.

For more information, read

```
:help 21.3
:help viminfo
:help 'viminfo'
:help :wviminfo
:help :rviminfo
```

===

SECTION 18 - REMOTE EDITING

18.1. How do I open a file with existing instance of gvim? What happened to the Vim 5.x OpenWithVim.exe and SendToVim.exe files?

Starting with Vim6, the OLE version of OpenWithVim.exe and SendToVim.exe Vim utilities are replaced by the new client-server feature. To open the file j.txt with an existing instance of Gvim (MyVim), use:

```
$ gvim --servername MyVim --remote-silent j.txt
```

To list the server names of all the currently running Vim instances, use

```
$ vim --serverlist
```

To get more information about client-server feature, read

```
:help client-server
```

18.2. How do I send a command to a Vim server to write all buffers to disk?

You can use the Vim remote server functionality to do this:

```
$ gvim --servername myVIM --remote-send "<C-\><C-N>:wall<CR>"
```

For more information, read

```
:help client-server
:help CTRL-\_CTRL-N
:help :wall
```

18.3. Where can I get the documentation about the Vim remote server functionality?

You can get more information about the Vim remote server functionality by reading

```
:help client-server
```

===

SECTION 19 - OPTIONS

19.1. How do I configure Vim in a simple way?

You can use the ":options" command to open the Vim option window:

```
:options
```

This window can be used for viewing and setting all the options.

For more information, read

```
:help :options
```

19.2. How do I toggle the value of an option?

You can prefix the option with "inv" to toggle the value of the option:

```
:set invignorecase
:set invhlsearch
```

You can also suffix the option with "!" to toggle the value:

```
:set ignorecase!
:set hlsearch!
```

For more information, read

```
:help set-option
```

19.3. How do I set an option that affects only the current buffer/window?

Some of the Vim options can have a local or global value. A local value applies only to a specific buffer or window. A global value applies to all the buffers or windows.

When a Vim option is modified using the ":set" command, both the global and local values for the option are changed. You can use the ":setlocal" command to modify only the local value for the option and the ":setglobal" command to modify only the global value.

You can use the ":setlocal" command to set an option that will affect only the current file/buffer:

```
:setlocal textwidth=70
```

Note that not all options can have a local value. You can use ":setlocal" command to set an option locally to a buffer/window only if the option is allowed to have a local value.

You can also use the following command to set a option locally:

```
:let &l:{option-name} = <value>
```

For more information, read

```
:help :setlocal
:help local-options
```

19.4. How do I use space characters for a Vim option value?

To use space characters in a Vim option value, you have to escape the space character. For example:

```
:set tags=tags\ /usr/tags
```

For more information, read

```
:help option-backslash
```

19.5. Can I add (embed) Vim option settings to the contents of a file?

You can use modelines to add Vim option settings to the contents of a file. For example, in a C file, you can add the following line to the top or the bottom of the file:

```
/* vim:sw=4: */
```

This will set the 'shiftwidth' option to 4, when editing that C file. For this to work, the 'modeline' option should be set. By default, the 'modeline' option is set. The 'modelines' settings specifies the number of lines that will be checked for the Vim set commands.

For more information, read

```
:help 21.6
:help modeline
:help auto-setting
:help 'modeline'
:help 'modelines'
```

19.6. How do I display the line numbers of all the lines in a file?

You can set the 'number' option to display the line numbers for all the lines.

```
:set number
```

For more information, read

```
:help 'number'
```

19.7. How do I change the width of the line numbers displayed using the "number" option?

The width used for displaying the line numbers for the 'number' option is hard-coded in Vim. It is not possible to change this width by setting some

option.

The request and the patch to add an option to change the number of columns used for the 'number' option is in the Vim todo list:

    "Add an option to set the width of the 'number' column. Eight
    positions is often more than needed. Or adjust the width to the length
    of the file?
    Add patch that adds 'numberlen' option. (James Harvey)
    Other patch with min and max from Emmanuel Renieris (2002 Jul 24)
    Other patch without an option by Gilles Roy (2002 Jul 25)"

19.8. How do I display (view) all the invisible characters like space, tabs and newlines in a file?

You can set the 'list' option to see all the invisible characters in your file.

```
:set list
```

With this option set, you can view space characters, tabs, newlines, trailing space characters and wrapped lines.

To not display the invisible characters (which is the default), you have to reset the 'list' option:

```
:set nolist
(or)
:set list!
```

The ":set list!" command will toggle the current setting of the boolean 'list' option.

You can modify the 'listchars' option to configure how and which invisible characters are displayed. For example, with the following command all the trailing space characters will be displayed with a '.' character.

```
:set listchars=trail:.
```

For more information, read

```
:help 'listchars'
:help 'list'
```

19.9. How do I configure Vim to always display the current line and column number?

You can set the 'ruler' option to display current column and line number in the status line:

```
:set ruler
```

For more information, read

```
:help 'ruler'
```

19.10. How do I display the current Vim mode?

You can set the 'showmode' option to display the current Vim mode. In Insert, Replace and Visual modes, Vim will display the current mode on the last line.

```
:set showmode
```

For more information, read

```
:help 'showmode'
```

19.11. How do I configure Vim to show pending/partial commands on the status line?

You can set the 'showcmd' option to display pending/partial commands in the status line:

```
:set showcmd
```

For more information, read

```
:help 'showcmd'
```

19.12. How do I configure the Vim status line to display different settings/values?

You can set the 'statusline' option to display different values/settings in the Vim status line.

For more information, read

```
:help 'statusline'
:help 'laststatus'
:help 'rulerformat'
:help 'ruler'
```

19.13. How do I configure Vim to display status line always?

You can set the 'laststatus' option to 2 to display the status line always.

```
:set laststatus=2
```

For more information, read

```
:help 'laststatus'
```

19.14. How do I make a Vim setting persistent across different Vim invocations/instances/sessions?

To make a Vim option setting persistent across different Vim instances, add your setting to the .vimrc or .gvimrc file. You can also use the ":mkvimrc" command to generate a vimrc file for the current settings.

For more information, read

```
:help save-settings
:help vimrc
:help gvimrc
:help vimrc-intro
:help :mkvimrc
:help initialization
```

19.15. Why do I hear a beep (why does my window flash) about 1 second after I hit the Escape key?

This is normal behavior. If your window flashes, then you've got the visual bell on. Otherwise, you should hear a beep.

Vim needs a timeout to tell the difference between a simple escape and, say, a cursor key sequence. When you press a key in normal mode (and even in insert mode) and that key is the beginning of a mapping, Vim waits a certain amount of time to see if the rest of the mapping sequence follows. If the mapping sequence is completed before a given timeout period, the mapping for that sequence of keys is applied. If you interrupt the mapping, the normal actions associated with the keys are executed.

For example, if you have a mapping defined as ":imap vvv Vim is great!!" and you type "vvv" quickly, the "Vim is great!!" will be inserted into your text. But if you type "vv v" then that is what will put into your text. This is also true if you type "vvv" too slowly where "too slowly" is longer than the value for the timeout option. Setting the timeout option to a larger value can help alleviate problems that appear when using function keys over a slow line.

For more information, read

```
:help ttimeout
```

19.16. How do I make the 'c' and 's' commands display a '$' instead of deleting the characters I'm changing?

To make the 'c' and 's' commands display a '$' instead of deleting the characters, add the $ flag to the 'cpoptions' option:

```
:set cpoptions+=$
```

For more information, read

```
:help 'cpoptions'
```

19.17. How do I remove more than one flag using a single ":set" command from a Vim option?

You can remove more than one flag from a Vim option using a single ":set" command, by specifying the flags in exactly the same order as they appear in the option. For example, if you use the following command to remove the 't' and 'n' flags from the 'formatoptions' option:

```
:set formatoptions-=tn
```

The 't' and 'n' flags will be removed from the 'formatoptions' option, only if the 'formatoptions' option contains these flags in this order: 'tn'. Otherwise, it will not remove the flags. To avoid this problem, you can remove the flags one by one:

```
:set formatoptions-=t formatoptions-=n
```

For more information, read

```
:help :set-=
```

===

SECTION 20 - MAPPING KEYS

20.1. How do I know what a key is mapped to?

To see what a key is mapped to, use the following commands:

```
:map <key>
:map! <key>
```

You can also check the mappings in a particular mode using one of the ":cmap", ":nmap", ":vmap", ":imap", ":omap", etc commands.

For more information, read

```
:help map-listing
:help map-overview
```

20.2. How do list all the user-defined key mappings?

You can list all the user-defined key mappings using:

```
:map
```

For more information, read

```
:help map-listing
```

20.3. How do I unmap a previously mapped key?

You can unmap a previously mapped key using the ":unmap" command:

```
:unmap <key>
:unmap! <key>
```

For mode specific mappings, you can use one of the ":nunmap/:vunmap/:ounmap/:iunmap/:lunmap/:cunmap" commands.

The following command will fail to unmap a buffer-local mapped key:

```
    :unmap <key>
```

To unmap a buffer-local mapped key, you have to use the <buffer> keyword in the unmap command:

```
    :unmap <buffer> <key>
    :unmap! <buffer> <key>
```

For more information, read

```
    :help :unmap
    :help map-modes
    :help map-local
    :help 'mapleader'
```

20.4. I am not able to create a mapping for the <xxx> key. What is wrong?

First make sure that the key is passed to Vim. In insert mode, press CTRL-V followed by the desired key. You should see the keycode corresponding to the key . If you do see the keycode, then you can create a mapping for the key using the following command:

```
    :map <C-V><xxx>  <your_command_to_be_mapped>
```

For more information, read

```
    :help map-keys-fails
    :help :map-special-keys
    :help key-codes
```

20.5. How do I map the numeric keypad keys?

First make sure that the numeric keypad keys are passed to Vim. Next, you can use the following command to map the numeric keypad keys:

```
    :map <kSomething>  <your_command>
```

where, <kSomething> can be kHome, kEnd, kPageUp, kPageDown, kPlus, kMinus, kDivide, kMultiply, kEnter, etc.

For more information, read

```
    :help key-codes
    :help terminal-options
```

20.6. How do I create a mapping that works only in visual mode?

You can create mappings that work only in specific mode (normal, command, insert, visual, etc). To create a mapping that works only in the visual mode, use the ":vmap" command:

```
    :vmap <F3> <your mapping here>
```

For more information, read

```
:help :vmap
:help map-modes
:help 40.1
```

20.7. In a Vim script, how do I know which keys to use for my mappings, so that the mapped key will not collide with an already used key?

Vim uses most of the keys in the keyboard. You can use the <leader> prefix in maps to define keys which will not overlap with Vim keys. For example:

```
:map <leader>S <C-W>s
:map <leader>j <C-W>j
:map <leader>k <C-W>k
```

where by default <leader> gets substituted with a backslash (\), so the user would enter

```
    \s
    \j
    \k
```

to invoke the above map commands. The user can change the mapleader variable to be whatever they wanted:

```
:let mapleader = ","
```

When writing a plugin or other script, more often than not, it is advisable to use :noremap instead of :map to avoid side effects from user defined mappings.

For more information, read

```
:help <Leader>
:help <LocalLeader>
:help write-plugin
```

20.8. How do I map the escape key?

You can map the Escape key to some other key using the ":map" command. For example, the following command maps the escape key to CTRL-O.

```
:map <C-O> <Esc>
```

20.9. How do I map a key to perform nothing?

You can map a key to <Nop> to perform nothing when the key is pressed. For example, with the following mappings, the <F7> key will do nothing when pressed.

```
:map <F7> <Nop>
:map! <F7> <Nop>
```

For more information, read

```
:help <Nop>
```

```
:help :map
:help :map!
:help map-modes
```

20.10. I want to use the Tab key to indent a block of text and Shift-Tab key to unindent a block of text. How do I map the keys to do this? This behavior is similar to textpad, visual studio, etc.

Use the following mapping:

```
:inoremap <S-Tab> <C-O><LT><LT>
:nnoremap <Tab> >>
:nnoremap <S-Tab> <LT><LT>
:vnoremap <Tab> >
:vnoremap <S-Tab> <LT>
```

Note that, the <S-Tab> mapping will work only if Vim receives the correct key sequence. This is mostly the case with GUI Vim.

For more information, read

```
:help :inoremap
:help :nnoremap
:help :vnoremap
:help <S-Tab>
:help i_CTRL-O
:help >>
:help <<
:help <LT>
```

20.11. In my mappings the special characters like <CR> are not recognized. How can I configure Vim to recognize special characters?

Check the value of the 'cpoptions' option:

```
:set cpoptions?
```

If this option contains the '<' flag, then special characters will not be recognized in mappings. Remove the '<' flag from 'cpoptions' option:

```
:set cpo-=<
```

Also, check the value of the 'compatible' option:

```
:se compatible?
```

The 'compatible' option must be reset:

```
:se nocompatible
```

For more information, read

```
:help 'cpoptions'
:help 'compatible'
```

20.12. How do I use the '|' to separate multiple commands in a map?

You can escape the '|' character using backslash (\) to use '|' in a map.

```
:map _l :!ls \| more<CR>
```

You can also try the following command:

```
:map _l :!ls <bar> more<CR>
```

There are also other ways to do this.

For more information, read

```
:help map_bar
```

20.13. If I have a mapping/abbreviation whose ending is the beginning of another mapping/abbreviation, how do I keep the first from expanding into the second one?

Instead of using the ":map lhs rhs" command, use the ":noremap lhs rhs" command. For abbreviations, use "noreabbrev lhs rhs". The "nore" prefix prevents the mapping or abbreviation from being expanded again.

For more information, read

```
:help :noremap
:help :noreabbrev
```

20.14. Why does it take a second or more for Vim to process a key, sometimes when I press a key?

Make sure you have not defined a mapping for this key using the following command:

```
:map <key>
```

If a mapping is defined for this key and the mapped key contains more than one character, then Vim will wait for the next character to be pressed to determine whether it is the mapped key or not. For example, if you have mapped "ab", then if you press "a", Vim will wait for the next key to be pressed. If the next key is "b", Vim will execute the mapped sequence. Otherwise, Vim will proceed with the normal processing of "a" followed by the next key. If the 'timeout' option is set (which is the default), then Vim will timeout after waiting for the period specified with the 'timeoutlen' option (default is 1 second).

For more information, read

```
:help map-typing
:help 'timeoutlen'
:help 'ttimeoutlen'
:help 'timeout'
:help 'ttimeout'
:help vt100-cursor-keys
:help slow-fast-terminal
```

20.15. How do I map a key to run an external command using a visually selected text?

You can the ":vmap" command to map a key in the visual mode. In the mapped command sequence, you have to first yank the text. The yanked text is available in the '"' register. Now, you can use the contents of this register to run the external command. For example, to run the external command "perldoc" on a visually selected text, you can use the following mapping:

```
:vmap <F7> y:!exec "!perldoc '" . @" . "'"<CR>
```

If you want the mapping to work in the visual mode, but not with the highlighted text, you can use the following command:

```
:vmap <F7> :<C-U>!perldoc <cword><CR>
```

The above mapping will use the word under the cursor instead of the highlighted text. Note the use of the <C-U> before invoking the "perldoc" external command. The <C-U> is used to erase the range of text selected in the visual mode and displayed on the command line. If the visual range is not removed using <C-U>, then the output from the external command will replace the visually selected text.

For more information, read

```
:help :vmap
:help quote_quote
:help let-register
:help c_CTRL-U
:help :!cmd
```

20.16. How do I map the Ctrl-I key while still retaining the functionality of the <Tab> key?

The Ctrl-I key and the <Tab> key produce the same keycode, so Vim cannot distinguish between the Ctrl-I and the <Tab> key. When you map the Ctrl-I key, the <Tab> key is also mapped (and vice versa). The same restriction applies for the Ctrl-[key and the <Esc> key.

For more information, read

```
:help keycodes
```

===

SECTION 21 - ABBREVIATIONS

21.1. How do I auto correct misspelled words?

You can auto correct misspelled words using abbreviations. For example, the following abbreviation can be used to correct "teh" with "the":

```
    :abbreviate teh the
```

Vim supports abbreviations in insert mode, replace mode and command-line mode.

For more information, read

```
    :help 24.7
    :help abbreviations
    :help Q_ab
```

21.2. How do I create multi-line abbreviations?

You can create multi-line abbreviations by embedding the "<CR>" key code in the text:

```
    iabbrev #c --------------<CR>-- Date:<CR>--<CR>---------
```

With the above abbreviation, when you type #c, it will be expanded to the following text:

```
--------------
-- Date:
--
---------
```

For more information, read

```
    :help abbreviations
```

21.3. When my abbreviations are expanded, an additional space character is added at the end of the expanded text. How do I avoid this character?

To avoid an additional space character at the end of the expanded text, you can expand the abbreviation by pressing the CTRL-] key. The abbreviation will be expanded without adding a space character at the end.

Another alternative is to use the following function and command:

```
function! Eatchar(pat)
    let c = nr2char(getchar())
    return (c =~ a:pat) ? '' : c
endfunction
command! -nargs=+ Iabbr execute "iabbr" <q-args> . "<C-R>=Eatchar('\\s')<CR>"
```

Now, define your abbreviations using the new "Iabbr" command instead of the builtin "iabbrev" command. With this command, after expanding the abbreviated text, the next typed space character will be discarded.

For more information, read

```
    :help abbreviations
```

21.4. How do I insert the current date/time stamp into the file?

You can use the strftime() function to insert the current data/time stamp in a file. For example, you can use the following abbreviation:

```
    iabbrev dts <C-R>=strftime("%y/%m/%d %H:%M")<CR>
```

With this abbreviation, when you type dts in insert mode, it will be expanded to the date/time stamp.

Some other forms of the above abbreviation are listed below:

```
    iabbrev mdyl <C-R>=strftime("%a %d %b %Y")<CR>
    iabbrev mdys <C-R>=strftime("%y%m%d")<CR>
    iabbrev mdyc <C-R>=strftime("%c")<CR>
    iabbrev hml  <C-R>=strftime("%d/%m/%y %H:%M:%S")<CR>
    iabbrev hms  <C-R>=strftime("%H:%M:%S")<CR>
```

For more information, read

```
    :help strftime()
    :help i_CTRL-R
```

21.5. How do I prevent an abbreviation from expanding in insert mode?

You can prevent an abbreviation from expanding in insert mode by typing CTRL-V before the character after the abrreviated word.

For more information, read

```
    :help abbreviations
```

===

SECTION 22 - RECORD AND PLAYBACK

22.1. How do I repeat an editing operation (insertion, deletion, paste, etc)?

You can repeat the last editing operation using the '.' command. This will repeat the last simple change like a insert, delete, change, paste, etc.

For more information, read

```
    :help 04.3
    :help single-repeat
    :help Q_re
```

22.2. How I record and repeat a set of key sequences?

You can use the 'q' command in normal mode to record a set of key sequences and store it in a register. For example, in the normal mode you can press q followed by a register name {0-9a-bA-Z"} to start the recording. To end/stop the recording press q again. You can playback/repeat the recorded key sequences by pressing @ followed by the register name. e.g. @a.

Another approach is to start Vim with the "-w" command-line argument.

```
$ vim -w <file_name>
```

Vim will record all the characters typed in the session in the specified file "file_name". You can use the recorded file with the "-s" command line argument to play it back:

```
$ vim -s <file_name>
```

For more information, read

```
:help 10.1
:help recording
:help -w
:help -s
```

22.3. How do I edit/modify a recorded set of key sequences?

The recorded key sequences are stored in a register. You can paste the contents of the register into a Vim buffer, edit the pasted text and again yank the text into the register. You can also use the ":let" command to modify the register. For example:

```
:let @a = "iHello World\<Esc>"
```

For more information, read

```
:help recording
:help 10.1
:help let-register
:help <>
:help 'cpoptions'
```

22.4. How do I write recorded key sequences to a file?

The recorded key sequences are stored in a register. You can paste the contents of the register into a Vim buffer. Now you can save the buffer into a file. You can also modify the pasted text and again yank into the register to modify the recorded key sequence. For example, if you record a set of key sequences using qa ..... q. The recorded key sequences are stored in the register 'a'. You can paste the contents of register 'a' using "ap.

For more information, read

```
:help recording
:help 10.1
```

22.5. I am using register 0 to record my key sequences (i.e. q0 .... q). In the recorded key sequences, I am yanking some text. After the first replay of the recorded key sequence, I am no longer able to play it back.

Register 0 contains the text from the last yank operation. In your recorded

key sequence, when the yank is performed, register 0 is overwritten with the yanked text. So your recording stored in register 0 is lost. You have to use some other register.

For more information, read

```
:help registers
```

===

SECTION 23 - AUTOCOMMANDS

23.1. How do I execute a command when I try to modify a read-only file?

You can use the FileChangedRO autocommand event to execute a command when a read-only file modified. For example, you can use this event to checkout a read-only file:

```
:autocmd FileChangedRO * call MyCheckoutFunction()
```

For more information, read

```
:help FileChangedRO
```

23.2. How do I execute a command every time when entering a buffer?

You can use the BufEnter autocommand event to execute a command every time when entering a buffer. For example:

```
:autocmd BufEnter *.c set formatoptions=croqt
```

For more information, read

```
:help BufEnter
```

23.3. How do I execute a command every time when entering a window?

You can use the WinEnter autocommand event to execute a command every time when entering a window. For example:

```
:autocmd WinEnter *.c call MyFunction()
```

For more information, read

```
:help WinEnter
```

23.4. From an autocmd, how can I determine the name of the file or the buffer number for which the autocommand is executed?

You can use the special words <afile> or <abuf> in an autocmd to get the name of the file or the buffer number for which the autocommand is executed.

For more information, read

```
:help :<afile>
:help :<abuf>
:help :<amatch>
```

23.5. How do I automatically save all the changed buffers whenever Vim loses focus?

You can define an autocommand for the FocusLost event which will save all the modified buffers whenever Vim loses focus:

```
:autocmd FocusLost * wall
```

For more information, read

```
:help FocusLost
:help :wall
```

23.6. How do I execute/run a function when Vim exits to do some cleanup?

You can use VimLeave autocmd event to execute a function just before Vim exists. For example,

```
:autocmd VimLeave * call MyCleanupFunction()
```

For more information, read

```
:help VimLeave
```

===

SECTION 24 - SYNTAX HIGHLIGHT

24.1. How do I turn off/on syntax highlighting?

By default, the Vim syntax highlighting is turned off. To enable the syntax highlighting, you can use one of the following commands:

```
:syntax enable

or

:syntax on
```

To disable the syntax highlighting, you can use the following command:

```
:syntax off
```

For more information, read

```
:help 06.1
:help 06.4
:help :syntax-enable
```

```
    :help :syntax-on
    :help :syn-clear
```

24.2. How do I change the background and foreground colors used by Vim?

Vim uses the "Normal" highlight group for the background and foreground colors. To change the foreground/background colors, you have to modify the "Normal" highlight group. For example, to set the background color to blue and foreground color to white, you can use

```
    :highlight Normal ctermbg=blue ctermfg=white guibg=blue guifg=white
```

If you are using the Motif or the Athena version of the GUI Vim, then you can modify the foreground and background resource names in the .Xdefaults files to change the colors:

```
    Vim.foreground:     Black
    Vim.backround:      Wheat
```

You can also use the "-foreground" and "-background" command-line arguments to specify the foreground and background colors. These arguments are supported only in the Motif or Athena versions:

```
    $ gvim -foreground Black -background Wheat
```

For more information, read

```
    :help :highlight
    :help .Xdefaults
    :help -gui
```

24.3. How do I change the highlight colors to suit a dark/light background?

You can set the 'background' option to either 'dark' or 'light' to change the highlight colors to suit a dark/light background:

```
    :set background=dark
```

For more information, read

```
    :help 'background'
    :help 6.2
```

24.4. How do I change the color of the line numbers displayed when the ":set number" command is used?

The line numbers displayed use the LineNr highlighting group. To display the current colors used, use

```
    :hi LineNr
```

To change the color modify the LineNr highlight group. For example:

```
    :hi linenr guifg=red guibg=black
```

This will give red numbers on a black background in GVIM.

For more information, read

```
:help :highlight
```

24.5. How do I change the background color used for a Visually selected block?

You can modify the 'Visual' highlight group to change the color used for a visually selected block:

```
:highlight Visual guibg=red
```

For more information, read

```
:help :highlight
:help hl-Visual
```

24.6. How do I highlight the special characters (tabs, trailing spaces, end of line, etc) displayed by the 'list' option?

You can modify the "NonText" and "SpecialKey" highlight groups to highlight the special characters displayed by the 'list' option:

```
:highlight NonText guibg=red
:highlight SpecialKey guibg=green
```

The "NonText" highlighting group is used for "eol", "extends" and "precedes" settings in the "listchars" option. The "SpecialKey" highlighting group is used for the "tab" and "trail" settings.

For more information, read

```
:help 'listchars'
:help hl-NonText
:help hl-SpecialKey
```

24.7. How do I specify a colorscheme in my .vimrc/.gvimrc file, so that Vim uses the specified colorscheme everytime?

You can specify the color scheme using the ":colorscheme" command in your .vimrc or .gvimrc file:

```
colorschme evening
```

For more information, read

```
:help :colorscheme
```

24.8. Vim syntax highlighting is broken. When I am editing a file, some parts of the file is not syntax highlighted or syntax highlighted incorrectly.

Vim doesn't read the whole file to parse the text for syntax highlighting. It starts parsing wherever you are viewing the file. That saves a lot of time, but sometimes the colors are wrong. A simple fix is refreshing the screen using the CTRL-L key. Or scroll back a bit and then forward again. You can also use the command:

```
:syntax sync fromstart
```

Note that this might considerably slow down the screen refreshing.

For more information, read

```
:help :syn-sync
:help :syn-sync-first
```

24.9. Is there a built-in function to syntax-highlight the corresponding matching bracket?

No. Vim doesn't support syntax-highlighting matching brackets. You can try using the plugin developed by Charles Campbell:

```
http://vim.sourceforge.net/tips/tip.php?tip_id=177
```

You can jump to a matching bracket using the '%' key. You can set the 'showmatch' option to temporarily jump to a matching bracket when in insert mode.

For more information, read

```
:help %
:help 'showmatch'
:help 'matchtime'
:help 'matchpairs'
```

24.10. How do I turn off the C comment syntax highlighting?

You can use the following command to turn off C comment syntax highlighting:

```
:highlight clear comment
```

For more information, read

```
:help c-syntax
```

24.11. How do I add my own syntax extensions to the standard syntax files supplied with Vim?

You should not modify the syntax files supplied with Vim to add your extensions. When you install the next version of Vim, you will lose your changes. Instead you should create a file under the ~/.vim/after/syntax directory with the same name as the original syntax file and add your additions to this file.

For more information, read

```
:help mysyntaxfile-add
:help 'runtimepath'
```

24.12. How do I replace a standard syntax file that comes with the Vim distribution with my own syntax file?

You can replace a standary syntax file that comes with the Vim distribution by creating a file with the same name as the original syntax file and placing it in the vim runtime syntax (~/.vim/syntax) directory. For example, to replace the c.vim syntax file in a Unix system, place the new c.vim in the ~/.vim/syntax directory. In a MS-Windows system, place the new syntax file in the $HOME/vimfiles/syntax or $VIM/vimfiles/syntax directory.

For more information, read

```
:help mysyntaxfile-replace
:help 44.11
:help mysyntaxfile
```

24.13. How do I highlight all the characters after a particular column?

You can use the ":match" command to highlight all the characters after a particular column:

```
:match Todo '\%>75v.\+'
```

This will highlight all the characters after the 75th column.

For more information, read

```
:help :match
:help /\%v
:help /\+
:help /.
```

24.14. How do I convert a source file (.c, .h, etc) with the Vim syntax highlighting into a HTML file?

You can use the 2html.vim script to convert a source file into a HTML file with the Vim syntax highlighting. Use the following command:

```
:runtime! syntax/2html.vim
```

For more information, read

```
:help convert-to-HTML
```

24.15. How do I list the definition of all the current highlight groups?

You can list the definition of all the current highlight groups using the ":highlight" (without any arguments) ex command.

For more information, read

```
:help :highlight
```

===

SECTION 25 - VIM SCRIPT WRITING

25.1. How do I list the names of all the scripts sourced by Vim?

You can use the ":scriptnames" command to list the names of all the scripts sourced by Vim:

```
:scriptnames
```

For more information, read

```
:help :scriptnames
```

25.2. How do I debug Vim scripts?

Vim has built-in support for a primitive debugger to debug Vim plugins and scripts. Using this debugger you can set breakpoints and step through the plugin functions.

For more information, read

```
:help debug-scripts
:help -D
```

25.3. How do I locate the script/plugin which sets a Vim option?

You can use the ":verbose" command to locate the plugin/script which last modified a Vim option. For example:

```
:verbose set textwidth?
```

For more information, read

```
:help :set-verbose
:help :verbose
```

25.4. I am getting some error/informational messages from Vim (possibly when running a script), the messages are cleared immediately. How do I display the messages again?

You can use the ":messages" command to display the previous messages.

```
:messages
```

For more information, read

```
:help :messages
:help :echoerr
```

```
:help :echomsg
:help message-history
```

25.5. How do I save and restore a plugin specific information across Vim invocations?

Vim will save and restore global variables that start with an uppercase letter and don't contain a lower case letter. For this to work, the 'viminfo' option must contain the '!' flag. Vim will store the variables in the viminfo file.

For more information, read

```
:help 'viminfo'
:help viminfo-file
:help variables
```

25.6. How do I start insert mode from a Vim function?

You can use the ":startinsert" command to start the insert mode from inside a Vim function.

For more information, read

```
:help :startinsert
```

25.7. How do I change the cursor position from within a Vim function?

You can use the cursor() function to position the cursor.

```
call cursor(lnum, col)
```

You can also use the following command to change the cursor position:

```
exe "normal! " . lnum . "G" . col . "|"
```

For more information, read

```
:help cursor()
:help bar
```

25.8. How do I check the value of an environment variable in the .vimrc file?

You can use prefix the environment variable name with the '$' character to use it from a Vim script/function. You can refer to the value of an environment variable using the $env_var syntax:

```
if $EDITOR == 'vi'
endif
```

For more information, read

```
:help expr-env
```

25.9. How do I check whether an environment variable is set or not from a Vim function?

You can use the exists() function to check for the existence of a environment variable.

```
if exists("$MY_ENV_VAR")
endif
```

For more information, read

```
:help exists()
:help expr-env
```

25.10. How do I call/use the Vim built-in functions?

You can use the ":call" command to invoke a Vim built-in function:

```
:call cursor(10,20)
```

You can use the ":echo" command to echo the value returned by a function:

```
:echo char2nr('a')
```

You can use the ":let" command to assign the value returned by a function to a variable:

```
:let a = getline('.')
```

To store the return value from a function into a Vim register, you can use the following command:

```
:let @a = system('ls')
```

The above command will store the return value from the 'ls' command into the register 'a'.

For more information, read

```
:help :call
:help :echo
:help :let
:help :let-register
:help user-functions
:help usr_41.txt
```

25.11. I am using some normal mode commands in my Vim script. How do I avoid using the user-defined mappings for these normal mode commands and use the standard Vim functionality for these normal mode commands?

You can use the "normal!" command in your script to invoke a normal-mode command. This will use the standard functionality of the normal mode command and will not use the user-defined mapping.

For more information, read

```
:help :normal
```

25.12. How do I get the current visually selected text into a Vim variable or register?

You can get the current visually selected text into a Vim variable by yanking the text into Vim register and then assigning the contents of the register into the variable:

```
:normal! gvy
:let myvar = @"
```

The above command copies the visually selected text into the variable "myvar".

You can also use the command:

```
:normal! gv"*y
```

In the above command, gv reselects the last visually selected text and the rest of the command copies the selected text into the * (clipboard) register. Alternatively, you can set the 'a' flag in the 'guioptions' option to automatically copy a visually selected text into the * register. To do this as part of a visual map, you can use a command similar to the one shown below:

```
:vmap <F3> "*y:call ...
```

For more information, read

```
:help gv
:help :normal
:help let-@
:help quotestar
:help clipboard
:help registers
```

25.13. I have some text in a Vim variable 'myvar'. I would like to use this variable in a ":s" substitute command to replace a text 'mytext'. How do I do this?

You can use the 'execute' command to evaluate the variable:

```
:execute '%s/mytext/' . myvar . '/'
```

For more information, read

```
:help :execute
```

You can also use "\=" in the substitute command to evaluate the variable:

```
:%s/mytext/\=myvar/
```

For more information, read

```
:help sub-replace-special
```

25.14. A Vim variable (bno) contains a buffer number. How do I use this variable to open the corresponding buffer?

The :buffer command will not accept a variable name. It accepts only a buffer number or buffer name. You have to use the ":execute" command to evaluate the variable into the corresponding value. For example:

```
:execute "buffer " . bno
```

For more information, read

```
:help :execute
```

25.15. How do I store the value of a Vim option into a Vim variable?

You can prefix the option name with the '&' character and assign the option value to a Vim variable using the "let" command. For example, to store the value of the 'textwidth' option into the Vim variable "old_tw", you can use the following command:

```
:let old_tw = &tw
```

To do the opposite, to set the 'textwidth' option with the value stored in the 'old_tw' variable, you can use the following command:

```
:let &tw = old_tw
```

For more information, read

```
:help expr-option
:help let-option
```

25.16. I have copied and inserted some text into a buffer from a Vim function. How do I indent the inserted text from the Vim function?

You can use the following command to format the just inserted text:

```
:normal '[=']
```

For more information, read

```
:help '[
:help ']
:help =
:help :normal
```

25.17. How do I get the character under the cursor from a Vim script?

You can use the getline() function and use string index [] to get the character:

```
:echo getline(".")[col(".") - 1]
```

In the above command, getline(".") returns the text in the current line. The indexing of the string starts at zero, and you can get a single character in a string by its index with the "string[index]" notation. The col(".") returns the column of the cursor position; the adjustment is to get the right character of the string.

Alternatively, you can use the following sequence of commands to get the character under the cursor:

```
normal! vy
let ch=@"
```

Note that the above commands will change the '< and '> marks.

For more information, read

```
:help getline()
:help col()
:help expr-[]
```

25.18. How do I get the name of the current file without the extension?

You can get the name of the current file without the extension using:

```
:echo expand("%:r")
```

With some commands, you can use the file name modifiers directly:

```
:cd %:p:h
:!gcc -o %:r.o %
```

For more information, read

```
:help filename-modifiers
:help expand()
:help cmdline-special
:help fnamemodify()
```

25.19. How do I get the basename of the current file?

You can use the :t filename modifier to get the basename of the current file:

```
:echo expand("%:t")
```

For more information, read

```
:help filename-modifiers
```

25.20. How do I get the output from a Vim function into the current buffer?

You can insert the return value from a function using the following command

in insert mode:

```
<C-R>=MyFunc()
```

Note that this will only insert the return value of the function.

For more information, read

```
:help i_CTRL-R
:help i_CTRL-R_CTRL-R
:help i_CTRL-R_CTRL-O
:help expression
```

25.21. How do I call external programs from a Vim function?

There are several ways to call external programs from a Vim function. You can use the builtin system() function to invoke external programs and get the result:

```
:let output = system("ls")
```

You can also use "!" ex-command to run an external command.

For more information, read

```
:help system()
:help :!
:help 10.9
```

25.22. How do I get the return status of a program executed using the ":!" command?

You can use the predefined Vim v:shell_error variable to get the return status of the last run shell command.

For more information, read

```
:help v:shell_error
```

25.23. How do I determine whether the current buffer is modified or not?

You can check the value of the 'modified' option to determine whether the current buffer is modified:

```
:set modified?
```

From a Vim script, you can check the value of the 'modified' option:

```
if &modified
    echo "File is modified"
endif
```

For more information, read

```
:help 'modified'
```

25.24. I would like to use the carriage return character in a normal command from a Vim script. How do I specify the carriage return character?

You can use the ":execute" command to specify the special (control) character in a normal mode command:

```
:execute "normal \<CR>"
:execute "normal ixxx\<Esc>"
```

For more information, read

```
:help :execute
:help expr-quote
```

25.25. How do I split long lines in a Vim script?

You can split long lines in a Vim script by inserting the backslash character ("\") at the start of the next line. For example,

For more information, read

```
:help line-continuation
```

25.26. When I try to "execute" my function using the "execute 'echo Myfunc()'" command, the cursor is moved to the top of the current buffer. Why?

The ":execute" command runs the normal mode command specified by the argument. In the case of the following command:

```
:execute "echo Myfunc()"
```

The call to "echo Myfunc()" will return 0. The ":execute" command will run the normal mode command "0", which moves the cursor to the top of the file. To call a Vim function, you should use the ":call" command instead of the ":execute" command:

```
:call Myfunc()
```

For more information, read

```
:help :call
:help :execute
:help :echo
:help user-functions
:help 41.5
:help 41.6
```

25.27. How do I source/execute the contents of a register?

If you have yanked a set of Vim commands into a Vim register (for example register 'a'), then you can source the contents of the register using one

of the following commands:

```
    :@a
or
    :exe @a
```

For more information, read

```
    :help :@
```

25.28. After calling a Vim function or a mapping, when I press the 'u' key to undo the last change, Vim undoes all the changes made by the mapping/function. Why?

When you call a function or a mapping, all the operations performed by the function/mapping are treated as one single operation. When you undo the last operation by pressing 'u', all the changes made by the function/mapping are reversed.

For more information, read

```
    :help undo-redo
    :help map-undo
```

25.29. How can I call a function defined with s: (script local function) from another script/plugin?

The s: prefix for a Vim function name is used to create a script local function. A script local function can be called only from within that script and cannot be called from other scripts. To define a function in a script/plugin, so that it can be called from other plugins/scripts, define the function without the s: prefix.

For more information, read

```
    :help script-variable
    :help script-local
    :help :scriptnames
```

25.30. Is it possible to un-source a sourced script? In otherwords, reverse all the commands executed by sourcing a script.

No. It is not possible to reverse or undo all the commands executed by sourcing a script.

For more information, read

```
    :help :source
```

===

SECTION 26 - PLUGINS

26.1. How do I set different options for different types of files?

You can create filetype plugins to set different options for different types of files. You should first enable filetype plugins using the command:

```
    :filetype plugin on
```

A filetype plugin is a vim script that is loaded whenever Vim opens or creates a file of that type. For example, to ensure that the 'textwidth' option is set to 80 when editing a C program (filetype 'c'), create one of the following files:

```
        ~/.vim/ftplugin/c.vim (Unix)
        %HOME%\vimfiles\ftplugin\c.vim (Windows)
```

with the following text in it:

```
        setlocal textwidth=80
```

You can also use autocommands to set specific options when editing specific type of files. For example, to set the 'textwidth' option to 75 for only *.txt files, you can use the following autocmd:

```
    autocmd BufRead *.txt setlocal textwidth=80
```

For more information, read

```
    :help filetype-plugin
    :help add-filetype-plugin
    :help autocmd
    :help 40.3
```

26.2. I have downloaded a Vim plugin or a syntax file or a indent file, or a color scheme or a filetype plugin from the web. Where should I copy these files so that Vim will find them?

You can place the Vim runtime files (plugins, syntax files, indent files, color schemes, filetype plugins, etc) under one of the directories specified in the 'runtimepath' option. To determine the current value of the 'runtimepath' option, use the following command:

```
    :set runtimepath
```

For Unix systems, this is usally the "$HOME/.vim" directory. For MS-Windows systems, this is usually the $VIM\vimfiles or $HOME\vimfiles directory. Depending on the type of the runtime file, you have to place it under a specific directory under the above runtime directory. The names of the directories are listed below:

```
    colors/   - color scheme files
    compiler/ - compiler files
    doc/      - documentation
    ftplugin/ - filetype plugins
    indent/   - indent scripts
    keymap/   - key mapping files
    lang/     - menu translations
    plugin/   - plugin scripts
```

```
    syntax/   - syntax files
    tutor/    - files for vimtutor
```

For more information, read

```
    :help your-runtime-dir
    :help 'runtimepath'
    :help :runtime
```

26.3. How do I extend an existing filetype plugin?

You can extend an existing filetype plugin by creating a file under either the $VIMRTUNTIME/after/ftplugin or the $VIMRTUNTIME/ftplugin directory. The name of the file should be the same as the name of the existing filetype plugin file. You can place your additions to the new file.

If you placed the file in the after/ftplugin runtime directory, then Vim will first source the existing filetype plugin file and then will source the new file. If you placed the file in the $VIMRTUNTIME/ftplugin runtime directory, then Vim will first source the new file and then will source the existing filetype plugin file.

For more information, read

```
    :help ftplugin-overrule
    :help filetype-plugin
    :help add-filetype-plugin
    :help 'runtimepath'
```

26.4. How do I turn off loading the Vim plugins?

You can reset the 'loadplugins' option to turn off loading the plugins:

```
    :set noloadplugins
```

You can also specify the "--noplugin" command line argument to stop loading the plugins:

```
    $ vim --noplugin
```

For more information, read

```
    :help 'loadplugins'
    :help --noplugin
    :help load-plugins
```

26.5. How do I turn on/off loading the filetype plugins?

By default, Vim will not load the filetype plugins. You can configure Vim to load filetype plugins using the command:

```
    filetype plugin on
```

You can turn off loading the filetype plugins using:

```
    filetype plugin off
```

For more information, read

```
    :help filetype-plugin-on
    :help filetype-plugin-off
    :help :filetype
```

26.6. How do I override settings made in a file type plugin in the global ftplugin directory for all the file types?

You can use an autocommand triggered on the FileType event:

```
    au Filetype * set formatoptions=xyz
```

This should at least be after "filetype on" in your vimrc. Best is to put it in your "myfiletypefile" file, so that it's always last.

If you want to override a setting for a particular filetype, then create a file with the same name as the original filetype plugin in the ~/.vim/after/ftplugin directory For example, to override a setting in the c.vim filetype plugin, create a c.vim file in the ~/.vim/after/ftplugin directory and add your preferences in this file.

For more information, read

```
    :help ftplugin-overrule
    :help ftplugins
    :help myfiletypefile
```

26.7. How do I disable the Vim directory browser plugin?

To disable the directory browsing Vim plugin, add the following line to your .vimrc file:

```
    let loaded_explorer = 1
```

For more information, read

```
    :help file-explorer
```

26.8. How do I set the filetype option for files with names matching a particular pattern or depending on the file extension?

You can set the 'filetype' option for files with names matching a particular pattern using an autocmd. For example, to set the 'filetype' option to 'c' for all files with extension '.x', you can use the following autocmd:

```
    autocmd! BufRead,BufNewFile *.x     setfiletype c
```

A better alternative to the above approach is to create a filetype.vim file in the ~/.vim directory (or in one of the directories specified in the 'runtimepath' option) and add the following lines:

```
    " my filetype file
    if exists("did_load_filetypes")
        finish
    endif
    augroup filetypedetect
        au! BufRead,BufNewFile *.x       setfiletype c
    augroup END
```

For more information, read

```
    :help new-filetype
    :help 43.2
    :help :setfiletype
```

===

SECTION 27 - EDITING PROGRAM FILES

27.1. How do I enable automatic indentation for C/C++ files?

You can enable file-type based indentation using:

```
    :filetype indent on
```

If you want to only enable automatic C indentation, then use:

```
    :set cindent
```

For more information, read

```
    :help 'cindent'
    :help C-indenting
    :help filetype
```

27.2. How do I configure the indentation used for C/C++ files?

You can configure the Vim C indentation by modifying the value of the 'cinoptions', 'cinkeys' and 'cinwords' options.

For more information, read

```
    :help 'cindent'
    :help 'cinoptions'
    :help 'cinkeys'
    :help 'cinwords'
    :help C-indenting
    :help cinoptions-values
    :help 'smartindent'
```

27.3. How do I turn off the automatic indentation feature?

By default, the automatic indentation is not turned on. You must have configured Vim to do automatic indentation in either .vimrc or .gvimrc files. You can disable automatic indentation using either,

```
    :filetype indent off
```

or

```
    :set nocindent
```

Also, check the setting for the following options:

```
    :set autoindent?
    :set smartindent?
    :set indentexpr?
```

For more information, read

```
    :help 'cindent'
    :help filetype-indent-off
    :help 'autoindent'
    :help 'smartindent'
    :help 'indentexpr'
```

27.4. How do I change the number of space characters used for the automatic indentation?

You can modify the 'shiftwidth' option to change the number of space characters used for the automatic indentation:

```
    :set shiftwidth=4
```

For more information, read

```
    :help 'shiftwidth'
```

27.5. I am editing a C program using Vim. How do I display the definition of a macro or a variable?

You can use the [d command to display the definition of a macro and the [i command to display the definition of a variable.

For more information, read

```
    :help [d
    :help [i
    :help include-search
    :help 29.4
    :help 29.5
```

27.6. I am editing a C program using Vim. How do I jump to the beginning or end of a code block from within the block?

You can use '[{' command to jump to the beginning of the code block and ']} to jump to the end of the code block from inside the block.

For more information, read

```
:help [{
:help ]}
:help various-motions
```

27.7. Is there a way to turn off the "//" comment auto-insertion behavior for C++ files? If I'm sitting on a line beginning with "//", then I open a new line above or below it, Vim automatically inserts new "//" chars.

You can modify the value of the 'comments' option to stop Vim from inserting the C++ comment character ("//") automatically. For example:

```
:set comments=sr:/*,mb:*,el:*/
```

For more information, read

```
:help 'comments'
:help format-comments
```

27.8. How do I add the comment character '#' to a set of lines at the beginning of each line?

First, select the first character in all the lines using visual block mode (CTRL-V). Press 'I' to start inserting characters at the beginning of the line. Enter the comment character and then stop the insert mode by pressing <Esc>. Vim will automatically insert the entered characters at the beginning of all the selected lines.

For more information, read

```
:help visual-block
:help blockwise-operators
:help v_b_I
```

27.9. How do I edit a header file with the same name as the corresponding C source file?

You can use the following command to edit a header file with the same name as the corresponding C source file:

```
:e %:t:r.h
```

You can use the following command to edit the file in a new split window:

```
:sp %:t:r.h
```

In the above commands, the percent sign expands to the name of the current file. The ":t" modifier extracts the tail (last component) of the filename. The ":r" modifier extracts the root of the filename. The .h is appended to the resulting name to get the header filename.

Another approach is to use the following command:

```
:sfind %:t:r.h
```

This command will search for the header file in the directories specified
in the 'path' option.

For more information, read

```
:help cmdline-special
:help filename-modifiers
:help :sfind
:help 'path'
```

27.10. How do I automatically insert comment leaders while typing comments?

To automatically insert comment leaders while typing comments, add the 'r'
and 'o' flags to the 'formatoptions' option.

```
:set formatoptions+=ro
```

You may also want to add the 'c' flag to auto-wrap comments using the
'textwidth' option setting and the 'q' flag to format comments with the
"gq" command:

```
:set formatoptions=croq
```

For more information, read

```
:help 30.6
:help format-comments
:help 'comments'
:help fo-table
```

===

SECTION 28 - QUICKFIX

28.1. How do I build programs from Vim?

You can use the ":make" command to build programs from Vim. The ":make"
command runs the program specified by the 'makeprg' option.

For more information, read

```
:help 30.1
:help make_makeprg
:help 'makeprg'
:help 'makeef'
:help :make
:help quickfix
```

28.2. When I run the make command in Vim I get the errors listed as the
compiler compiles the program. When it finishes this list disappears
and I have to use the :clist command to see the error message again.
Is there any other way to see these error messages?

You can use the ":copen" or ":cwindow" command to open the quickfix window

that contains the compiler output. You can select different error lines from this window and jump to the corresponding line in the source code.

For more information, read

```
:help :copen
:help :cwindow
:help quickfix
```

===

SECTION 29 - FOLDING

29.1. How do I extend the Vim folding support?

You can use the 'foldexpr' option to fold using an user specified function. For example, to fold subroutines of the following form into a single line:

```
sub foo {
  my $barf;
  $barf = 3;
  return $barf;
}
```

You can use the following commands:

```
set foldmethod=expr
set foldexpr=MyFoldExpr(v:lnum)
fun! MyFoldExpr(line)
    let str = getline(a:line)
    if str =~ '^sub\>'
        return '1'
    elseif str =~ '^}'
        return '<1'
    else
        return foldlevel(a:line - 1)
    endif
endfun
```

For more information, read

```
:help 'foldexpr'
:help fold-expr
```

29.2. When I enable folding by setting the 'foldmethod' option, all the
      folds are closed. How do I prevent this?

You can set the 'foldlevelstart' option to a particular value to close only folds above the specified value.

```
:set foldlevelstart=99
```

For more information, read

```
:help 'foldlevelstart'
```

```
    :help 'foldlevel'
    :help fold-foldlevel
```

29.3. How do I control how many folds will be opened when I start editing a file?

You can modify the 'foldlevelstart' option to control the number of folds that will be opened when you start editing a file. To start editing with all the folds closed:

```
    :set foldlevelstart=0
```

To start editing with all the folds opened, you can use

```
    :set foldlevelstart=999
```

For more information, read

```
    :help 'foldlevelstart'
```

29.4. How do I open and close folds using the mouse?

You can click on the + and - characters displayed at the leftmost column to open and close fold. For this to work, you have to set the 'foldcolumn' to a value greater than zero:

```
    :set foldcolumn=2
```

For more information, read

```
    :help 'foldcolumn'
```

29.5. How do I change the text displayed for a closed fold?

You can use the 'foldtext' option to change the text displayed for a closed fold.

For more information, read

```
    :help 'foldtext'
    :help fold-foldtext
    :help 'fillchars'
```

29.6. How do I store and restore manually created folds across different Vim invocations?

You can use the ":mkview" command to store manually created folds. Later, you can use the ":loadview" command to restore the folds. For this to work, the 'viewoptions' must contain "folds".

For more information, read

```
    :help 28.4
    :help :mkview
```

```
    :help :loadview
    :help 'viewoptions'
    :help 'viewdir'
    :help :mksession
    :help 'sessionoptions'
```

===

SECTION 30 - VIM WITH EXTERNAL APPLICATIONS

30.1. Can I run a shell inside a Vim window?

Currently Vim doesn't have support for running shell and other external commands inside a Vim window.

For more information, read

```
    :help shell-window
```

Alternatively, you can try using the Unix "screen" utility or the 'splitvt' program.

You can also use the vimsh plugin by Brian Sturk to run a shell in a Vim window. To use this you need to have Vim built with python support. For more information visit the following URL:

```
    http://vim.sourceforge.net/scripts/script.php?script_id=165
```

30.2. How do I pass the word under the cursor to an external command?

You can use the special keyword <cword> to pass the word under the cursor to an external command. For example:

```
    :!dict <cword>
```

For more information, read

```
    :help <cword>
```

30.3. How do I get the output of a shell command into a Vim buffer?

You can use the ":r !" command to get the output of a shell command into a Vim buffer. For example, to insert the output of the "ls" shell command, you can use the following command:

```
    :r !ls
```

To insert the output of the shell command above the first line use the following command:

```
    :0r !ls
```

For more information, read

    :help :r!

30.4. How do I pipe the contents of the current buffer to an external command and replace the contents of the buffer with the output from the command?

You can use the :! command to pipe the contents of the current buffer to a external command and replace the contents of the buffer with the output from the command. For example, to sort the contents of the current buffer, using the Unix sort command, you can use the following command:

```
:%!sort
```

To sort only lines 10-20, you can use the following command

```
:10,20!sort
```

Also, if you want to pipe a buffer to an external command but not put the results back in the buffer, you can use

```
:w !sort
```

The above command will pipe the entire buffer to the sort command. Note that the space between the 'w' and the '!' is critical. To pipe only a range of lines, you can use

```
:10,20w !sort
```

The above command will pipe the lines 10-20 to the sort command.

For more information, read

```
:help :range!
:help 10.9
:help :w_c
```

30.5. How do I sort a section of my file?

You can pipe a section of the file to the Unix "sort" utility to sort the file. For example:

```
:5,100!sort
```

You can also use a visual block, and use the "!sort" command on the selected block.

To sort using visual blocks (sort based on a column or sort just the column itself), read the following tip from the Vim online web page:

http://vim.sourceforge.net/tips/tip.php?tip_id=588

30.6. Is there a step-by-step guide for using Vim with slrn?

Visit the following link to get information about using Vim with Slrn:

    http://thingy.apana.org.au/~fun/slrn/

30.7. How do I use Vim as a pager?

You can use Vim as a pager using the $VIMRUNTIME/macros/less.sh shell script, supplied as part of the standard Vim distribution. This shell script uses the $VIMRUNTIME/macros/less.vim Vim script to provide less like key bindings.

For more information, read

```
:help less
```

30.8. How do I view Unix man pages from inside Vim?

You can view Unix man pages, inside Vim, using the man.vim plugin supplied as part of the standard Vim distribution. To use this plugin, add the following line to your startup vimrc file:

```
runtime ftplugin/man.vim
```

You can also press the K key to run the program specified by the 'keywordprg' option with the keyword under the cursor. By default, 'keywordprg' is set to run man on the keyword under the cursor.

For more information, read

```
:help man-plugin
:help K
:help 'keywordprg'
```

30.9. How do I change the diff command used by the Vim diff support?

By default, the Vim diff support uses the 'diff' command. You can change this by changing the 'diffexpr' option.

For more information, read

```
:help diff-diffexpr
:help 'diffexpr'
```

30.10. How do I use the Vim diff mode without folding?

You can use the following command-line to start Vim with two filenames and use the diff mode without folding:

```
$ vim -o file1 file2 "+windo set diff scrollbind scrollopt+=hor nowrap"
```

If you like vertically split windows, then replace "-o" with "-O".

For more information, read

```
:help vimdiff
```

===

SECTION 31 - GUI VIM

31.1. How do I create buffer specific menus?

Adding support for buffer specific menus is in the Vim TODO list. In the mean time, you can try Michael Geddes's plugin, buffermenu.vim:

```
http://vim.sourceforge.net/scripts/script.php?script_id=246
```

31.2. How do I change the font used by GUI Vim?

You can change the 'guifont' option to change the font used by GUI Vim. To display the current value of this option, you can use

```
:set guifont?
```

You can add the displayed font name to the .vimrc file to use the font across Vim sessions. For example, add the following line to the .vimrc file to use Andale Mono font.

```
set guifont=Andale_Mono:h10:cANSI
```

For Win32, GTK and Photon version of Vim, you can use the following command to bringup a dialog which will help you in changing the guifont:

```
:set guifont=*
```

You can also use the -font Vim command line option to specify the font used for normal text.

For more information, read

```
:help 'guifont'
:help 'guifontset'
:help 'guifontwide'
:help font-sizes
:help -font
:help -boldfont
:help -italicfont
:help -menufont
:help -menufontset
```

31.3. When starting GUI Vim, how do I specify the location of the GVIM window?

You can use the "-geometry" command line argument to specify the location of the GUI Vim window. For example:

```
$ gvim -geometry 80x25+100+300
```

For more information, read

```
    :help 31.4
    :help -geom
```

31.4. How do I add a horizontal scrollbar in GVim?

You can enable the horizontal scrollbar by modifying the 'guioptions' option:

```
    :set guioptions+=b
```

For more information, read

```
    :help 'guioptions'
    :help gui-horiz-scroll
```

31.5. How do I make the scrollbar appear in the left side by default?

You can add the 'l' flag to the 'guioptions' option to make the scrollbar appear in the left side.

```
    :set guioptions+=l
    :set guioptions-=r
```

For more information, read

```
    :help 'guioptions'
    :help gui-scrollbars
```

31.6. How do I remove the Vim menubar?

You can remove the Vim menubar by removing the 'm' flag from the 'guioptions' option:

```
    :set guioptions-=m
```

For more information, read

```
    :help 'guioptions'
```

31.7. I am using GUI Vim. When I press the ALT key and a letter, the menu starting with that letter is selected. I don't want this behavior as I want to map the ALT-<key> combination. How do I do this?

You can use the 'winaltkeys' option to disable the use of the ALT key to select a menu item:

```
    :set winaltkeys=no
```

For more information, read

```
    :help 'winaltkeys'
    :help :simalt
```

31.8. Is it possible to scroll the text by dragging the scrollbar so that the cursor stays in the original location?

The way Vim is designed, the cursor position has to be in a visible spot in normal, visual, select and insert mode. This cannot be changed without modifying Vim. When the scrollbar is used, the cursor will be moved so that it is always visible. Another approach to solving this problem is to use the Vim marks. You can mark the current cursor position using ma. Then scroll to a different part of the text and jump back to the old position using `a. You can also try the following suggestion from the Vim Online website:

```
http://www.vim.org/tip_view.php?tip_id=320
```

For more information, read

```
:help mark-motions
```

31.9. How do I get gvim to start browsing files in a particular directory when using the ":browse" command?

You can set the 'browsedir' option to the default directory to use for the ":browse" command.

```
:set browsedir='<your_dir>'
```

For more information, read

```
:help 'browsedir'
```

31.10. For some questions, like when a file is changed outside of Vim, Vim displays a GUI dialog box. How do I replace this GUI dialog box with a console dialog box?

You can set the 'c' flag in the 'guioptions' option to configure Vim to use console dialogs instead of GUI dialogs:

```
:set guioptions+=c
```

For more information, read

```
:help 'guioptions'
```

31.11. I am trying to use GUI Vim as the editor for my xxx application. When the xxx application launches GUI Vim to edit a file, the control immediately returns to the xxx application. How do I start GUI Vim, so that the control returns to the xxx application only after I quit Vim?

You have to start GUI Vim with the '-f' (foreground) command line option:

```
$ gvim -f
```

By default, GUI Vim will disconnect from the program that started Vim. With the '-f' option, GUI Vim will not disconnect from the program that started

it.

For more information, read

```
:help gui-fork
:help -f
```

31.12. Why does the "Select Font" dialog doesn't show all the fonts
       installed in my system?

Vim supports only fixed width (mono-spaced) fonts. Proportional fonts are not supported. In the "Select Font" dialog, only fixed width fonts will be displayed.

For more information, read

```
:help font-sizes
:help 'guifont'
```

31.13. How do I use the mouse in Vim command-line mode?

You can set the 'c' flag in the 'mouse' option to use mouse in the Vim command-line mode:

```
:set mouse+=c
```

For more information, read

```
:help mouse-using
:help gui-mouse
:help 09.2
```

31.14. When I use the middle mouse button to scroll text, it pastes the
       last copied text. How do I disable this behavior?

You can map the middle mouse button to <Nop> to disable the middle mouse button:

```
:map  <MiddleMouse> <Nop>
:map! <MiddleMouse> <Nop>
```

For more information, read

```
:help gui-mouse-mapping
:help <Nop>
```

31.15. How do I change the location and size of a GUI Vim window?

You can use the "winpos" command to change the Vim window position. To change the size of the window, you can modify the "lines" and "columns" options.

For example, the following commands will position the GUI Vim window at the X,Y co-ordinates 50,50 and set the number of lines to 50 and the number of

columsn to 80.

```
:winpos 50 50
:set lines=50
:set columns=80
```

The arguments to the 'winpos' command specify the pixel co-ordinates of the Vim window. The 'lines' and 'columns' options specify the number of lines and characters to use for the height and the width of the window respectively.

For more information, read

```
:help 31.4
:help :winpos
:help 'lines'
:help 'columns'
:help GUIEnter
```

===

SECTION 32 - VIM ON UNIX

32.1. I am running Vim in a xterm. When I press the CTRL-S key, Vim freezes. What should I do now?

Many terminal emulators and real terminal drivers use the CTRL-S key to stop the data from arriving so that you can stop a fast scrolling display to look at it (also allowed older terminals to slow down the computer so that it did not get buffer overflows). You can start the output again by pressing the CTRL-Q key.

When you press the CTRL-S key, the terminal driver will stop sending the output data. As a result of this, it will look like Vim is hung. If you press the CTRL-Q key, then everything will be back to normal.

You can turn off the terminal driver flow control using the 'stty' command:

```
$ stty -ixon -ixoff
```

or, you can change the keys used for the terminal flow control, using the following commands:

```
$ stty stop <char>
$ stty start <char>
```

32.2. I am seeing weird screen update problems in Vim. What can I do to solve this screen/display update problems?

You have to use a proper terminal emulator like xterm with correct TERM settings (TERM=xterm) and a correct terminfo/termcap file.
For more information, read

```
:help 'term'
```

32.3. I am using the terminal/console version of Vim. In insertmode, When I press the backspace key, the character before the cursor is not erased. How do I configure Vim to do this?

You have to make sure that Vim gets the correct keycode for the backpspace key. You can try using the command:

```
:fixdel
```

Make sure the TERM environment variable is set to the correct terminal name. You can try using the 'stty' command:

```
$ stty erase ^H
```

where, you have to enter the ^H character by pressing the CTRL-V key and then the CTRL-H key.

For more information, read

```
:help :fixdel
:help Linux-backspace
:help NetBSD-backspace
```

32.4. I am using Vim in a xterm. When I quit Vim, the screen contents are restored back to the original contents. How do I disable this?

The xterm has a capability called "alternate screen". If this capability is present, vim switches to that alternate screen upon startup and back on exit, thus restoring the original screen contents. To disable this feature, add the following line to your .vimrc file:

```
:set t_ti= t_te=
```

For more information, read

```
:help restorescreen
:help xterm-screens
```

32.5. When I start Vim, it takes quite a few seconds to start. How do I minimize the startup time?

This may be related to Vim opening the X display for setting the xterm title and using the X clipboard. Make sure the DISPLAY variable is set to point to the correct host. Try using the command line:

```
$ vim -X
```

This will prevent Vim from opening the X display. With this command-line option, the X clipboard cannot be used and also Vim will not be able to change the xterm title.

You can also set the 'clipboard' option to

```
:set clipboard=exclude:.*
```

This has the same effect as using the -X command-line argument.

For more information, read

```
:help -X
:help 'clipboard'
```

32.6. How can I make the cursor in gvim in unix stop blinking?

You can modify the 'guicursor' option, to stop the cursor from blinking. For example:

```
:set guicursor=a:blinkon0
```

For more information, read

```
:help 'guicursor'
```

32.7. How do I change the menu font on GTK Vim?

You can modify the ~/.gtkrc file to change the menu font on GTK Vim. For example:

```
style "default"
{ font ="smooth09" }
class "*" style "default"
```

The last line changes the font of all widgets.

For more information, read

```
:help gui-gtk
```

32.8. How do I prevent <Ctrl-Z> from suspending Vim?

You can map <Ctrl-Z> to prevent the suspending. Here are some suggestions:

- Make <Ctrl-Z> do nothing:

```
:map <C-Z> <Nop>
```

- Make <Ctrl-Z> start a shell:

```
:map <C-Z> :shell<CR>
```

- Make <Ctrl-Z> give an error message:

```
:map <C-Z> :"suspending disabled<CR>
```

For the last example, the double quote is necessary in order to keep the message on the status line.

32.9. When I kill the xterm running Vim, the Vim process continues to run and takes up a lot of CPU (99%) time. Why is this happening?

When Vim is built with support for Python interface, you will have this problem. This is a known problem with the python thread library and Vim. To solve this problem, use a Vim binary built without the Python interface.

For more information, read

```
:help +python
:help python
```

32.10. How do I get the Vim syntax highlighting to work in a Unix terminal?

The easiest and simplest way to get Vim syntax highlighting is to use the GUI version of Vim (GVIM). To get syntax highlighting to work in the console/terminal version of Vim, you have to run a terminal emulator (like Xfree86 xterm or rxvt or dtterm) that supports color. Note that if a terminal emulator supports changing the background and foreground colors, that does not mean that it also supports ANSI escape sequences for changing the color. You can download the latest version of Xfree86 xterm from http://dickey.his.com/xterm/xterm.html. You can download the latest version of rxvt from http://www.rxvt.org. You have to install the terminfo/termcap file that supports colors for the terminal emulator. Also, set the TERM environment variable to the correct name of the term that supports colors.

You can use the colortest.vim script supplied with the Vim runtime package to test the color setup. To use this script, follow these steps:

```
:e $VIMRUNTIME/syntax/colortest.vim
:source %
```

For more information, read

```
:help 06.2
:help terminal-colors
:help termcap-colors
:help startup-terminal
:help xterm-color
:help colortest.vim
```

===

SECTION 33 - VIM ON MS-WINDOWS

33.1. In MS-Windows, CTRL-V doesn't start the blockwise visual mode. What happened?

The mswin.vim script provides key mappings and options to make Vim behave like a MS-Windows application. One of the keys mapped is CTRL-V which is used for pasting text in MS-Windows applications. This will disable the use of CTRL-V to start the blockwise visual mode. The mswin.vim script maps CTRL-Q for staring the blockwise visual mode. So you can use CTRL-Q instead of CTRL-V.

For more information, read

```
    :help CTRL-V
    :help CTRl-V-alternative
    :help CTRL-Q
    :help 10.5
```

33.2. When I press the CTRL-Y key, it acts like the CTRL-R key. How do I configure Vim to treat CTRL-Y as CTRL-Y?

The mapping of the CTRL-Y key to the CTRL-R key is done by the mswin.vim script. The mswin.vim script maps CTRL-Y to make Vim behave like a standard MS-Windows application. This is explained in ":help CTRL-Y". You can either comment out the line in mswin.vim that maps the CTRL-Y key or you can remove the line in your .vimrc file that sources the mswin.vim script.

33.3. How do I start GUI Vim in a maximized window always?

You can use the "simalt" command to maximize the Vim window. You can use the GUIEnter autocmd to maximize the Vim window on startup:

```
    autocmd GUIEnter * simalt ~x
```

For more information, read

```
    :help :simalt
    :help GUIEnter
    :help gui-win32-maximized
```

33.4. After doing some editing operations, Vim freezes. The cursor becomes an empty rectangle. I am not able enter any characters. What is happening?

Most probably, you used the mouse wheel to scroll the text in Vim. There is a known problem in using intellimouse mouse wheel with Vim. To avoid this problem, disable Universal scrolling support for Vim.

For more information, read

```
    :help intellimouse-wheel-problems
```

33.5. I am using Windows XP, the display speed of maximized GVim is very slow. What can I do to speed the display updates?

This may be due to the fact that you have enabled 'Smooth edges of screen fonts' in the display properties. Try turning off font smoothing or try changing the smoothing method to "Standard".

33.6. What are the recommended settings for using Vim with cygwin?

You may want to set the following shell related Vim settings:

```
    :set shellcmdflag=-c
    :set shellquote=
    :set shellslash          " Use the forward slash for expansion.
```

```
:set shellxquote=\"
:set shell=d:\cygwin\bin\bash.exe " Use the bash shell
:set shellpipe=2>&1| tee
:set shellredir=>%s 2>&1
```

33.7. I am trying to use GNU diff with Vim diff mode. When I run the diff from command line, it works. When I try to use the diff with Vim it doesn't work. What should I do now?

There is a problem with using GNU diff with Vim. You can try using the GNU diff.exe built by Ron Aaron from the following link:

    http://www.mossbayeng.com/~ron/vim/builds.html

33.8. Is it possible to use Vim as an external editor for MS-Windows Outlook email client?

You can use the "cubiclevim" COM Add-In to use Vim as an external editor for MS-Windows Outlook email client. Visit the following URL for more information:

    http://sourceforge.net/projects/cubiclevim

Note that currently this works only with MS-Office 2000 and XP.

33.9. I am using Vim to edit HTML files. How do I start internet explorer with the current file to preview the HTML file?

You can use the following command:

```
:!start c:\progra~1\intern~1\iexplore.exe file://%:p<CR>
```

33.10. I would like to use Vim with Microsoft Visual Studio. How do I do this?

You have to download and use the OLE version of Vim (for example: gvim61ole.zip). This file also contains instructions on how to use Vim with Visual Studio.

For more information, read

    :help MSVisualStudio

33.11. Where do I place the _vimrc and _gvimrc files?

You can place the _vimrc and _gvimrc files under the directory pointed to by the VIM environment variable. If you are sharing this system with other users, then you can place the files in a directory and set the HOME environment variable to this directory.

For more information, read

    :help $HOME-use

```
    :help _vimrc
```

33.12. Everytime I save a file, Vim warns about the file being changed
       outside of Vim. Why?

If you get the following warning message, everytime you save a file:

```
    WARNING: The file has been changed since reading it!!!
    Do you really want to write to it (y/n)?
```

then this problem could be related to a bug in MS-Windows on the day daylight saving time starts. Vim remembers the timestamp of the file after it was written. Just before the next write the timestamp is obtained again to check if the file was changed outside of Vim. This works correctly, except on the day daylight saving time starts.

This problem will go away the next day after the day the daylight saving time starts.

For more information, read

```
    :help W11
```

===

SECTION 34 - PRINTING

34.1. How do I print a file along with line numbers for all the lines?

You can set the 'printoptions' option and use the ":hardcopy" command to print your file:

```
    :set printoptions=number:y
    :hardcopy
```

For more information, read

```
    :help 'printoptions'
    :help :hardcopy
```

34.2. How do I print a file with the Vim syntax highlighting colors?

You can use the ":hardcopy" command to print a file with the Vim syntax highlighting colors. You can also convert your file to a HTML file using the 2html.vim script and print the HTML file.

For more information, read

```
    :help syntax-printing
    :help 2html.vim
    :help :hardcopy
    :help printing
```

===

SECTION 35 - BUILDING VIM FROM SOURCE

35.1. How do I build Vim from the sources on a Unix system?

For a Unix system, follow these steps to build Vim from the sources:

- Download the source and run-time files archive (vim-##.tar.bz2) from the ftp://ftp.vim.org/pub/vim/unix directory.
- Extract the archive using the bzip2 and tar utilities using the command:

```
        $ bunzip2 -c <filename> | tar -xf -
```

- Run the 'make' command to configure and build Vim with the default configuration.
- Run 'make install' command to install Vim in the default directory.

To enable/disable various Vim features, before running the 'make' command you can run the 'configure' command with different flags to include/exclude the various Vim features. To list all the available options for the 'configure' command, use:

```
    $ configure -help
```

For more information, read

```
    :help install
```

35.2. How do I install Vim in my home directory or a directory other than the default installation directory in Unix?

To install Vim in a directory other than the default installation directory, you have to specify the directory using the --prefix option while running the configure script.

```
    $ ./configure --prefix=/users/xyz
```

You can enable/disable various Vim feature by supplying different arguments to the configure script. For more information about all these options, run

```
    $ ./configure --help
```

For more information, read

```
    :help install-home
    :help install
```

35.3. How do I build Vim from the sources on a MS-Windows system?

For a MS-Windows system, Vim can be built using either the Visual C++ compiler or the Borland C++ compiler or the Ming GCC compiler or the cygwin gcc compiler. Follow these steps to build Vim from the sources for MS-Windows:

- Download the source (vim##src.zip), runtime (vim##rt.zip) and the extra (vim-##-extra.tar.gz) archives from the ftp://ftp.vim.org/pub/vim/pc directory.
- Extract the archives into a directory (for example, c:\vimsrc)
- Depending on the installed compiler, you can use the corresponding makefile to build the Vim sources. For Visual C++ use the Make_mvc.mak makefile, for borland C++ use the Make_bc5.mak makefile, for ming GCC use the Make_ming.mak makefile, for cygwin gcc use the Make_cyg.mak makefile.

Depending on whether you want to build the GUI version of Vim or the console version of Vim, you have to pass different arguments to the makefiles. After successfully building the sources, you can copy the vim.exe or gvim.exe file to the desired directory along with the files from the runtime archive.

You can visit the following site for extensive information about building Vim on a MS-Windows system using the various compilers:

    http://mywebpage.netscape.com/SharpPeople/vim/howto/index.html

For more information, read

    :help install

35.4. The Vim help, syntax, indent files are missing from my Vim installation. How do I install these files?

The Vim help, syntax, indent and other runtime files are part of the Vim runtime package. You need to download and install the Vim runtime package. For example, for MS-Windows, the name of the Vim 6.1 runtime package is vim61rt.zip.

For more information, read

    :help install

35.5. I have built Vim from the source and installed the Vim package using "make install". Do I need to keep the Vim source directory?

No. Once you have built and installed Vim in some directory other than the original source directory (for example, /usr/bin or /usr/local/bin), then you can remove the source directory.

35.6. How do I determine the Vim features which are enabled at compile time?

You can use the ":version" command to determine the Vim features that are enabled at compile time. The features that are enabled will be prefixed with a "+". The features that are not enabled will be prefixed with a "-".

If you want to test for a feature in a script, you can use the has() function:

```
    if has("menu")
        " Set up some menus
```

```
    endif
```

For more information, read

```
    :help :version
    :help +feature-list
    :help has()
```

35.7. Can I build Vim without the GUI support?

Yes. You can build Vim by optionally enabling/disabling many of the features including GUI.

For more information, read

```
    :help install
```

35.8. When building Vim on a Unix system, I am getting "undefined reference to term_set_winsize' error. How do I resolve this error?

You will get this error when the build process is not able to locate the termlib, termcap or ncurses library. You have to install the ncurses-dev package to resolve this error.

35.9. Vim configure keeps complaining about the lack of gtk-config while trying to use GTK 2.03. This is correct, since in GTK 2 they moved to using the generic pkg-config. I can get pkg-config to list the various includes and libs for gtk, but for some reason the configure script still isn't picking this up.

Use the following shell script named gtk-config:

```
    #!/bin/sh
    pkg-config gtk+-2.0 $1 $2
```

===

SECTION 36 - VARIOUS

36.1. How do I edit binary files with Vim?

You can set the following options to edit binary files in Vim:

```
    :set binary
    :set display=uhex
```

You can also use the "-b" command-line option to edit a binary file:

```
    $ vim -b <binary_file_name>
```

You can also use the xxd utility (part of the Vim distribution) to edit binary files.

For more information, read

```
:help 23.4
:help edit-binary
:help hex-editing
:help -b
:help 'binary'
:help 'endofline'
:help 'display'
```

36.2. How do I disable the visual error flash and the error beep?

You can disable both the visual error flash and the error beep using the following command:

```
:set visualbell t_vb=
```

For more information, read

```
:help 'visualbell'
:help 'errorbells'
:help t_vb
```

36.3. How do I display the ascii value of a character displayed in a buffer?

You can use the 'ga' command to display the ascii value of a displayed character.

For more information, read

```
:help ga
:help :ascii
```

36.4. Can I use zero as a count for a Vim command?

You cannot use zero as a count for a Vim command, as "0" is a command on its own, moving to the first column of the line.

For more information, read

```
:help 0
:help count
```

36.5. How do I disable the Vim welcome screen?

You can disable the Vim welcome screen, by adding the 'I' flag to the 'shortmess' option:

```
:set shortmess+=I
```

For more information, read

```
:help :intro
```

```
:help 'shortmess'
```

36.6. How do I avoid the "hit enter to continue" prompt?

Vim will prompt you with the "hit enter to continue" prompt, if there are some messages on the screen for you to read and the screen is about to be redrawn. You can add the 'T' flag to the 'shortmess' option to truncate all messages. This will help in avoiding the hit-enter prompt:

```
:set shortmess+=T
```

You can also increase the command height by setting the 'cmdheight' option:

```
:set cmdheight=2
```

For more information, read

```
:help hit-enter
:help avoid-hit-enter
:help 'shortmess'
:help 'cmdheight'
```

36.7. How do I invoke Vim from command line to run a group of commands on a group of files?

There are several ways to invoke Vim from command line to run a group of commands on a group of files. You can use a set of "-c" command line options to specify a group of commands:

```
$ vim -c "<ex_command_1>" -c "<ex_command_2>" *.txt
```

Each of the ex-command specified with the "-c" command line option is executed one by one sequentially. You can also use a single "-c" command line option and the "|" character to separate the ex commands:

```
$ vim -c "<ex_command_1> | <ex_command_2>" *.txt
```

In the above command, if an ex command fails, then all the remaining ex commands will not be executed.

For example, to replace "ABC" with "DEF" in a file from the command-line, you can use the following command:

```
$ vim -c "%s/ABC/DEF/ge | update" myfile.txt
```

To replace "ABC" with "DEF" in multiple files from the command-line, you can use the following command:

```
$ vim -c "argdo %s/ABC/DEF/ge | update" *.txt
```

You can store the group of commands into a file and use the "-s" command line option to run the commands on a set of files. For example, if the group of commands are stored in the file mycmds.txt, then you can use the following command:

```
$ vim -s mycmds.txt *.pl
```

For more information, read

```
:help -c
:help -s
```

36.8. How do I use a normal mode command from insert mode without leaving the insert mode?

You can use a normal command from insert mode, without leaving the insert mode, by first pressing the CTRL-O key and then follow that with a single normal mode command.

To execute more than one normal mode command, press the CTRL-L key, followed by any number of normal mode commands and then press <Esc> to get back to the insert mode.

For more information, read

```
:help i_CTRL-O
:help i_CTRL-L
```

36.9. How do I start Vim in insert mode?

You can start Vim in insert mode using the ":startinsert" ex command.

```
$ vim +startinsert myfile.txt
```

The above command will open the file "myfile.txt" and start insert mode with the cursor in front of the first character on the first line. To open the file and start appending after the last character on the last line, you can use the following command:

```
$ vim + +startinsert! myfile.txt
```

For more information, read

```
:help :startinsert
```

===

SECTION 37 - UNICODE
Author: Tony Mechelynck <antoine.mechelynck AT belgacom.net>

37.1. Is it possible to create Unicode files using Vim?

Yes. It may be more or less complicated depending on the keyboard and fonts available to you, but it is always possible to encode any possible Unicode codepoint (and some illegal ones) into a file. To create a Unicode file using Vim, you should have compiled Vim with the "+multi_byte" compile-time option. You can get more information about Unicode from the following sites:

    http://www.unicode.org

    http://www.cl.cam.ac.uk/~mgk25/unicode.html

For more information, read

    :help multibyte
    :help usr_45.txt

37.2. Which Vim settings are particularly important for editing Unicode files?

The most important are the various "encoding" options, i.e., 'encoding', 'fileencoding', 'fileencodings' and 'termencoding'. The boolean option 'bomb' is also significant.

For more information, read

    :help 'encoding'
    :help 'fileencoding'
    :help 'fileencodings'
    :help 'termencoding'
    :help 'bomb'

37.3. What is the 'encoding' option?

Basically, the 'encoding' option defines how Vim will represent your data internally. However, all Unicode encodings are represented internally as utf-8 and converted (if necessary) when reading and writing.

For more information, read

    :help 'encoding'

37.4. How does Vim name the various Unicode encodings?

Utf-8 is called utf-8 or utf8; utf-16 is called ucs-2 or ucs2; utf-32 is called ucs-4 or ucs4. Also, you may specify endianness (except for utf-8 which does not vary for endianness) by appending le for little-endian or be for big-endian. If you create a file with an encoding of ucs-2 or ucs-4 without specifying endianness, Vim will use what is typical of your machine.

For more information, read

    :help encoding-names
    :help encoding-values
    :help encoding-table

37.5. How does Vim specify the presence or absence of a byte-order mark?

When reading a file, if the 'fileencodings' option includes "ucs-bom", Vim will check for a byte-order mark. When writing a file, if the 'bomb' option is set, Vim will write a byte-order mark on files whose encoding warrants it.

For more information, read

```
:help 'fileencodings'
:help 'bomb'
```

37.6. What is the 'fileencoding' option?

The 'fileencoding' option defines the particular encoding which Vim will use to write a file. If empty, then the value of the 'encoding' option is the default.

For more information, read

```
:help 'fileencoding'
```

37.7. What is the 'fileencodings' option?

The 'fileencodings' option defines the heuristics used by Vim when opening an existing file. It is a comma separated list of encodings. A special name, "ucs-bom" is used to indicate that Vim should check for the presence of a byte-order mark; however, it will not be recognised if it comes after "utf-8". Normally, "ucs-bom" (if present) should be first in the list.

When Vim opens a file, it checks it against the encodings listed in 'fileencodings'. The first one that matches is used. If there is no match, then Vim sets 'fileencoding' to the null string, i.e., the value of 'encoding' will be used.

For more information, read

```
:help 'fileencodings'
:help 'encoding'
```

37.8. What is the 'termencoding' option?

The 'termencoding' option defines how your keyboard encodes the data you type. If empty, Vim assumes that it has the same value as 'encoding'. Usually it should be set to something that matches your locale.

For more information, read

```
:help 'termencoding'
:help locale
```

37.9. What is the 'bomb' option?

When reading a file with "ucs-bom" present in the 'fileencodings' option, Vim will set the 'bomb' option on or off depending on the presence or absence of a byte-order mark at the start of the file. When writing, Vim will write a byte-order mark if the 'bomb' option is set. You may set or unset it manually do make Vim write, or not write, the b.o.m.

For more information, read

```
    :help 'bomb'
```

37.10. Where can I find an example of a typical use of all these options?

There is a "tip", with explains them in different words with an example, at
http://vim.sourceforge.net/tip_view.php?tip_id=246 .

37.11. How can I insert Unicode characters into a file using Vim?

Several methods are available:

- Characters present on your keyboard can be typed in the usual way, even those which require a "dead-key" prefix, like (for instance) the circumflex on French keyboards.
- Characters for which a digraph is defined can be typed as two characters prefixed by <Ctrl-K>.
- If you have set the 'digraph' option, you can enter the characters for which a digrph is defined as <char1><BS><char2>.
- Any character can be entered by using a <Ctrl-V> prefix (or <Ctrl-Q> if <Ctrl-V> is remapped to paste from the clipboard).

For more information, read

```
    :help digraphs
    :help 'digraph'
    :help i_CTRL-V_digit
```

37.12. How can I know which digraphs are defined and for which characters?

First set the 'encoding' option properly (for instance, to utf-8), then use
the :digraphs command to list the currently defined digraphs.

For more information, read

```
    :help :digraphs
    :help 'encoding'
```

===

Current Maintainer: Yegappan Lakshmanan
Last updated on: 2 January 2005

Generated by vim2html.pl 3.2 on Wed Oct 19 01:54:34 2005
