# Supplementary material for: Simple tools for assembling and searching high-density picolitre pyrophosphate sequence data
Source: Source Code Biol Med. 2008 Apr 18;3:5. doi: 10.1186/1751-0473-3-5 (PMC2374781; doi:10.1186/1751-0473-3-5)
Supplement: Additional file 3 — Supplementary tables. Supplementary tables containing sample output from several of the tools. [file 1751-0473-3-5-S3.pdf]

Parker N.J. and A.G. Parker (2008) **Simple tools for assembling and searching high-density picolitre pyrophosphate sequence data.** *Source Code for Biology and Medicine.*

Supplementary Table 1. Section of the output from `re`

Section of output showing the matches falling to zero where a single erroneous base has been inserted by the assembler, marked in red. For column descriptions see the section on `re` in main paper.

2761	T	GTTGTGGGTTAAAAAATACTTCCTCACCTTCATAA	C	43	100%	43	100%
2762	G	TTGTGGGTTAAAAAATACTTCCTCACCTTCATAAC	T	43	97%	44	100%
2763	T	TGTGGGTTAAAAAATACTTCCTCACCTTCATAACT	G	44	100%	44	100%
2764	T	GTGGGTTAAAAAATACTTCCTCACCTTCATAACTG	T	44	100%	45	100%
2765	G	TGGGTTAAAAAATACTTCCTCACCTTCATAACTGT	T	45	100%	45	97%
2766	T	GGGTTAAAAAATACTTCCTCACCTTCATAACTGTT	G	45	97%	44	95%
2767	G	GGTTAAAAAATACTTCCTCACCTTCATAACTGTTG	C	44	100%	45	100%
2768	G	GTTAAAAAATACTTCCTCACCTTCATAACTGTTGC	**C**	45	100%	0	0%
2769	G	TTAAAAAATACTTCCTCACCTTCATAACTGTTGCC	T	0	0%	0	0%
2770	T	TAAAAAATACTTCCTCACCTTCATAACTGTTGCCT	T	0	0%	0	0%
2771	T	AAAAAATACTTCCTCACCTTCATAACTGTTGCCTT	T	0	0%	0	0%
2772	A	AAAAATACTTCCTCACCTTCATAACTGTTGCCTTT	C	0	0%	0	0%
2773	A	AAAATACTTCCTCACCTTCATAACTGTTGCCTTTC	C	0	0%	0	0%
2774	A	AAATACTTCCTCACCTTCATAACTGTTGCCTTTCC	C	0	0%	0	0%

Supplementary Table 2. Output from `fr`

Output showing the fragments matching a search string. The original 454 read identification is prefixed to the sequence preceded by either > to indicate a forward read or < to indicate that the read has been reverse complemented. The read length and full read are shown.

```
GGTAT.TTAGGTGTG.TGTGAACAGGATTG.TAACAAAA.TAACAC.CCCAT

>455_081966_0306_2620     length=110    ATTTTCTATCAGGCTCAGGAGGAGGCGGTTGTGGTATATTAGGTGTGCTGTGAACAGGATTGCTAACAAAAGTAACACCCCCATTGTTAATTTGAAAATCTCTATTATTA
>455_091256_0910_3654     length=113    TTGTGGTATATTAGGTGTGCTGTGAACAGGATTGCTAACAAAAGTAACACCCCCATTGTTAATTTGAAAATCTCTATTATTATCTGTTAATACTGTATATTCAAATTCGAACG
>455_102147_1979_2910     length=110    ATTTGGTGGATTTTCATCAGGCTCAGGAGGAGGCGGTTGTGGTATATTAGGTGTGCTGTGAACAGGATTGCTAACAAAAGTAACACCCCCATTGTTAATTTGAAAATCTC
>455_109194_1169_1583     length=111    GGTGGATTTTCATCAGGCTCAGGAGGAGGCGGTTGTGGTATATTAGGTGTGCTGTGAACAGGATTGCTAACAAAAGTAACACCCCCATTGTTAATTTGAAAATCTCTATTA
>455_142673_0764_1798     length=114    GTTGTGGTATATTAGGTGTGCTGTGAACAGGATTGCTAACAAAAGTAACACCCCCATTGTTAATTTGAAAATCTCTATTATTATCTGTTAATACTGTATATTCAAATTCGAACG
>455_163274_1640_3114     length=109    TATCATTTGGTGGATTTTCATCAGGCTCAGGAGGAGGCGGTTGTGGTATATTAGGTGTGCTGTGAACAGGATTGCTAACAAAAGTAACACCCCCATTGTTAATTTGAAA
>455_163368_0496_3662     length=107    TCATCAGGCTCAGGAGGAGGCGGTTGTGGTATATTAGGTGTGCTGTGAACAGGATTGCTAACAAAAGTAACACCCCCATTGTTAATTTGAAAATCTCTATTATTATC
>456_006368_3425_2092     length=109    AGGAGGAGGCGGTTGTGGTATATTAGGTGTGCTGTGAACAGGATTGCTAACAAAAGTAACACCCCCATTGTTAATTTGAAAATCTCTATTATTATCTGTTAATACTGTC
>456_032254_3817_2020     length=110    TTTTCATCAGGCTCAGGAGGAGGCGGTTGTGGTATATTAGGTGTGCTGTGAACAGGATTGCTAACAAAAGTAACACCCCCATTGTTAATTTGAAAATCTCTATTATTATC
>456_049864_3166_3323     length=94     GTTGTGGTATATTAGGTGTGCTGTGAACAGGATTGCTAACAAAAGTAACACCCCCATTGTTAATTTGAAAATCTCTATTATTATCTGTTAATAC
>456_198291_3269_1432     length=105    AGGAGGAGGCGGTTGTGGTATATTAGGTGTGCTGTGAACAGGATTGCTAACAAAAGTAACACCCCCATTGTTAATTTGAAAATCTCTATTATTATCTGTTAATAC
>456_214781_2425_2138     length=110    TATCATTTGGTGGATTTTCATCAGGCTCAGGAGGAGGCGGTTGTGGTATATTAGGTGTGCTGTGAACAGGATTGCTAACAAAAGTAACACCCCCATTGTTAATTTGAAAA
<455_051341_1534_1263     length=111    GGTTGGATTTTCATCAGGCTCAGGAGGAGGCGGTTGTGGTATATTAGGTGTGCTGTGAACAGGATTGCTAACAAAAGTAACACCCCCATTGTTAATTTGAAAATCTCTATT
<455_064059_1110_1651     length=106    GTAATGTATCATTTGGTGGATTTTCTATCAGGCTCAGGAGGGAGGCGGTTGTGGTATATTAGGTGTGCTGTGAACAGGATTGCTAACAAAAGTAACACCCCCATTG
<455_112354_1566_2247     length=97     TTTGGTGGATTTCATCAGGCTCAGGAGGAGGCGGTTGTGGTATATTAGGTGTGCTGTGAACAGGATTGCTAACAAAAGTAACACCCCCATTGTTAAT
<455_118761_0209_1715     length=112    GGAGGCGGTTGTGGTATATTAGGTGTGCTGTGAACAGGATTGCTAACAAAAGTAACACCCCCATTGTTAATTTGAAAATCTCTATTATTATCTGTTAATACTGTATATTCAA
<455_126853_0746_3516     length=111    GGTATATTAGGTGTGCTGTGAACAGGATTGCTAACAAAAGTAACACCCCCATTGTTAATTTGAAAATCTCTATTATTATCTGTTAATACTGTATATTCAAATTCGAACGAG
<455_137207_1805_0789     length=111    GGCGGTTGTGGTATATTAGGTGTGCTGTGAACAGGATTGCTAACAAAAGTAACACCCCCATTGTTAATTTGAAAATCTCTATTATTATCTGTTAATACTGTATATTCAAAT
<455_165081_1571_0423     length=106    TCAGGCTCAGGAGGAGGCGGTTGTGGTATATTAGGTGTGCTGTGAACAGGATTGCTAACAAAAGTAACACCCCCATTGTTTAATTTGAAAATCTCTATTATTATCT
<455_170174_1574_3732     length=112    TCAGGCTCAGGAGGAGGCGGTTGTGGTATTTTAGGTGTGTTGTGAACAGGATTGTTAACAAAATTAACACTCCCATTGTTAATTTGAAAATCTCTATTATTATCTGTTAATA
<455_204722_0874_3637     length=106    GAATGTATCATTTGGTGGATTTTCATCAGGCTCAGGAGGAGGCGGTTGTGGTATATTAGGTGTGCTGTGAACAGGATTGCTAACAAAAGTAACACCCCCATTGTTA
<455_212654_1752_0837     length=110    TTTGGTGGATTTTCATCAGGCTCAGGAGGAGGCGGTTGTGGTATATTAGGTGTGCTGTGAACAGGATTGCTAACAAAAGTAACACCCCCATTGTTAATTTGAAAATCTCT
<455_220591_1075_1897     length=104    GGAGGAGGCGGTTGTGGTATATTAGGTGTGCTGTGAACAGGATTGCTAACAAAAGTAACACCCCCATTGTTAATTTGAAATCTCTATTATTATCTGTTTAATAC
<455_241012_0454_3789     length=101    TTTGGTGGATTTTCATCAGGCTCAGGAGGAGGCGGTTGTGGTATATTAGGTGTGCTGTGAACAGGATTGCTAACAAAAGTAACACCCCCATTGTTAATTTG
<455_243583_0878_1829     length=115    GGCGGTTGTGGTATATTAGGTGTGCTGTGAACAGGATTGCTAACAAAAGTAACACCCCCATTGTTAATTTGAAAATCTCTATTATTATTCTGTTAATACTGTATTATTCAAATTC
<455_260875_1834_1653     length=104    TCAGGCTCAGGAGGAGGCGGTTGTGGTATATTAGGTGTGCTGTGAACAGGATTGCTAACAAAAGTAACACCCCCATTGTTAATTTGAAATCTCTATTATTATCT
<455_267806_0561_3895     length=113    CGGTTGTGGTATATTAGGTGTGCTGTGAACAGGATTGCTAACAAAAGTAACACCCCCATTGTTAATTTGAAATCTCTATTATTATCTGTTAATACTGTATATTCAAATTCGAA
<455_283695_0138_0865     length=107    GAATGTATCATTTGGTGGATTTTCATCAGGCTCAGGAGGAGGCGGTTGTGGTATATTAGGTGTGCTGTGAACAGGATTGCTAACAAAAGTAACACCCCCATTGTTAA
<455_306391_1904_3813     length=111    GGATTTTCATCAGGCTCAGGAGGAGGCGGTTGTGGTATATTAGGTGTGCTGTGAACAGGATTGCTAACAAAAGTAACACCCCCATTGTTTAATTTGAAAATCTCTATTATT
<456_008779_3968_0646     length=112    GGATTTTCATCAGGCTCAGGAGGAGGCGGTTGTGGTATATTAGGTGTGCTGTGAACAGGATTGCTAACAAAAGTAACACCCCCATTGTTTAATTTGAAAATCTCTATTATTA
<456_000300_3977_1786     length=109    GGTGGTATATTAGGTGTGCTGTGAACAGGATTGCTAACAAAAGTAACACCCCCATTGTTAATTTGAAATCTCTATTATTATCTGTTAATACTGTATATTCAAATTCGAA
<456_062802_2557_0874     length=105    TCAGGCTCAGGAGGAGCGGTTGTGGTATATTAGGTGTGCTGTGAACAGGATTGCTAACAAAAGTAACACCCCCATTGTTTAATTTGAAAANCTCTATTATTATCT
<456_111500_3039_1880     length=111    GATTTGGTGGATTTTCATCAGGCTCAGGAGGAGGCGGTTGTGGTATATTAGGTGTGCTGTGAACAGGATTGCTAACAAAAGTAACACCCCCATTGTTAATTTGAAAATCTC
<456_135531_3675_3667     length=103    GAATGTATCATTTGGTGGATTTTCATCAGGCTCAGGAGGAGGCGGTTGTGGTATATTAGGTGTGCTGTGAACAGGATTGCTAACAAAAGTAACACCCCCATTG
<456_154563_3773_4023     length=105    TCTGGTGGATTTTCATCAGGCTCAGGAGGAGGCGGTTGTGGTATTTTAGGTGTGTTGTGAACAGGATTGTTAACAAAATTAACACTCCCATTGTTAATTTGAAAA
<456_163293_3012_2113     length=105    GGAGGAGGCGGTTGTGGTATATTAGGTGTGCTGTGAACAGGATTGCTAACAAAAGTAACACCCCCATTGTTTAATTTGAAATCTCTATTATTATCTGTTAATACT
<456_181082_2380_0357     length=107    TCTGGTGGATTTTCATCAGGCTCAGGAGGAGGCGGTTGTGGTATTTTAGGTGTGTTGTGAACAGGATTGTTAACAAAATTAACACTCCCATTTGTTTAATTTGAAAA
<456_185166_2201_3372     length=113    GGTATATTAGGTGTGCTGTGAACAGGATTGCTAACAAAAGTAACACCCCCATTGTTAATTTTGAAAATCTCTATTATTATCTGTTAATACTGTATATTCAAATTCGAACGAGG
<456_194561_3006_3956     length=105    GGAGGAGGCGGTTGTGGTATATTAGGTGTGCTGTGAACAGGATTGCTAACAAAAGTAACACCCCCATTGTTTAATTTGAAATCTCTATTATTATCTGTTAATACT
<456_223114_3669_3202     length=117    GGTATTTTAGGTGTGTTGTGAACAGGATTGTTAACAAAATTAACACTCCCATTGTTAATTTGAAAATCTCTATTATTATCTGTTAATACTGAATATTCAAATTCGAACGAGGTGAAG
<456_260098_3313_1763     length=110    GGACTCAGGAGGAGGCGGTTNTGGTATATTAGGTGTGCTGTGAACAGGATTGCTAACAAAAGTAACACCCCCATTGTTAATTTGAAATCTCTATTATTATCTGTTAATAC
<456_261108_2294_1263     length=103    GAATGTATCATTTGGTGGATTTTCATCAGGCTCAGGAGGAGGCGGTTGTGGTATATTAGGTGTGCTGTGAACAGGATTGCTAACAAAAGTAACACCCCCATTG
<456_279253_3657_2596     length=112    GTGGTATATTAGGTGTGCTGTGAACAGGATTGCTAACAAAAGTAACACCCCCATTGTTAATTTGAAAATCTCTATTATTATCTGTTAATACTGTATATTCAAATTCGAACGA
```

Parker N.J. and A.G. Parker (2008) **Simple tools for assembling and searching high-density picolitre pyrophosphate sequence data.** *Source Code for Biology and Medicine.*

Supplementary Table 3. Output from `fr2`

Output from `fr2` showing the presence of a putative polymorphism in the SGHV. `fr2` left truncates the reads to the beginning of *search_string* before displaying them to allow the sequences to be compared. The forward and complemented reads are shown separately, each sorted into alphabetical order. The last four entries in the reverse reads have an alternative sequence

```
GGTATTTTAGGTGTGTTGTGAACAGGATTGTTAACAAAATTAACACTCCCAT

GGTAT.TTAGGTGTG.TGTGAACAGGATTG.TAACAAAA.TAACAC.CCCAT

>
GGTATATTAGGTGTGCTGTGAACAGGATTGCTAACAAAAGTAACACCCCCATTGTTAATTTGAAA
GGTATATTAGGTGTGCTGTGAACAGGATTGCTAACAAAAGTAACACCCCCATTGTTAATTTGAAAA
GGTATATTAGGTGTGCTGTGAACAGGATTGCTAACAAAAGTAACACCCCCATTGTTAATTTGAAAATCTC
GGTATATTAGGTGTGCTGTGAACAGGATTGCTAACAAAAGTAACACCCCCATTGTTAATTTGAAAATCTCTATTA
GGTATATTAGGTGTGCTGTGAACAGGATTGCTAACAAAAGTAACACCCCCATTGTTAATTTGAAAATCTCTATTATTA
GGTATATTAGGTGTGCTGTGAACAGGATTGCTAACAAAAGTAACACCCCCATTGTTAATTTGAAAATCTCTATTATTATC
GGTATATTAGGTGTGCTGTGAACAGGATTGCTAACAAAAGTAACACCCCCATTGTTAATTTGAAAATCTCTATTATTATC
GGTATATTAGGTGTGCTGTGAACAGGATTGCTAACAAAAGTAACACCCCCATTGTTAATTTGAAAATCTCTATTATTATCTGTTAATAC
GGTATATTAGGTGTGCTGTGAACAGGATTGCTAACAAAAGTAACACCCCCATTGTTAATTTGAAAATCTCTATTATTATCTGTTAATAC
GGTATATTAGGTGTGCTGTGAACAGGATTGCTAACAAAAGTAACACCCCCATTGTTAATTTGAAAATCTCTATTATTATCTGTTAATACTGTATATTCAAATTCGAACG
GGTATATTAGGTGTGCTGTGAACAGGATTGCTAACAAAAGTAACACCCCCATTGTTAATTTGAAAATCTCTATTATTATCTGTTAATACTGTATATTCAAATTCGAACG
GGTATATTAGGTGTGCTGTGAACAGGATTGCTAACAAAAGTAACACCCCCATTGTTAATTTGAAAATCTCTATTATTATCTGTTAATACTGTC

<
GGTATATTAGGTGTGCTGTGAACAGGATTGCTAACAAAAGTAACACCCCCATTG
GGTATATTAGGTGTGCTGTGAACAGGATTGCTAACAAAAGTAACACCCCCATTG
GGTATATTAGGTGTGCTGTGAACAGGATTGCTAACAAAAGTAACACCCCCATTG
GGTATATTAGGTGTGCTGTGAACAGGATTGCTAACAAAAGTAACACCCCCATTGTTA
GGTATATTAGGTGTGCTGTGAACAGGATTGCTAACAAAAGTAACACCCCCATTGTTAA
GGTATATTAGGTGTGCTGTGAACAGGATTGCTAACAAAAGTAACACCCCCATTGTTAAT
GGTATATTAGGTGTGCTGTGAACAGGATTGCTAACAAAAGTAACACCCCCATTGTTAATTTG
GGTATATTAGGTGTGCTGTGAACAGGATTGCTAACAAAAGTAACACCCCCATTGTTAATTTGAAAATCTC
GGTATATTAGGTGTGCTGTGAACAGGATTGCTAACAAAAGTAACACCCCCATTGTTAATTTGAAAATCTCT
GGTATATTAGGTGTGCTGTGAACAGGATTGCTAACAAAAGTAACACCCCCATTGTTAATTTGAAAATCTCTATT
GGTATATTAGGTGTGCTGTGAACAGGATTGCTAACAAAAGTAACACCCCCATTGTTAATTTGAAAATCTCTATTATTATCTGTTAATACTGTATATTCAA
GGTATATTAGGTGTGCTGTGAACAGGATTGCTAACAAAAGTAACACCCCCATTGTTAATTTGAAAATCTCTATTATTATCTGTTAATACTGTATATTCAAAT
```

Parker N.J. and A.G. Parker (2008) **Simple tools for assembling and searching high-density picolitre pyrophosphate sequence data.** *Source Code for Biology and Medicine.*

```
GGTATATTAGGTGTGCTGTGAACAGGATTGCTAACAAAAGTAACACCCCCATTGTTAATTTGAAAATCTCTATTATTATCTGTTAATACTGTATATTCAAATTCGAACGA
GGTATATTAGGTGTGCTGTGAACAGGATTGCTAACAAAAGTAACACCCCCATTGTTAATTTGAAAATCTCTATTATTATCTGTTAATACTGTATATTCAAATTCGAACGAG
GGTATATTAGGTGTGCTGTGAACAGGATTGCTAACAAAAGTAACACCCCCATTGTTAATTTGAAAATCTCTATTATTATTCTGTTAATACTGTATTATTCAAATTC
GGTATATTAGGTGTGCTGTGAACAGGATTGCTAACAAAAGTAACACCCCCATTGTTAATTTGAAATCTCTATTATTATCT
GGTATATTAGGTGTGCTGTGAACAGGATTGCTAACAAAAGTAACACCCCCATTGTTAATTTGAAATCTCTATTATTATCTGTTAATAC
GGTATATTAGGTGTGCTGTGAACAGGATTGCTAACAAAAGTAACACCCCCATTGTTAATTTGAAATCTCTATTATTATCTGTTAATACTGTATATTCAAATTCGAA
GGTATATTAGGTGTGCTGTGAACAGGATTGCTAACAAAAGTAACACCCCCATTGTTAATTTGAAATCTCTATTATTATCTGTTAATACTGTATATTCAAATTCGAA
GGTATATTAGGTGTGCTGTGAACAGGATTGCTAACAAAAGTAACACCCCCATTGTTAATTTGAAATCTCTATTATTATCTGTTTAATAC
GGTATATTAGGTGTGCTGTGAACAGGATTGCTAACAAAAGTAACACCCCCATTGTTAATTTTGAAAATCTCTATTATTATCTGTTAATACTGTATATTCAAATTCGAACGAGG
GGTATATTAGGTGTGCTGTGAACAGGATTGCTAACAAAAGTAACACCCCCATTGTTTAATTTGAAAANCTCTATTATTATCT
GGTATATTAGGTGTGCTGTGAACAGGATTGCTAACAAAAGTAACACCCCCATTGTTTAATTTGAAAATCTCTATTATT
GGTATATTAGGTGTGCTGTGAACAGGATTGCTAACAAAAGTAACACCCCCATTGTTTAATTTGAAAATCTCTATTATTA
GGTATATTAGGTGTGCTGTGAACAGGATTGCTAACAAAAGTAACACCCCCATTGTTTAATTTGAAAATCTCTATTATTATCT
GGTATATTAGGTGTGCTGTGAACAGGATTGCTAACAAAAGTAACACCCCCATTGTTTAATTTGAAATCTCTATTATTATCTGTTAATACT
GGTATATTAGGTGTGCTGTGAACAGGATTGCTAACAAAAGTAACACCCCCATTGTTTAATTTGAAATCTCTATTATTATCTGTTAATACT
GGTATTTTAGGTGTGTTGTGAACAGGATTGTTAACAAAATTAACACTCCCATTGTTAATTTGAAAA
GGTATTTTAGGTGTGTTGTGAACAGGATTGTTAACAAAATTAACACTCCCATTGTTAATTTGAAAATCTCTATTATTATCTGTTAATA
GGTATTTTAGGTGTGTTGTGAACAGGATTGTTAACAAAATTAACACTCCCATTGTTAATTTGAAAATCTCTATTATTATCTGTTAATACTGAATATTCAAATTCGAACGAGGTGAAG
GGTATTTTAGGTGTGTTGTGAACAGGATTGTTAACAAAATTAACACTCCCATTTGTTTAATTTGAAAA
```

Supplementary Table 4. Frequency of some repeat sequences from the SGHV

Including some false sequences created by changing a single base in an existing repeat

Repeat		Copies in Sequence	Copies in 454 reads
<p1>	GAATGATTCTGGTATATCAATTGTCCC	1	85
<p2>	GAATAGTTTCAGTACAACAAATCACCC	2	176
<p3>	GAATCGTTTCAGTACAACAAAACACCC	0	6
<p4>	GAATCGTTTCAGTACATCAATTGTCCC	0	2
<p5>	GAATGGTTTCAGTATAACAAATTACCC	2	151
<p6>	GAATGGTTTCAGTACTACAATTGGCCC	1	87
<p7>	GAATGATTCTAGTACTACAATTGACGC	1	95
<p8>	GAATGATTCTAGTACTACAATTGGCCC	1	106
<p9>	AGATCATTTTAATACAATAACTGACCC	1	81
<p10>	AAATGATTCTGGTATATCAATTGTCCC	0	1
<p11>	TAATGATTCTGGTATATCAATTGTCCC	0	2
<p12>	GAATGATTCTAGTATATCAATTGTCCC	3	224
<p13>	GAATGATTCTGGTATATCAATTGACCC	0	5
<p14>	GAATGATTCTGGTATATCAATTGCCCC	0	0
<p15>	GAATGATTCTGGTATATCAATTGGCCC	1	72
<p16>	TAATAGTTTCAGTACAACAAATCACCC	0	1
<p17>	GAATGGTTTCAGTACAACAAATCACCC	2	166
<p18>	GAATAGTTTCAGTACAACAAATCCCCC	0	0
<p19>	GAATAGTTTCAGTACAACAAATCGCCC	0	0
<p20>	TAATGGTTTCAGTATAACAAATTACCC	0	0
<p21>	GAATGGTTACAGTATAACAAATTACCC	0	1
<p22>	AAATGGTTTCAGTACTACAATTGGCCC	0	1
<p23>	GAATGGTTTCAGTACTACAATTAGCCC	0	0
<p24>	GAATGGTTTCAGTACTACAATTTGCCC	0	0
<p25>	TAATCGTTTCAGTACATCAATTGACCC	0	0
<p26>	GAATGGTTTCAGTACATCAATTGACCC	0	0
<p27>	GAATCTTTTCAGTACATCAATTGACCC	0	0
<p28>	GAATCGGTTCAGTACATCAATTGACCC	0	1
<p29>	GAATCGTTTCAGTATATCAATTGACCC	0	0
<p30>	GAATGGTTTCAGTATATCAATTGACCC	1	81
<p31>	GAATAGTTTCAGTATATCAATTGACCC	0	0
<p32>	GAATCGTTTCAGTACATCAATTGTCCC	0	0
<p33>	GAATGGTTTCAGTACATCAATTGTCCC	0	0
<p34>	GGATGATTCTAGTATATCAATTGTCCC	0	0
<p35>	GAATTATTCTAGTATATCAATTGTCCC	0	0
<p36>	GAATGATTCTATTATATCAATTGTCCC	0	0
<p37>	GAATGATTCTAGTATATCAATCGTCCC	0	0
<p38>	GAATGATTCTAGTATATCAATTGACCC	0	0
<p49>	GAATAGTTTCAGTATATTAATTGACCC	1	76
<p50>	GAATAGTTTCAGTATATCAACTGACCC	0	0
<p56>	GAATAGTTTCAGTATATCAATTGGCCC	1	77
<p57>	GAATCGTTTCAGTATATCAATTGGCCC	0	0
<p58>	GAATGGTTTCAGTATATCAATTGGCCC	1	95
<p65>	GAATGATTCTGGTATAACAAATTACCC	2	169
<p66>	GAATGATTCTGGTATATCAATTGTCCT	1	76
<p67>	GAATCGTTTCAGTACATCAATTGACGC	1	75
<p68>	GAATGGTTTCAGTACATCAATTGTACG	0	2

<p76>	GAATGGTTTCAGTACATCAATTGACGC	1	71
<p77>	GAATCTTTTCAGTACATCAATTGACGC	0	1
<p78>	GAATCGTTTCAGTATATCAATTGACGC	1	89
<p79>	GAATGGTTTCAGTATATCAATTGACGC	1	70
<p80>	GAATAGTTTCAGTATATCAATTGACGC	0	11
<p81>	GAATGGTTTCAGTACATCAATTGTACG	0	2
<p82>	GAATGATTCTAGTATATCAATTGACGC	1	79
<p83>	GAATAGTTTCAATATATCAATTGATGC	0	3
<p84>	GAATAGTTTCAGTATATCAACTGACTC	1	90
<p85>	GAATCGTTTCAGTATATCAACTGACTC	1	88
<p86>	GAATGGTTTCAGTATATCAACTGACGC	1	76
<p87>	GAATCGTTTCAGTATATCAATTGGCGC	0	5
<p88>	GAATGGTTTCAGTATATCAATTGGCTC	1	84
<p89>	GAATGGTTTCAGTATATCAATTAGACC	0	3
<p90>	GAATGATTCTAGTACTACAATTGTCCC	1	71
<p91>	GAATGATTTTAATACAATAACTGACCC	1	72
<p92>	GAATATTCTATGTACTAGTACTGTTTT	1	65
<p93>	AGATCATTTTAATATAATAACTGACCC	1	79
<p94>	GAATGATTTTAGTACTACAATTGACCC	1	87
<p95>	GAATAGTTTCAGTATATCAGGTACTGA	1	79
<p96>	AGATCATTTCAGTACAACAAGTTACCC	1	90
<p97>	AAATGATTTTAATATACCAAATGACCC	1	69
<p98>	GAATGATTCTTGTACCTCATATAATAA	1	56
<p99>	GAATAGTTTTAGTACAACAAATCACCC	0	0
		40	

<q1>	GGGGCATCAGAGAAAAGACAGAAATAT	3	221
<q2>	GGGGCATCAGAGAAAAGACGTAAATAT	2	159
<q3>	GGGACATTATAGAAAAGACAGAAATAT	3	217
<q4>	GGGACATTTTAGAAAAGACAGAAATAT	11	793
<q5>	GGGACATTTTAGAAAAGACGTAAATAT	1	93
<q6>	GGGACATTTTAGAAAAGACGGAAATAT	1	70
<q7>	GGGACATTATAAAAAAGACAGAAATAT	3	198
<q8>	GGGACATTATAAAAAAGACATTATAAT	1	61
<q9>	GGGACATTATAAAAAAGACGTAAATAT	2	125
<q10>	GGGACATTATAAAAAAGACAGAATTAT	1	63
<q11>	GGGACATTATATAAAAGACAGAAATAT	2	144
<q12>	AGATCATTAGAGAAAAGACGTAAATAT	1	80
<q13>	GGGACATTTTAAAAAAGACAGAAATAT	1	58
<q14>	GGGCATTATAGAAAAAGACAAAAATAT	1	57
<q15>	GAGTCATTATAAAAAAGACAGAAATAT	1	63
<q16>	GGGTCATCAGAGAAAAGACAGAAATAT	3	268
<q17>	GGGTCATTATAAAAAAGACAGAAATAT	1	65
<q18>	GGGACATTTTAGAAAAGACAAAAATAT	1	66
<q19>	AGGACATTTTAGAAAAGACAGAAATAT	1	72
<q20>	GGGTCATTATAAAAAAGACAAAAATAT	1	50
		41	
